# A three-gene signature based on tumour microenvironment predicts overall survival of osteosarcoma in adolescents and young adults

**DOI:** 10.18632/aging.202170

**Published:** 2020-12-03

**Authors:** Chunkai Wen, Hongxue Wang, Han Wang, Hao Mo, Wuning Zhong, Jing Tang, Yongkui Lu, Wenxian Zhou, Aihua Tan, Yan Liu, Weimin Xie

**Affiliations:** 1 Department of Breast, Bone and Soft Tissue Oncology, the Affiliated Tumor Hospital of Guangxi Medical University, Nanning 530021, China; 2 Graduate School of Guangxi Medical University, Nanning 530021, China; 3 Department of Bone and Soft Tissue Tumor Surgery, the Affiliated Tumor Hospital of Guangxi Medical University, Nanning 530021, China

**Keywords:** osteosarcoma, prognosis, risk model, tumour microenvironment, TARGET

## Abstract

Evidences shows that immune and stroma related genes in the tumour microenvironment (TME) play a key regulator in the prognosis of Osteosarcomas (OSs). The purpose of this study was to develop a TME-related risk model for assessing the prognosis of OSs. 82 OSs cases aged ≤25 years from TARGET were divided into two groups according to the immune/stromal scores that were analyzed by the Estimate algorithm. The differentially expressed genes (DEGs) between the two groups were analyzed and 122 DEGs were revealed. Finally, three genes (COCH, MYOM2 and PDE1B) with the minimum AIC value were derived from 122 DEGs by multivariate cox analysis. The three-gene risk model (3-GRM) could distinguish patients with high risk from the training (TARGET) and validation (GSE21257) cohort. Furthermore, a nomogram model included 3-GRM score and clinical features were developed, with the AUC values in predicting 1, 3 and 5-year survival were 0.971, 0.853 and 0.818, respectively. In addition, in the high 3-GRM score group, the enrichment degrees of infiltrating immune cells were significantly lower and immune-related pathways were markedly suppressed. In summary, this model may be used as a marker to predict survival for OSs patients in adolescent and young adults.

## INTRODUCTION

Osteosarcomas (OCs) is the most common primary bone tumor in adolescent and young adults and the incidence is higher at age 15 to 19 years old [1]. OSs has a high potential to metastasize to lungs. Neoadjuvant chemotherapy-Surgical resection-Adjuvant chemotherapy (the so-called sandwich treatment mode) is the standard treatment for early and locally advanced OSs, which has significantly improved the prognosis of patients, with the 5-year survival has exceeded 60% [1, 2].

However, for advanced patients, the application of emerging therapies such as targeted therapy and immunotherapy (immune checkpoint inhibitors) is not optimistic, and the chemotherapeutic regimens included adriamycin, cisplatin, ifosfamide and high-dose methotrexate are still the main options [3, 4]. Unfortunately, about one-third of patients eventually failed due to drug resistance. In the past few decades, the 5-year survival of patients with metastasis at diagnosis or in relapse has not significantly improved, but still remains at only 20% [5, 6]. Therefore, it is remains a major goal for developing new therapy and valid signature to improve or predict the prognosis of OSs. Increasing evidence indicated that the biological behavior of tumor, such as invasion and metastasis, drug resistance and so on, depended not only on the inherent characteristics of tumor cells, but also on the composition and function of tumor microenvironment (TME) [7–9].

OSs has extremely significant heterogeneity, the landscape of driver mutations had proved few mutations recur with high frequency at the intra-tumor level of OSs, resulting in the loss of definite targets for therapy [10, 11]. On the other hand, and more importantly, TME was not only considered essential for the growth of osteosarcoma, but also regulated the proliferation, migration and metastasis of osteosarcoma cells, as well as drug resistance, immunosuppression and immune escape [5, 12]. The TME of OSs is a complex environment composed of active cells (such as tumor cells, stromal cells and immune cells, fibroblasts, etc.), vasculature, extracellular matrix (ECM), and a variety of factors [5, 13, 14]. Among them, the tumor-infiltrating immune cells (TIICs) and stromal cells are closely related to OSs cells by regulating various signal pathways via release of cytokines and other soluble factors, conversely stimulating and facilitating tumor cell metabolism and proliferation in all the stages of carcinogenesis [15–17]. Therefore, TME-related genomic analysis can help to find markers for assessing disease evolution and prognosis of OSs patients, and even for translation in both molecularly targeted therapy and personalized therapy [18, 19].

In the past decade, several methods have been invented to dissect the TME based on gene expression profiles. One of these, Estimate algorithm [20], designed by Yoshihara et al, was used to assess the score of immunity and stromal in the TME based on gene expression profiles, and it has been successfully applied to a variety of solid tumors, such as ovarian cancer [21], bladder cancer [22] and gastric cancer [23]. A similar approach, Cibersort [24], a new biological tool based on the deconvolution technique, can quantify the abundance of specific TIICs. Unfortunately, there are no definitive and clinically applicable prognostic markers for OSs. Thus, this study expects to establish a new predictive model based on TME-related genes and evaluate its effect in estimating the outcome of OSs, so as to provide a reference for further research in the future.

## RESULTS

### Correlation between immune/stromal score in TME and prognosis of OSs patients in adolescents and young adults

**Figure 1:**
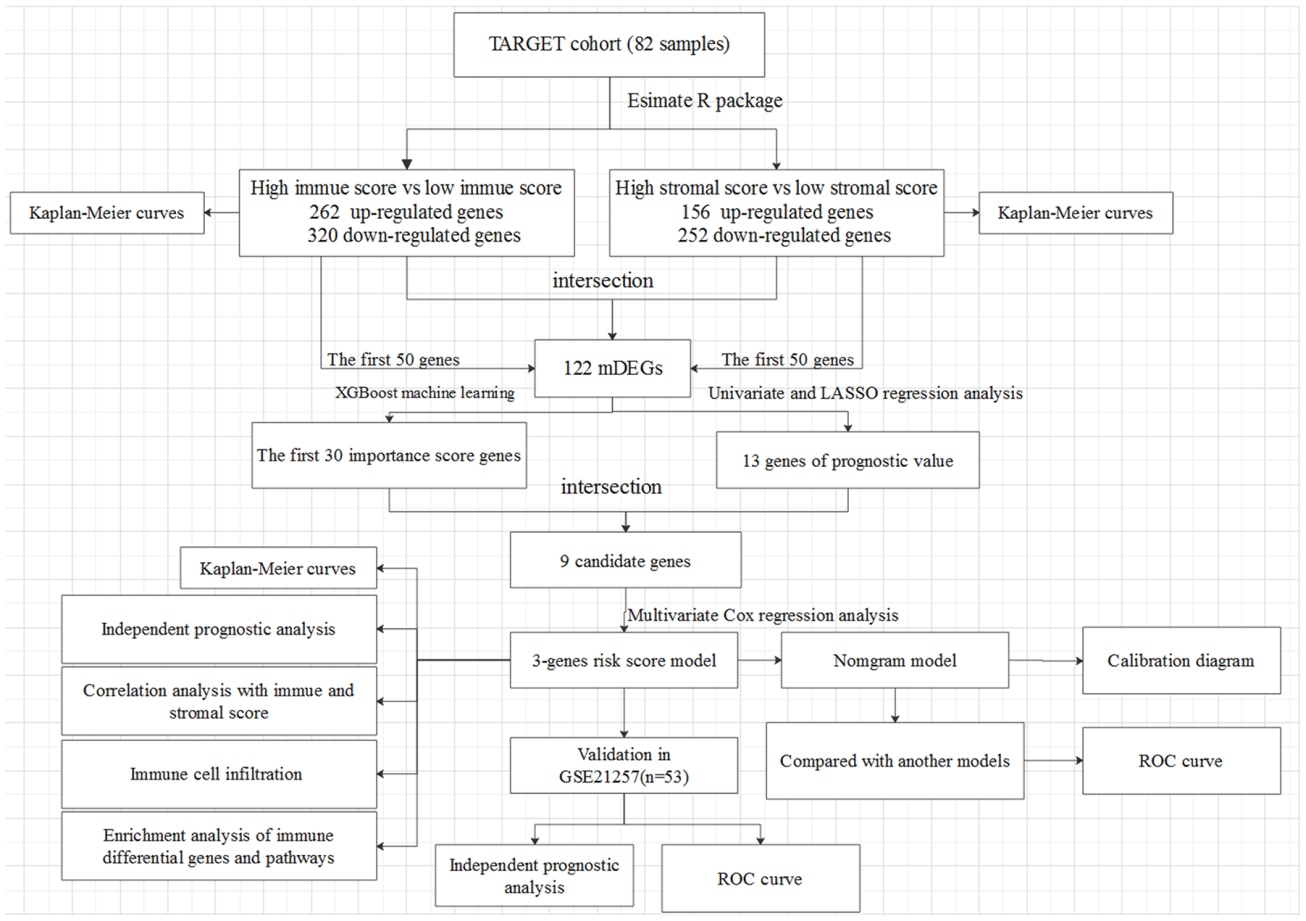
**The overall design of the present study.**

In this study, 82 OSs patients (≤ 25 years of age) from TARGET were enrolled, those with complete gene expression data of samples and clinical information of follow-up. The detailed clinical characteristics of patients were summarized in Supplementary Table 1. The flowchart of the analysis procedure was shown in Figure 1. Based on the normalized matrix data, the TME score was graded using the Estimate algorithm, and the results showed that the immune scores and stromal scores of all patients were ranged from -1508.04 to 2638.38 and from -695.66 to 1962.14, respectively.

**Figure 2:**
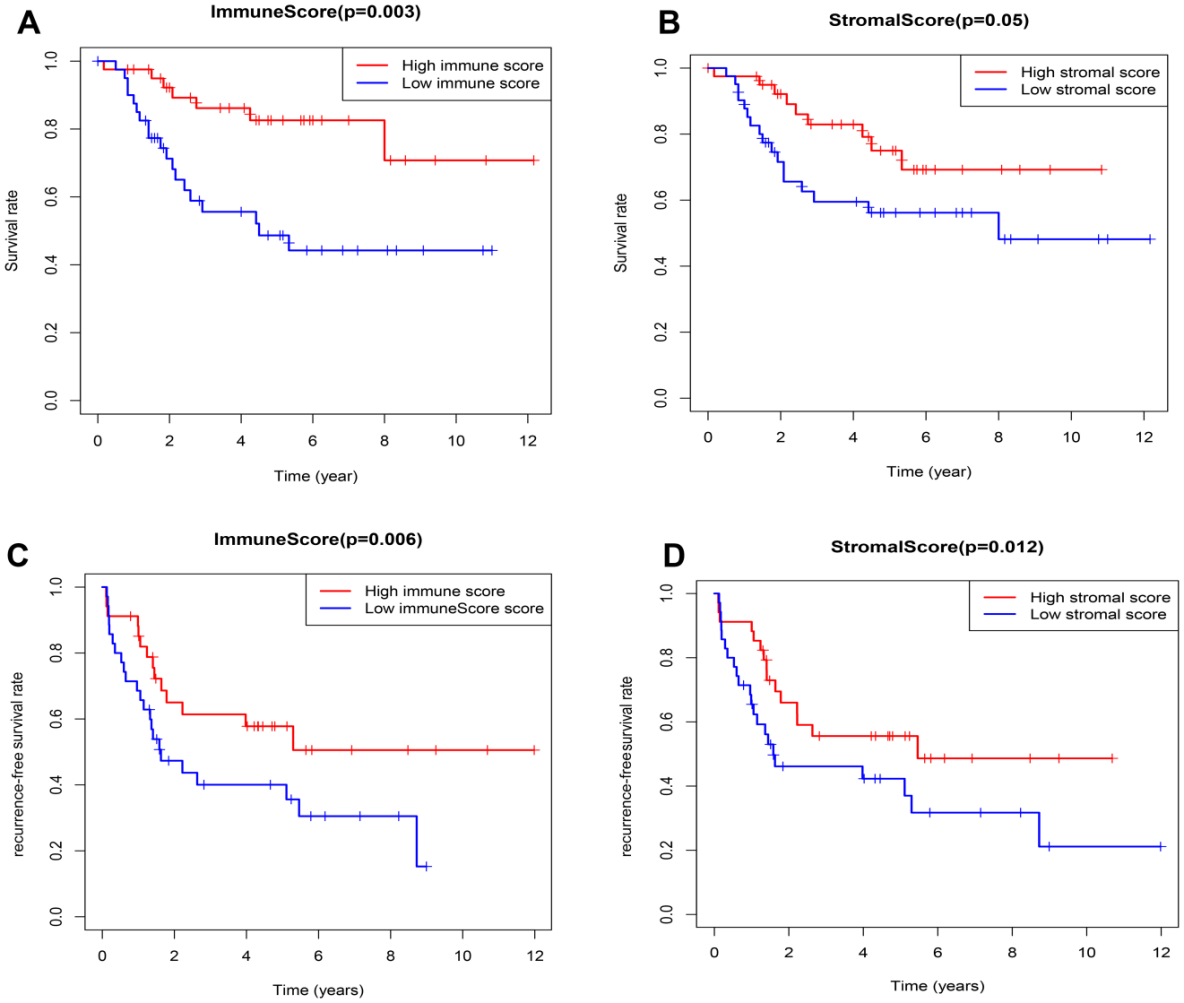
**Relationship between the TME-related immune/stromal score and survival of OSs patients.** (**A**) Immune score and overall survival rate. (**B**) Stromal score and overall survival rate. (**C**) Immune score and recurrence-free survival rate. (**D**) Stromal score and recurrence-free survival rate.

According to the median value of immune/stromal score, 82 OSs patients were divided into high and low score group. Kaplan-Meier (K-M) curves showed that the 5-year overall survival (OS) rates of patients with high and low immune scores were 82.6% and 48.7%, respectively (P=0.003) (Figure 2A). As well as, the 5-year recurrence-free survival (RFS) rates were 65% and 47.3%, respectively (P=0.006) (Figure 2C). Consistently, the 5-year OS rates of patients with high and low stromal scores were 65% and 47.3%, respectively, the P value approximately reached statistical difference (P=0.053) (Figure 2B), and the 5-year RFS rates were 66% and 46.1%, respectively (P=0.012) (Figure 2D), indicating that the TME-related immune and stromal score in the TME were significantly correlated with the survival.

### Identification of differentially expressed genes (DEGs)

**Figure 3:**
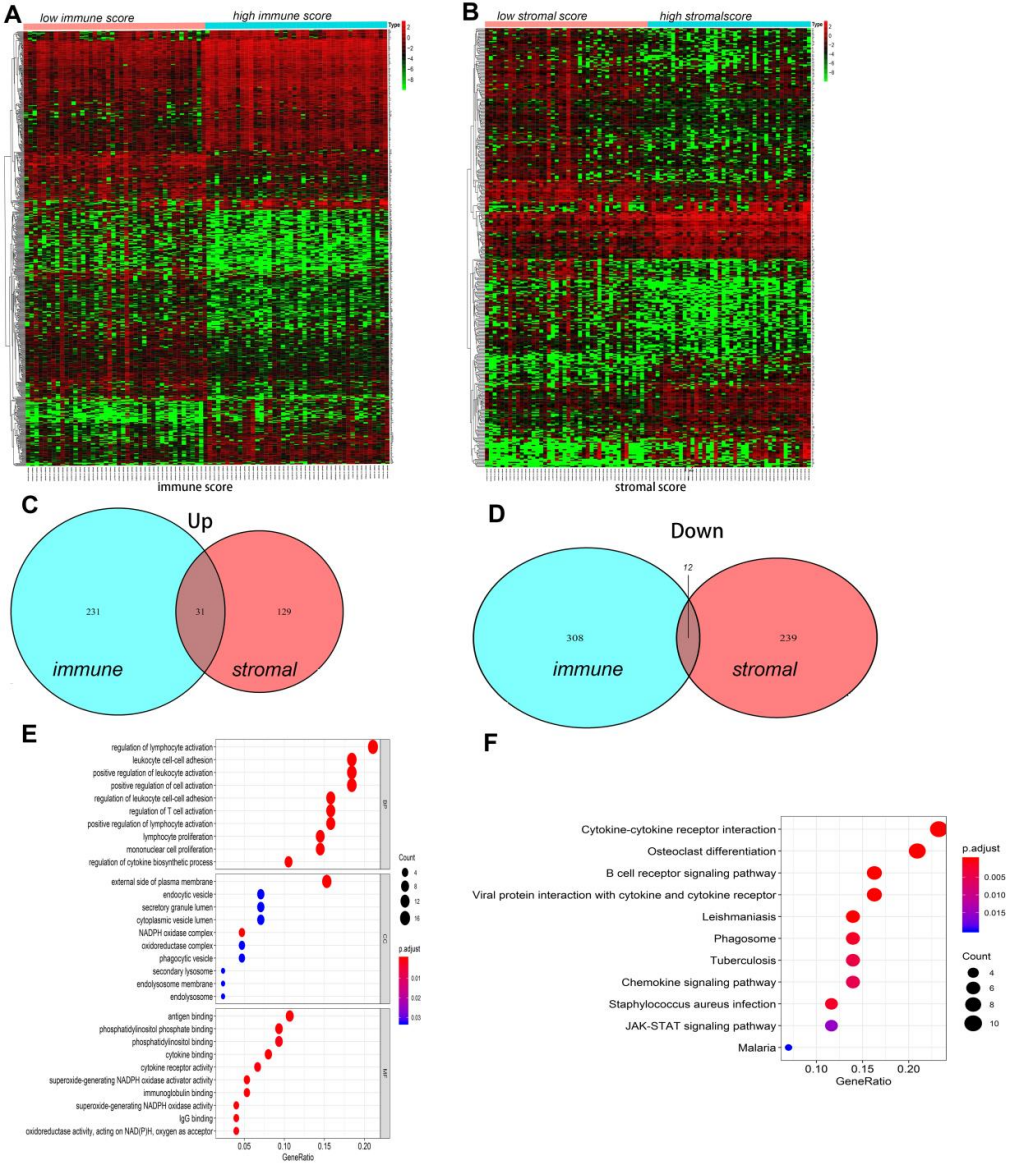
**The differences of genes expression and pathways enrichment based on immune scores and stromal scores.** (**A**, **B**) Heatmap of significantly differentially expressed genes based on immune and stromal scores. (**C**, **D**) Venn diagram analysis of aberrantly expressed genes based on immune and stromal scores in osteosarcoma. (**E**) GO analyses of the tmDEGs in the categories of biological processes (BP), cellular components (CC), and molecular functions (MF). (**F**) KEGG analysis of tmDEGs genes. tmDEGs, differentially expressed genes of the tumour microenvironment. GO, Gene Ontology; KEGG, Kyoto Encyclopedia of Genes and Genomes.

The DEGs between the high and low immune/stromal scores groups were obtained based on gene expression profiles, and a fold-change > 1 or < -1 and adjusted P<0.05 were used as criterions for screening DEGs. Heatmaps showed the distinct gene expression profiles from the two groups in Figure 3A, 3B. In the high immune score group, 320 genes were down-regulated and 262 genes were up-regulated (Supplementary Tables 2A, 2B). Meanwhile, the high stromal score group had 252 genes down-regulated and 160 genes up-regulated (Supplementary Tables 3A, 3B). Furthermore, Venn diagram was used to find the common-DEGs in the two groups (Figure 3C, 3D), showing that 31 genes were generally up-regulated and 12 genes were markedly down-regulated (Supplementary Table 4). The top 50 genes with the most significant difference between the high and low immune score groups, and the high and low stromal score groups, respectively, and other differentially expressed genes crossing with Venn diagram were all included. Finally, a total of 122 genes, defined as the TME-related differentially expressed genes (tmDEGs), were included in the subsequent functional classification analysis.

GO(Gene Ontology) Term analysis confirmed that the tmDEGs mostly involved in regulation of leukocyte activation, leukocyte cell-cell adhesion, positive regulation of cell activation in biological processes (BP), external side of plasma membrane, endocytic vesicel, secretory granule lumen in cellular components (CC), antigen binding, phosphatidylinositol phosphate binding, phosphatidylinositol binding in molecular functions (MF) (Figure 3E). KEGG enrichment analysis showed that tmDEGs mainly enriched in the osteoclast differentiation, B cell receptor signaling pathway cell receptor interaction, viral protein interaction with cytokine and cytokine receptor, cytokine-cytokine receptor interaction and JAK-STAT signaling pathway (Figure 3F).

These results indicated that the immune score and stromal score could all better reflect the main immune landscapes of TME, showing the activation of immune cells and stromal cells, and the expression of various signal pathways in OSs tissues.

### Construction and evaluation of the risk score model

**Figure 4:**
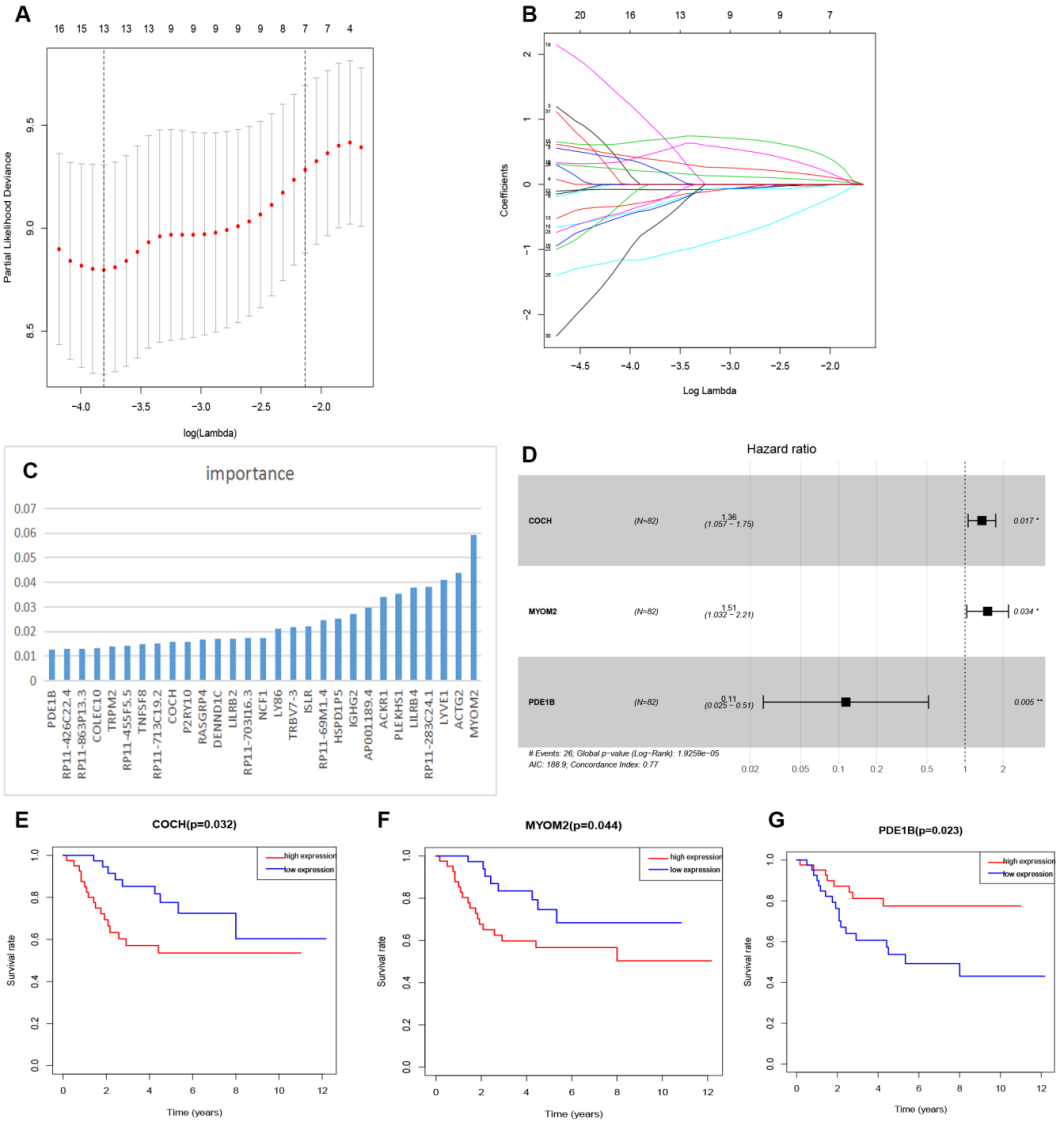
**Feature selection of risk score model.** (**A**) Selection of tuning parameters in the Lasso regression analysis based on 1,000 cross-validations. (**B**) Lasso regression analysis coefficients. (**C**) The importance of XGBoost machine learning screening the top 30 genes. (**D**) Multivariate analysis of 3 genes (COCH, MYOM2, PDE1B) and establishment of the regression equation. (**E**–**G**) Kaplan-Meier curve analysis of the relationship between the expression levels of COCH, MYOM2 and PDE1B, respectively, and the prognosis of OSs patients.

The relationship between tmDEGs and prognosis of 82 OSs patients from TARGET as above was analyzed. The univariate cox regression analysis showed that 32 of 122 tmDEGs were significantly correlated with OS of patients (P<0.05) (Supplementary Table 5). Then Lasso regression analysis was performed on these 32 genes, and 13 genes were screened as candidate genes through relative regression coefficient (Figure 4A, 4B).

And then the top 30 genes, identified from 122 tmDEGs by XGBoost machine learning according to the order of importance (Figure 4C), were crossed with these 13 genes selected by Lasso regression analysis, and 9 candidate genes were obtained.

Finally, a multivariate cox analysis was performed, and three genes with the minimum Akaike's Information Criterion (AIC) value of 188.9 were identified to construct a risk model (Figure 4D), which was called three-gene risk model (3-GRM). The C index of model was 0.77 (Se=0.042, 95% CI, 0.688~0.852).

According to the median value of 3-GRM score, patients were divided into high score group and low score group. The expression levels of these three genes were significantly correlated with survival of OSs patients from dataset TARGET, among which, Cochlin (COCH) and Myomesin2 (MYOM2), both overexpressed in the high 3-GRM score group, were associated with poor prognosis. However, PD1EB was significantly down-regulated in high 3-GRM score group, was related to favorable prognosis (Figure 4E–4G). Based on relative coefficients in multivariable cox regression analysis, the scoring formula of 3-GRM was as follows: risk score = [0.30×COCH expression level]+ [0.41×MYOM2 expression level]-[2.1×PDE1B expression level].

**Figure 5:**
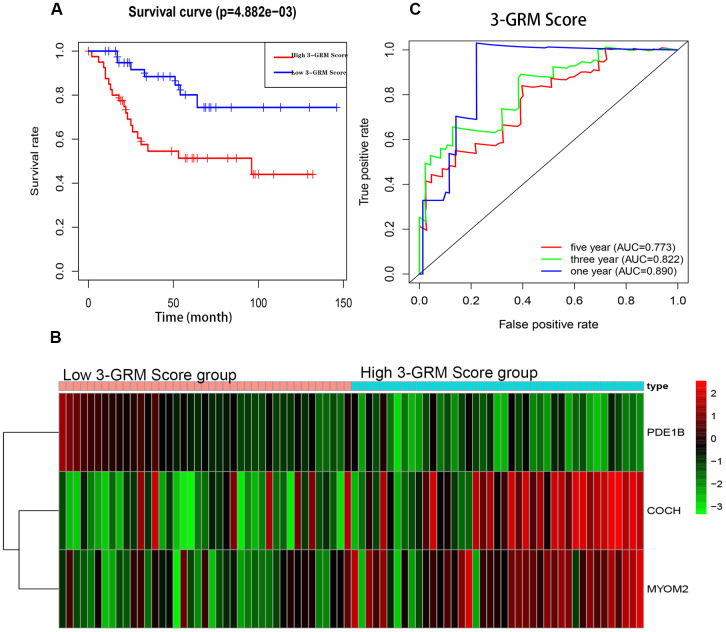
**Prognostic analysis of the 3-genes risk model (3-GRM) in TARGET.** (**A**) Prognostic analysis between the high and low 3-GRM score group. (**B**) Differences of COCH, MYOM2 and PDE1B expression levels between the high and low 3-GRM score group. (**C**) Time-dependent ROC curve analysis of the 3-GRM.

The K-M analysis showed that the OS of high 3-GRM score group was significantly lower than that of low score group (P<0.05) (Figure 5A), and the 5-year survival rates of the high and low score group were 51.3% and 80.1%, respectively (P=0.000). The distribution of genes expression data in the subgroup of difference risk scores were shown in Figure 5B. The prediction ability of 3-GRM was evaluated by calculating the area under the ROC curve (AUC). The AUC values in predicting 1, 3 and 5-year of survival rate were 0.890, 0.822 and 0.773, respectively, indicating that 3-GRM had a good prediction effect (Figure 5C).

### High score of 3-GRM was an independent prognostic factor for OSs patients in adolescent and young adults

**Figure 6:**
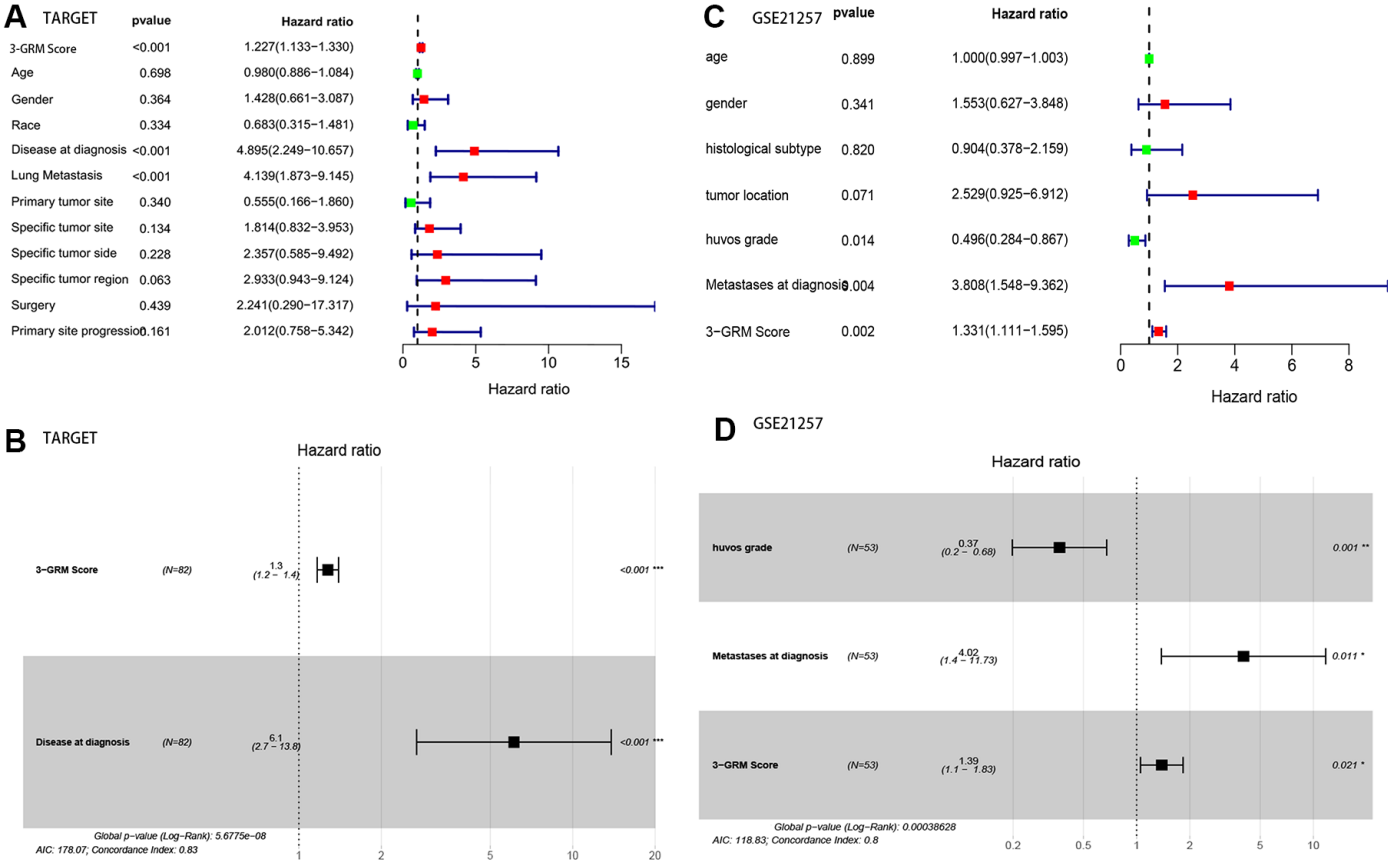
**Analysis of prognostic factors of OSs patients.** (**A, B**) Univariate and multivariate regression analysis of the relation between the 3-GRM and clinicopathological features in TARGET. (**C, D**) Univariate and multivariate regression analysis of the relation between the 3- GRM and clinicopathological features in GSE21257.

Based on the clinical data and survival information of 82 patients, univariate cox regression analysis showed that the high score of 3-GRM, metastasis at diagnosis and lung metastasis were the risk factors affecting prognosis, but age, gender, race, primary site, specific site of primary tumor, and surgical method were not related to outcome of patients (Figure 6A). As in the TARGET database (as above), 95% of patients (20/21) with metastasis at diagnosis had lung metastasis, so these two factors were classified as having metastasis at diagnosis for further analysis. Multivariate cox regression analysis confirmed that high 3-GRM score and metastasis at diagnosis were independent risk factors of OSs patients (Figure 6B). K-M analysis showed that the 5-year survival rates of cases with metastases and without metastases at diagnosis were 31.3% and 77.4%, respectively (P<0.001, data not shown).

### Validate the new risk model (3-GRM) in GEO dataset

To verify the robustness of 3-GRM, another independent dataset GSE21257 (n=53) (Table 1) from the GEO database was used. According to the risk scoring formula as above, the patients in GSE21257 dataset were divided into two groups, with 28 cases in high 3-GRM score group and 25 cases in low 3-GRM score group.

**Table 1:** Clinical baseline data and score grouping of 53 patients with OSs in GES21257 dataset.

**Clinical features**	**Cases, n (%)**	**High 3-GRM score group, n (%)**	**Low 3-GRM score group, n (%)**	P*
**Patients(n)**	53	28	25	
**Median age(range)**	17 (14-19)	16.5(14-19)	17(13-19)	0.71
**Gender**				
Female	19 (35.8)	7 (25.0)	12 (48.0)	0.08
Male	34 (64.2)	21 (75.0)	13 (52.0)	
**Histological Subtype**				
Osteoblastic	32 (60.4)	19 (67.9)	13 (52.0)	0.24
Others	21 (39.6)	9 (32.1)	12 (48.0)	
**Tumor location**				
Lower limb	44 (83.0)	23 (82.1)	21 (87.5)	0.88
upper limb	8 (15.1)	5 (17.9)	3 (12.5)	
Unknow	1(1.89)	-	-	
**Huvos grade**				
I-II	29 (54.7)	14 (58.3)	15 (65.2)	0.85
III-IV	18 (34.0)	10 (41.7)	8 (34.8)	
Unknow	6(11.3)	-	-	
**Metastases at diagnosis**				
No	39 (73.6)	20 (71.4)	19 (76.0)	0.95
Yes	14 (26.4)	8 (28.6)	6 (24.0)	

*Comparison of high 3-GRM score group and low 3-GRM score group.

**Figure 7:**
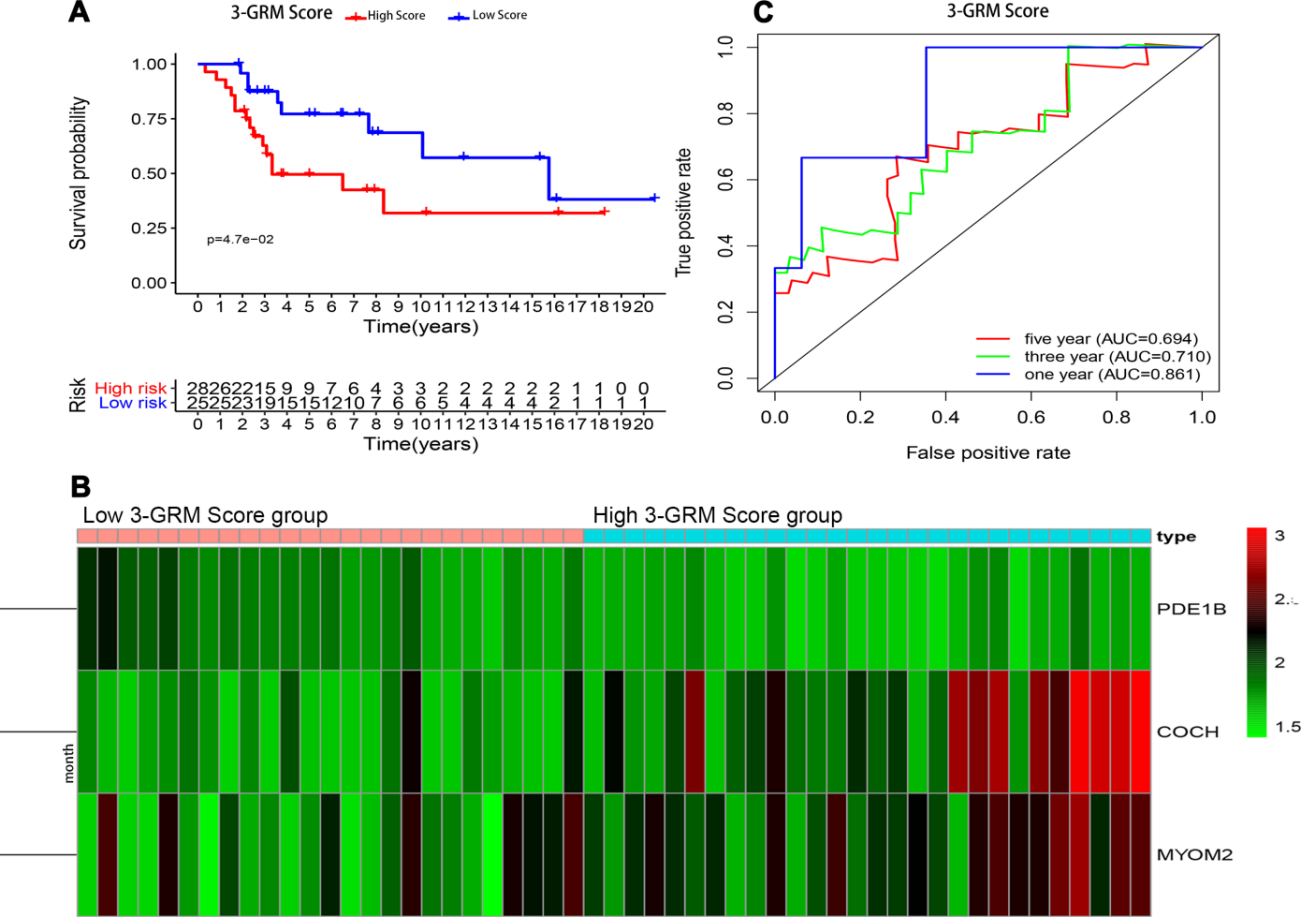
**Prognostic analysis of the 3-GRM in GSE21257.** (**A**) Prognostic analysis between the high and low 3-GRM score group. (**B**) Differences of COCH, MYOM2 and PDE1B expression levels between the high and low 3-GRM score group. (**C**) Time-dependent ROC curve analysis of the 3-GRM.

Consistent with previous results, this results also confirmed that patients with high 3-GRM score had significantly shorter OS than those with low scores (Figure 7A). The AUC values of the 3-GRM in predicting 1, 3 and 5-year of survival rate were 0.861, 0.710 and 0.694, respectively (Figure 7B). The Figure 7C shows the distribution of the 3-GRM scores and gene expression data in the validation cohorts. At the same time, univariate (Figure 6C) and multivariate (Figure 6D) cox analysis found that huvos grade (histological response grade after chemotherapy) [25], metastasis at diagnosis and the high 3-GRM score were also independent prognostic factors for OSs patients in the GSE21257 dataset, confirming that the 3-GRM was robust and effective.

### The relationship between 3-GRM score and TME score, and the difference of TIICs between the high and low 3-GRM score groups

**Figure 8:**
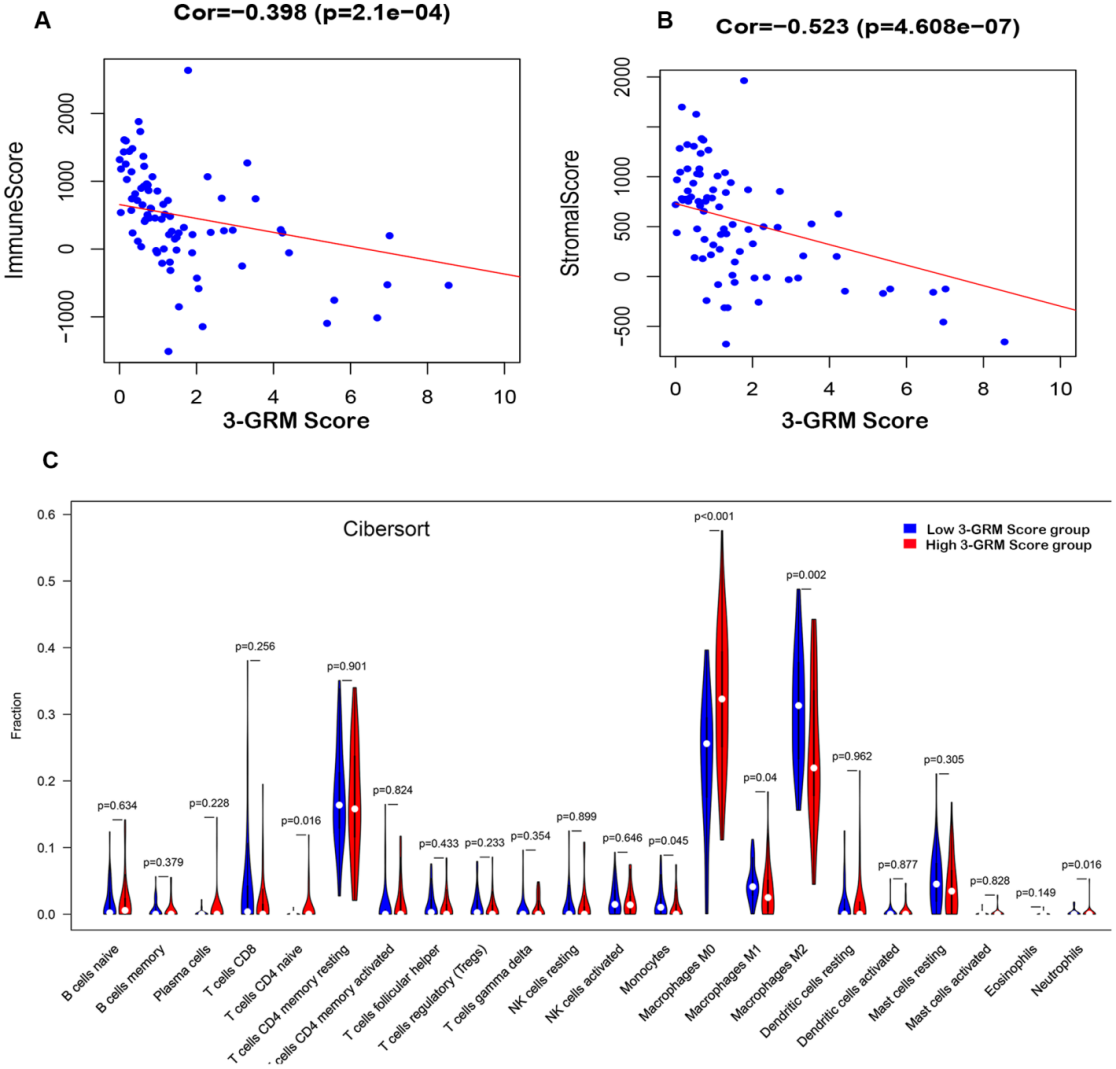
**Relationship between the 3-GRM score and the immune/stromal score, and the level of TIICs.** (**A**) The relationship between the 3-GRM score and the immune score by Estimate. (**B**) The relationship between the 3-GRM score and the stromal score. (**C**) Comparison of the levels of TIICs between high 3-GRM score group and low 3-GRM score group by Cibersort. The horizontal axis represents the type of TIICs, and the vertical axis represents the relative percentage. NS, no significance; TIICs, tumour-infiltrating immune cells.

Based on TARGET database, the analysis results found that the 3-GRM score was both significantly correlated with the TME-related immunity score (R=0.398, P<0.01) and the stromal score (R=0.523, P<0.01) (Figure 8A, 8B), respectively, indicating that the higher the 3-GRM score, the lower the immune score and stromal score.

Cibersort algorithm analysis revealed that the most common TIICs in the specimens of 82 OSs patients were macrophages M2(27.8±10.14)%, macrophages M0 (27.63±11.35)% and CD4+ memory T cells (17.08±7.86)%, accounting for more than 70%. In addition, the proportion of macrophages M0 in the high 3-GRM score group was significantly higher than that in the low score group (P<0.001), while the ratios of monocytes (P=0.05), macrophages M1 (P=0.04) and macrophages M2 (P=0.002) were significantly lower than those in the low score group (Figure 8C).

Furthermore, 64 kinds of TME-related cells were evaluated by Xcell, and the results were consistent with that of Cibersort algorithm. Compared with the low 3-GRM score group, the enrichment degree of TIICs (such as “aDC”, “cDC”, “iDC”, "macrophage M1 and M2", "endothelial cells"and "MSC") were significantly lower, while "muscle cells" and "skeletal muscle cells" were significantly enrichment in the high 3-GRM score group (all P<0.01)(Supplementary Figure 1).

### Comparison of immune-related signal pathways between the high and low score group

**Figure 9:**
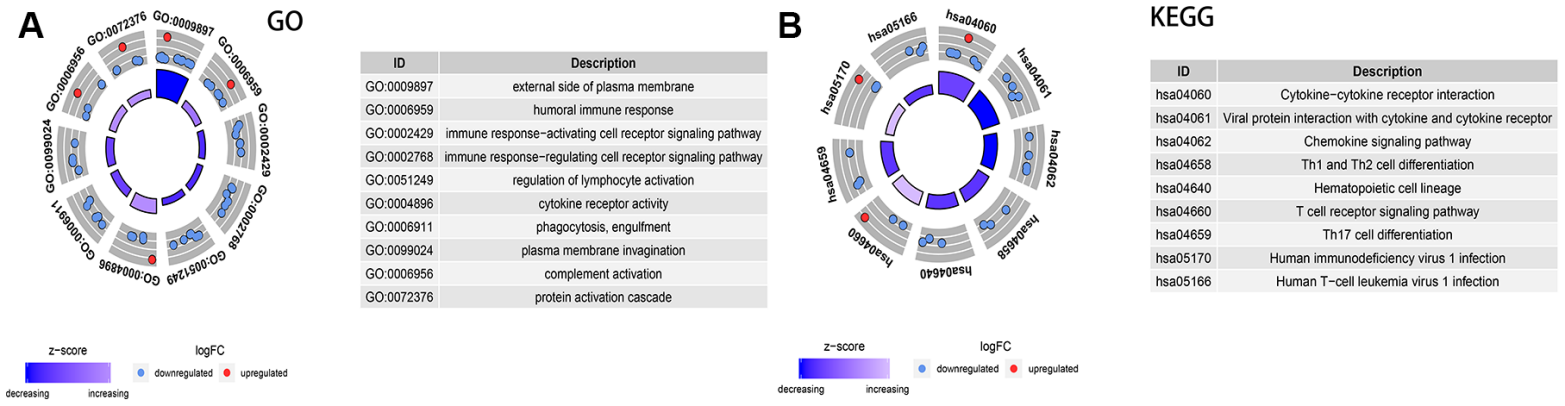
**Analysis of the expression of immune-related differential genes, enrichment of immune-related pathways in high and low 3-GRD score group.** (**A**) GO analyses of the immune-related differentially expressed genes in the categories of biological processes (BP), cellular components (CC), and molecular functions (MF). (**B**) KEGG analysis of the immune-related signaling pathways. GO, Gene Ontology; KEGG, Kyoto Encyclopedia of Genes and Genomes.

A total of 1,811 immune-related genes were obtained from the website of IMMPORT (http://www.immport.org/) for differential analysis, with screening criteria were fold-changed >1 or <-1 and adjusted P<0.05. The results showed that there were 31 DEGs between the two groups (Supplementary Table 6). Identified by GO terms, these DEGs were involved in external side of plasma membrane (CC), humoral immune response(BP), immune response-activating/regulating cell receptor signaling pathway(BP), regulation of lymphocyte activation (BP) and cytokine receptor activity(MF) (all adjusted P<0.01) (Figure 9A).

The enrichment analysis of KEGG pathway explored that of immune-related signaling pathways were markedly down-regulated in the high 3-GRM score group, including cytokine−cytokine receptor interaction, viral protein interaction with cytokine and cytokine receptor, chemokine signaling pathway, Th1/Th2 and Th17 cell differentiation, and T cell receptor signaling pathway (all adjusted P<0.01) (Figure 9B).

### Establishment and evaluation of nomogram model for survival based on 3-GRM scoring and clinicopathological features

**Figure 10:**
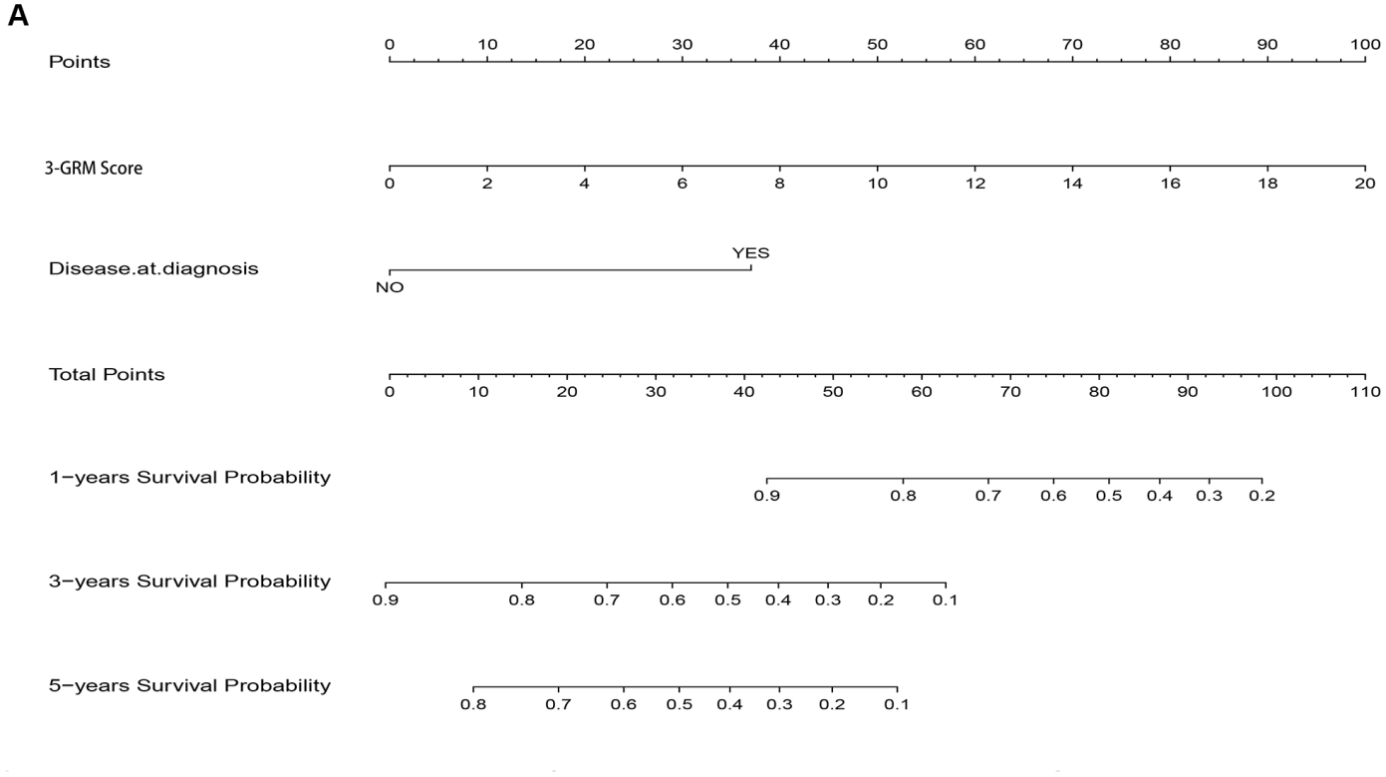
**Nomogram model for predicting the outcome of OSs patients.** (**A**) Nomogram model for predicting the probability of 1, 3, and 5-year overall survival (OS) for adolescent and young adults with osteosarcoma.

As the result of multivariate cox regression analysis showing there were two prognostic indicators of OSs (Figure 6B), a nomogram model included 3-GRM score and clinicopathological features (such as metastasis status at diagnosis) were developed (Figure 10A), with the index C was 0.825. The calibration plot for the possibility of 1, 3and 5-year survival showed good agreement between the prediction by risk score and actual observations (Supplementary Figure 2A–2C). The AUC values of nomogram model in predicting 1, 3 and 5-year survival reached to 0.971, 0.853 and 0.818, respectively. In addition, the nomogram model was also verified in GSE21257 dataset, with the AUC values in predicting 1, 3 and 5-year survival were 0.781, 0.840 and 0.795, respectively. The predicted results of the calibration plot were also consistent with the actual observation (Supplementary Figure 3A, 3B).

Recently, prognosis models had been established and used to predict the prognosis of OSs in several studies [26–29]. In our study, the AUC value was used for evaluating the prediction effect of varies models. As shown in Table 2, the calculate effects of nomogram model were always better than those of the 3-GRM and other models [26–29].

**Table 2:** Comparison of OSs-related prognostic models in TARGET database.

**OSs related prognostic model**	**Constitution**	**1-year AUC value**	**3-year AUC value**	**5-year AUC value**
Nomogram model	3-GRM + metastasis at diagnosis	0.971	0.853	0.818
3-GRM	3-gene signature	0.89	0.822	0.773
Zhang,et al.(2020) [26]	3-gene signature	0.643	0.781	0.809
Li,et al.(2020) [27]	4-gene signature	0.644	0.714	0.738
Liu,et al.(2020) [28]	2-gene signature	-	0.71	0.72
Shi,et al.(2020) [29]	3-gene signature	0.914	0.849	0.822

Note: AUC, area under the ROC curve; OSs, osteosarcomas; 3-GRM, three-gene risk model.

## DISCUSSION

OSs has a relatively high incidence in the second decade of life, especially at ages 15-19 years [1]. Indeed, genetic investigations have demonstrated the paucity of mutations more specifically involving signal transduction pathways in adolescent and young adults sarcomas compared with adults. The result highlights a major difference between adolescent and young adults and adult. In other words, there may be different immune landscapes in adult osteosarcoma and childhood osteosarcoma [30]. In addition, The clinicopathologic features that may influence the prognosis of OSs patients include age, metastasis at diagnosis, lesion location, and degree of tumor necrosis (Huvos grade) after neoadjuvant chemotherapy, etc. [1, 2]. However, age has not been taken into account in many prognostic model-related studies [26–29]. Therefore, patients under 25 years old were enrolled in this study, and it is hoped that the model could more accurately predict the prognosis of such population.

The TME is an extremely complex system that has not been clearly elucidated [5, 31].

Currently, exploratory researches involved in TME include the molecular events of regulation between OSs and non-tumor cells, the genomic drivers of disease progression, prediction model that best reflects the clinical outcome and its transformational applications [19, 32].

Evidences shows that immune and stromal related genes in TME may play key regulatory roles in the prognosis of OSs. The interaction between Programmed Cell Death Protein 1 (PD-1) and its Ligand 1(PD-L1) is critical for tumor cells survival. PD-1 is expressed in T lymphocytes, while PD-L1 is overexpressed in tumor cells. PD-1 binding with PD-L1 can interfere with and inhibit the ability of T cells to kill cancer cells [33]. The pooled results of a meta-analysis showed that PD-L1 overexpression could predict poor OS (HR 1.45, 95% CI: 1.11-1.90, P<0.01), metastasis-free survival (HR1.58, 95% CI: 1.14-2.19, P<0.01) and event-free survival (HR 2.82, 95% CI: 1.69-4.71, P<0.01) in OSs, and was also significantly correlated with a higher rate of tumor metastasis (OR 2.95, 95% CI: 1.32-6.60, P< 0.01) [34]. CXCL12 (also known as stromal cell-derived factor 1, SDF-1), one of the chemokine protein family, is the main regulator of cell trafficking, affecting both tumor cells and white blood cells [35]. Overexpression of CXCL12 was positively correlated with the number of tumor infiltrating lymphocytes and the better survival rate of OSs patients [36].

Among various methods, the TME-related gene expression profiling, based on immune score and stromal score obtained by Estimate algorithm, is a common and effective method to evaluate tumor microenvironment [20–23]. In this study, we analyzed the TME characteristics of OSs patients under 25 years old from TARGET dataset and its relationship with patient prognosis. According to the immune score and stromal score of TME, DEGs were obtained to construct a three-gene risk model (3-GRM). The results of validation were consistent in two independent databases, which confirmed that this 3-GRM was efficient and robust and the high score of 3-GRM was an independent prognostic factor for OSs patients. Further analysis found that the 3-GRM score was all strongly negatively correlated with the immune score and stromal score, respectively, indicating the model could effectively identify the TME status of OSs. Specifically, in this model, both COCH and MYOM2 were overexpressed in the high 3-GRM score group and led to poor prognosis. However, PD1EB was significantly down-regulated in the high-scoring group, and analysis showed (Figure 4E–4G) that its overexpression was beneficial to survival. As far as we know, the prognostic risk model based on these three genes has not reported before.

COCH gene is highly expressed in the sensory organs (inner ear and eye), lymph nodes and spleen [37]. It plays an important role in maintaining the shape of cells [38]. And Cochlin, which encoded by COCH, and its domains are the main non-collagenous components of extracellular matrix (ECM) and have high affinity for multi-type collagens [38, 39]. A study shown that COCH was a transition zone-specific genes and was also stroma-specific of the prostate, and involved in the occurrence of prostatic hyperplasia and prostate cancer [40]. Up-expression of COCH was directly related to the stage progression of clear cell renal cell carcinoma (ccRCC) [41]. Interestingly, research indicated that in the circulation, the LCCL domains, one N-terminal factor C homology of COCH, could signal the innate immune cells and amplify the cytokine response in the form of glycosylated polypeptides through unknown pathways [42]. Similar, Cochlin, secreted from follicular dendritic cells in the spleen, was crucial for systemic immune response against bacterial infection by induces secretion of cytokines (IL-1βand IL-6) and enhances the recruitment of immune cells (neutrophils and macrophages) [37, 43, 44].

MYOM2 (myomesin 2) is a type of muscle fiber related protein and is an essential component of cytoskeleton [45, 46]. It is also the main component of the M-bands, which are located in the center of the sarcomere and are essential for the stability of sarcomere contraction [45, 47]. Study had revealed that in relapsed/refractory diffuse large B-cell lymphoma patients, late oncogenic events was composed of clonally represented recurrent mutations/gene alterations including MYOM2 [48]. One report showed that MYOM2 was the only significantly up-regulated gene in localized invasive periodontitis, suggesting that it was associated with inflammation [49]. Tumor necrosis factor (TNF) blockers have a high efficacy in treating Ankylosing Spondylitis (AS), a study reported that IgG Galactosylation status combined with MYOM2 rs2294066 polymorphism (T allele in rs2294066 leads to MYOM2 overexpression) could precisely predicts anti-TNF response of AS, indicating that MYOM2 might associated with the pharmacology of TNF blockers [50]. In addition, the results of genomic screening represented MYOM2 mutations was probably causative for arthrogryposis [51].

PDE1B (Phosphodiesterase 1B) gene is located on chromosomes 12q13 and is a member of the cyclic nucleotide phosphodiesterase (PDE) family [52]. Interestingly, studies show that PDE1B may play a regulatory role in differentiation of multiple immune cell types via degrading intracellular levels of cAMP and cGMP [53, 54]. Exploration shown that granulocyte macrophage colony stimulating factor (GM-CSF) could shift the differentiation from a macrophage to a dendritic cell phenotype and also up-regulated PDE1B.Yet, in the presence of GM-CSF, IL-4 treatment suppressed the up-regulation of PDE1B2 (one of PDE1B variants with unique N-terminal sequences) [55]. Further research found that inhibiting PDE1B2 up-regulation did not prevent HL-60 cells differentiation, but could change some aspects of macrophage like phenotype, such as increased cell proliferation, phagocytosis and leukocyte adhesion molecule CD11b expression, accompanied by a lower basal levels of cAMP in cells [56]. Moreover, PDE1B2 might involve in the occurrence and prognosis of breast cancer [57, 58].

Regrettably, the mechanism by which these three genes affect the prognosis of patients with OSs is still unclear, and it is worth exploring in the future. We speculate that COCH overexpression may induce the secretion of multiple cytokines and enhance the recruitment of immune cells, leading to the imbalance of the local inflammatory environment of TME and mediating the immune escape of tumor cells [59–61]. Similarly, MYOM2 overexpression may also mediate the malignant phenotype of tumor cells by affecting the inflammatory response of TME [49–51]. However, under the mediation of cytokines such as GM-CSF, the up-regulation of PD1EB may promote the differentiation of macrophages into M1 subtype macrophages, thereby enhancing the anti-tumor immune response and helping to improve the prognosis of patients [53–56, 62, 63].

Moreover, we also discussed the properties of microenvironmental cell levels and immune-related signal pathways between the high 3-GRM group and the low 3-GRM group. In general, the degree of enrichment of immune infiltrating cells in the high score group was significantly reduced, and immune regulation-related signal pathways such as cytokine interaction signaling pathways and T cell receptor signaling pathways were significantly down-regulated, indicating that there were general immunosuppression in the TME, which might affect the prognosis of patients. Several important studies identified that there was a complex associations between TIICs and cancer survival. On the one hand, the characteristics of TIICs, including the density, composition and activation of immune cells, were closely related to the response of immunotherapy and chemotherapy [64, 65]. The presence of either a pre-existing or induced immune response might indicate a more favourable prognosis [66]. On the other hand, OSs cells could regulate the recruitment and differentiation of immune infiltrating cells (TIICs) to establish a local immune tolerance environment that was conducive to tumor growth, drug resistance and metastasis. OSs cells also could control the T-lymphocyte responses via the PD-1/PDL-1 system to affect the balance between M1 and M2 macrophage subtypes, then leading to immune tolerance [58, 59]. A study used whole genome, T cell receptor sequencing and other methods to analyze the immune status of OSs, and confirmed that there were likely multiple immune-suppressive features in OSs, which might lead to poor response to immune checkpoint inhibitors and neoadjuvant chemotherapy [67].

In order to further comprehensively explore the impact of potential factors, such as 3-GRM score, clinicopathological characteristics, huvos grade, etc., on the prognosis of patients, this study established a nomogram model, which composed of 3-GRM scores and with metastasis at diagnosis. The verification analysis in two independent datasets identified that the nomogram model had higher prediction efficiency than 3-GRM and other models [26–29]. This nomogram model was consistent with the multivariate analysis results of several large-scale clinical studies that the most adverse factors for prognosis were pulmonary metastases at diagnosis [68].

However, our research also has certain limitations. First of all, due to the limited number of cases and clinical data available in the data set, this study cannot analyze the correlation between the 3-GRM and clinical staging, tumor location, histological grade and other factors. Secondly, the role and mechanism of the three prognostic genes in OSs need to be further explored. Third, the 3-GRM needs to be further validated in multi-center clinical trials and prospective studies.

In summary, this study aimed at the microenvironment of OSs and obtained a risk model (3-GRM) consisting of three immune/stroma-related genes through strict classification and screening criteria. The verification results proved that the model had high sensitivity and specificity, and good robustness. In addition, combining the 3-GRM score with the patient's clinical characteristics might further improve the prediction efficiency. Therefore, this 3-GRM could be used as a marker to predict the outcome for OSs patients in adolescent and young adults.

## MATERIALS AND METHODS

### Data preparation

The gene expression profiles and clinical data of TARGET osteosarcoma patients were downloaded from the UCSC Xena website (http://xena.ucsc.edu/). Of those, a total of 82 patients under 25 years old and survive for more than 1 month were enrolled. The GSE21257 database was served as a validation, including 53 OSs patients and their clinical information (age, gender, histological subtype and huvos grade, tumor location, metastasis at diagnosis, and survival). All samples were obtained from primary lesion, and gene expression profiling were detected by microarray. The expression matrix data for the validation set GSE21257 was downloaded from the GEO database (https://www.ncbi.nlm.nih.gov/geo/), R package “limma” was used for quality control and normalization [69]. Gene expression value with multiple probes for genes was calculated as the average of the probes.

### Identification of DEGs related to the immune and stromal score of the TME

The immune and stromal scores were calculated based on mRNA expression profile by “estimate” package (http://r-forge.rproject.org) [20]. According to the median of scores, 82 OSs patients from TARGET dataset were divided into two groups: high and low immune/stromal score group. The differential expression genes of the TME (tmDEGs) between these two groups were identified with “limma” in R package (version 3.6.1; https://www.rproject.org/) [69]. The filtering criteria were the fold-change> 1 or <-1 and adjusted P<0.05.

### Enrichment analysis of DEGs

The potential functional enrichment of tmDEGs and immune-related differential genes were analyzed by the “clusterprofile” in R [70]. Functional enrichment included gene ontology (GO) categories in biological processes (BP), molecular functions (MF), cellular components (CC) and Kyoto Encyclopedia of Gene and Genome enriched by (KEGG) Pathway. The threshold of false detection rate (FDR) < 0.05 was considered significant.

### Construction of risk model of Oss

The genes related to survival of OSs patients were screened by univariate cox and lasso regression, and Xgboost was used to further narrow the screening range, then multivariate cox regression analysis was used to determine the genes used to establish the prognosis model. The Lasso regression was analyzed using "glmnet" R software package. XGBoost machine learning [71], used Anaconda software, and its script was from MichaelMW (github.com/MichaelMW/bnfo.course). The formula of risk scoring was as follows: risk score= [0.30×COCH expression level]+[0.41×MYOM2 expression level]-[2.1×PDE1B expression level]. In this paper, the evaluation of the 3-GRM involved ROC curve. “Survival ROC” R software package was used to visualize the specificity and sensitivity of the 3-GRM.

### Enrichment analysis of cells related to TME

We used two bioinformatics methods to analyze cells in TME, in which, Cibersort (https://cibersortx.stanford.edu) calculated the proportion of 22 kinds of TIICs in microenvironment based on deconvolution (Figure 9A) [24]; XCELL (https://xcell.ucsf.edu/) calculated the independent enrichment scores of 64 kinds of immune and stromal cells based on gene enrichment(Figure 9B) [72]. We further applied the Wilcox test to compare the differences of cells characteristics between high score and low score groups.

### Construction and validation of the nomogram model

Using "RMS" in R to draw the nomograms of 1, 3, 5-year OS of OSs patients [73]. 3-GRM score and metastasis at diagnosis, which were two independent prognostic factors identified by multivariate cox regression analysis, were incorporated into the nomogram model. “Bootstrap” obtained 1, 3, 5-year calibration plots through re-sampling 1000 times, and the calibration curve was visualized to evaluate the consistency between actual and predicted survival rates.

### Statistical methods

All analyses were performed using R software (version 3.5.2). Survival analyses used the Kaplan-Meier (K-M) method and tested the relationship between them using a log-rank test. The spearman rank correlation analyzes the correlation between the two variables. The Venn diagram was drawn using the "VennDiagram" package, and the violin curve was drawn using the "violot" package. Immune-related genes were recruited at IMMPORT (http://www.immport.org/) [74]. According to the lowest AIC value, cox multiple factors determined the genes included in the model [75]. Analysis of variance was used to test the relationship between 3-GRM score and clinical characteristics, and Wilcox test was used to analyze the characteristics of cells in microenvironment in different risk groups. All statistical tests are two-sided tests, P value <0.05 is considered statistically significant.

## Supplementary Material

Supplementary Figures

Supplementary Table 1

Supplementary Table 2A and 2B

Supplementary Table 3A and 3B

Supplementary Tables 4, 5 and 6
