# Supplementary material for: A three-gene signature based on tumour microenvironment predicts overall survival of osteosarcoma in adolescents and young adults
Source: Aging (Albany NY). 2020 Dec 3;13(1):619–45. doi: 10.18632/aging.202170 (PMC7835013; doi:10.18632/aging.202170)
Supplement: Supplementary Figures [file aging-13-202170-s001.pdf]

SUPPLEMENTARY FIGURES

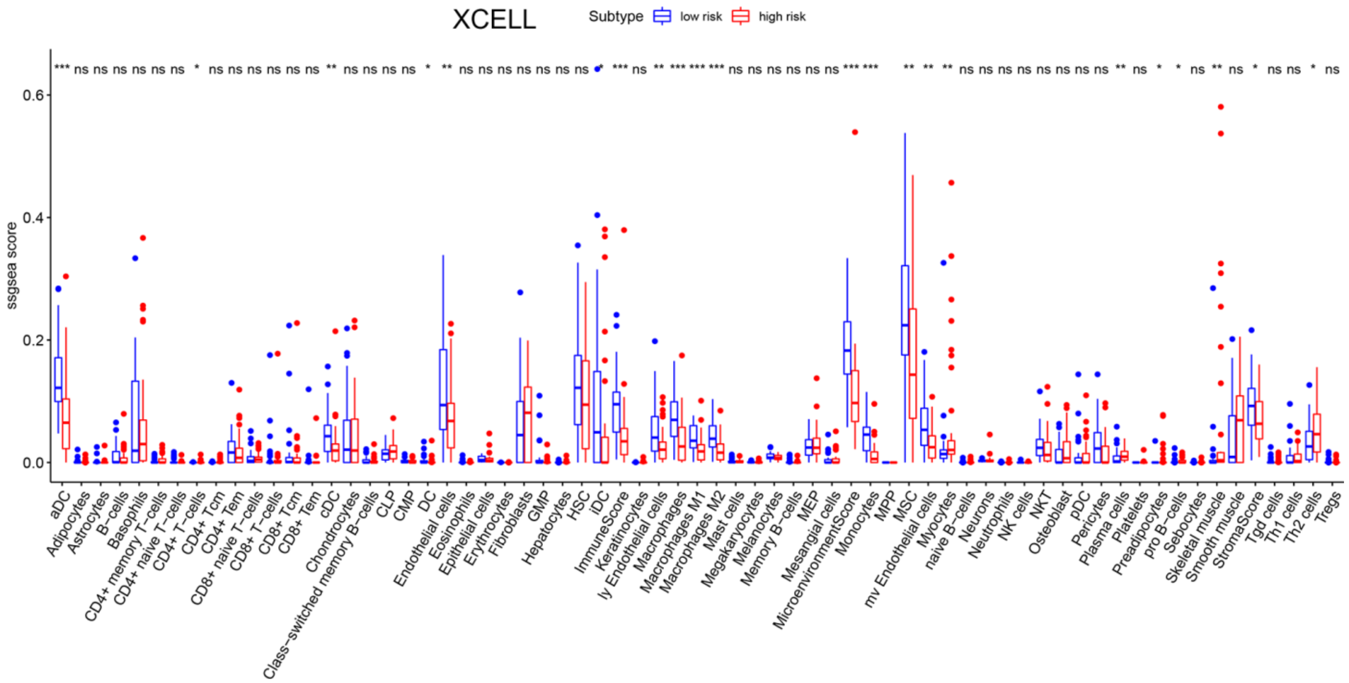

Supplementary Figure 1. Comparison of the level of TIICs between low risk group and high risk group by XCell. The horizontal axis represents the type of microenvironment cells, and the vertical axis represents the relative enrichment score. Blue and red represent low and high risk score, respectively. The Wilcoxon rank-sum test assessed data. *P<0.05, **P<0.01, ***P<0.001. NS, no significance; TIICs, tumour-infiltrating immune cells.

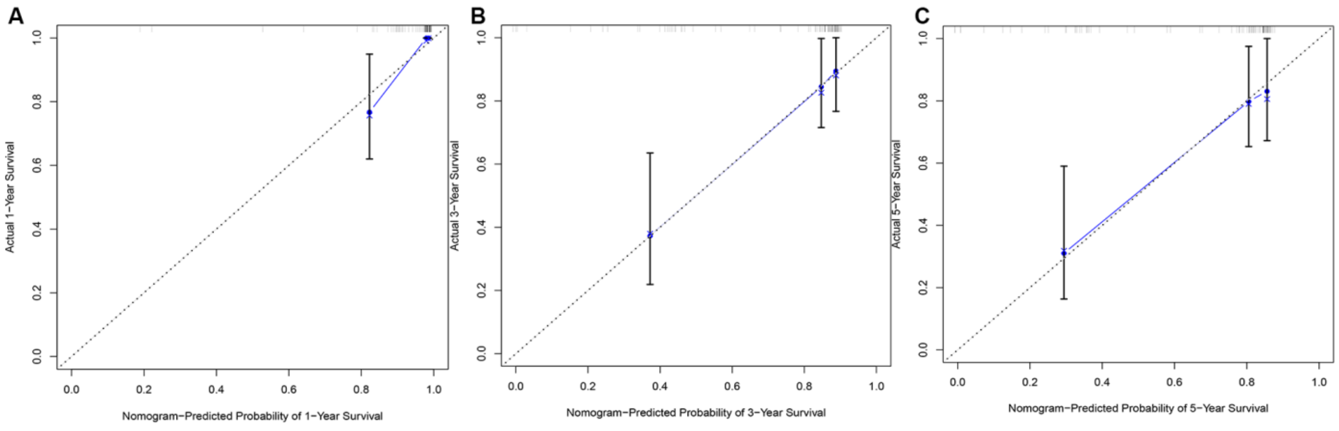

Supplementary Figure 2. Calibration plot of the nomogram model for predicting the probability of OS at 1, 3, and 5 years. (**A**) 1-year calibration curve. (**B**) 3-year calibration curve. (**C**) 5-year calibration curve.

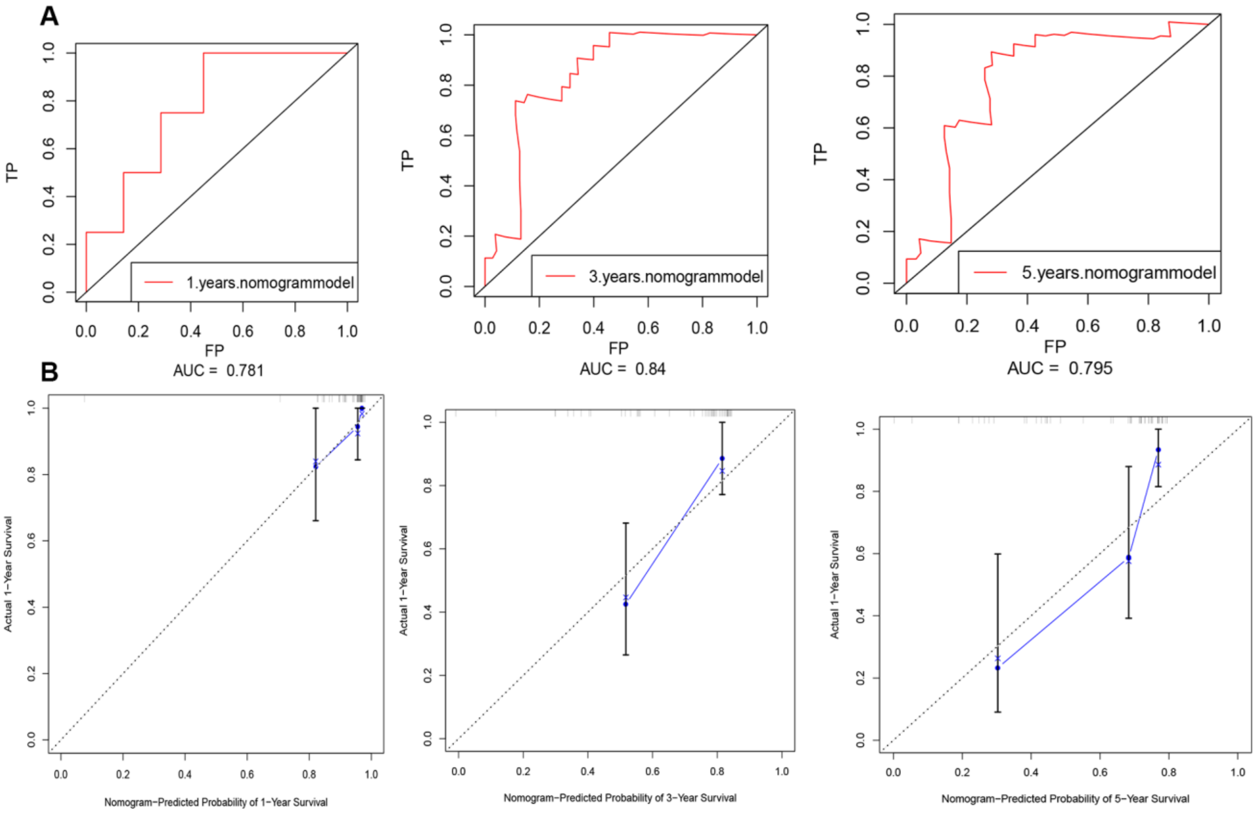

Supplementary Figure 3. (**A**) 1, 3 and 5-year ROC curve for survival prediction of osteosarcoma patients by nomogram model in GSE21257. (**B**) 1, 3 and 5-year calibration plot for survival prediction of osteosarcoma patients by nomogram model in GSE21257.
