# Supplementary material for: A three-gene signature based on tumour microenvironment predicts overall survival of osteosarcoma in adolescents and young adults
Source: Aging (Albany NY). 2020 Dec 3;13(1):619–45. doi: 10.18632/aging.202170 (PMC7835013; doi:10.18632/aging.202170)
Supplement: Supplementary Table 1 [file aging-13-202170-s002.pdf]

SUPPLEMENTARY TABLE

Supplementary Table 1. Clinical baseline data of TARGET 82 cases of adolescent osteosarcoma.

	level	Total
**patient(n)**	-	82
**Survival time (months)**	-	49.88 (±35.01)
**age (years)**	-	14.50 (±3.92)
**Survival status (n[%])**	Alive	56 (68.3)
	Dead	26 (31.7)
**Gender(n[%])**	Male	45 (54.9)
	Female	37 (45.1)
**Race(n[%])**	While	48 (58.5)
	Asian	7 (8.5)
	Black or African American	7 (8.5)
	Unknown	20 (24.4)
**Disease at diagnosis (n[%])**	Non-metastatic	61 (74.4)
	Metastatic	21 (25.6)
**Metastasis at lung (n[%])**	Yes	27 (32.9)
	Unknown	55 (67.1)
**Primary tumor site(n[%])**	Leg/Foot	74 (90.2)
	Arm/hand	6 (7.3)
	Pelvis	2 (2.4)
**Specific tumor site(n[%])**	Femur	44 (53.7)
	Tibia /Fibula	28 (34.1)
	Humerus	6 (7.3)
	Pelvis	4 (4.9)
**Specific tumor side(n[%])**	Left	10 (12.2)
	Right	10 (12.2)
	Unknown	62 (75.6)
**Specific tumor region(n[%])**	Distal	26 (31.7)
	Proximal	22 (26.8)
	Unknown	34 (41.5)
**Definitive Surgery(n[%])**	Limb sparing	38 (46.3)
	Amputation	4 (4.9)
	Unknown	40 (48.8)
**Primary site progression(n[%])**	No	19 (23.2)
	Yes	15 (18.3)
	Unknown	48 (58.5)
