# Supplementary material for: A three-gene signature based on tumour microenvironment predicts overall survival of osteosarcoma in adolescents and young adults
Source: Aging (Albany NY). 2020 Dec 3;13(1):619–45. doi: 10.18632/aging.202170 (PMC7835013; doi:10.18632/aging.202170)
Supplement: Supplementary Table 2A and 2B [file aging-13-202170-s003.doc]

**Supplementary Table 2 A. 262 differential genes up-regulated in the high immune score group.**

| **Gene** | **Low immune score** | **High immune score** | **Log FC** | **P Value** | **FDR** |
| --- | --- | --- | --- | --- | --- |
| **AC011515.2** | 0.116462159 | 0.253279156 | 1.120867084 | 6.99E-05 | 0.002213382 |
| **AC016735.2** | 0.283098655 | 0.887098756 | 1.647789826 | 0.000445246 | 0.008605142 |
| **AC092484.1** | 0.931844345 | 2.017016286 | 1.11406184 | 0.000184646 | 0.004576586 |
| **AC092580.4** | 0.167254426 | 0.565123101 | 1.756520776 | 0.002466786 | 0.027527885 |
| **AC093484.4** | 0.080807234 | 0.196343321 | 1.280822164 | 0.003143742 | 0.03234661 |
| **AF127936.5** | 0.286755389 | 0.645619431 | 1.170863403 | 0.000160026 | 0.004124753 |
| **AGMO** | 0.319282083 | 0.639869195 | 1.002945416 | 0.001876766 | 0.022935219 |
| **ALOX5** | 1.122084351 | 2.350488556 | 1.066779524 | 3.10E-07 | 2.56E-05 |
| **AMICA1** | 0.350104117 | 0.706292434 | 1.012481615 | 1.22E-05 | 0.000554522 |
| **ANKRD22** | 0.198170291 | 0.820861648 | 2.050398389 | 6.64E-06 | 0.000334262 |
| **APOBEC3A** | 0.144059165 | 0.33655558 | 1.22418333 | 2.02E-05 | 0.00081847 |
| **APOC2** | 0.296587482 | 0.607730549 | 1.034974108 | 6.94E-06 | 0.000345216 |
| **APOC4-APOC2** | 0.091731127 | 0.245493927 | 1.420204071 | 3.74E-05 | 0.001368421 |
| **ARHGAP9** | 0.903116376 | 1.83197039 | 1.020412374 | 2.37E-13 | 2.39E-10 |
| **ARL11** | 0.41458497 | 0.996803579 | 1.265641431 | 1.77E-12 | 1.35E-09 |
| **ASCL2** | 0.390812652 | 0.802980344 | 1.038887501 | 8.76E-06 | 0.000417391 |
| **BATF2** | 0.627331783 | 1.280043389 | 1.02889215 | 2.76E-06 | 0.000164559 |
| **C1orf162** | 1.218956941 | 2.623708778 | 1.105960431 | 3.30E-14 | 7.59E-11 |
| **CARD11** | 0.448667932 | 0.976708129 | 1.12227943 | 2.80E-09 | 4.66E-07 |
| **CCL14** | 0.276013994 | 0.55375777 | 1.004513624 | 0.000674659 | 0.011406884 |
| **CCL2** | 1.264199053 | 2.694565121 | 1.091828814 | 4.21E-09 | 6.65E-07 |
| **CCL23** | 0.068655383 | 0.170828332 | 1.315102515 | 8.59E-05 | 0.002613553 |
| **CCL4** | 0.617457627 | 1.419882546 | 1.201359554 | 1.13E-06 | 7.73E-05 |
| **CCL4L1** | 0.666995345 | 1.433808938 | 1.104104193 | 3.53E-06 | 0.000198834 |
| **CCL8** | 0.516612033 | 1.565271162 | 1.599259453 | 8.59E-08 | 8.71E-06 |
| **CCR2** | 0.160547279 | 0.392298705 | 1.28895436 | 6.13E-05 | 0.001991777 |
| **CCR5** | 0.487996527 | 1.216233565 | 1.317477526 | 5.06E-08 | 5.66E-06 |
| **CD2** | 0.568643492 | 1.408924988 | 1.308998453 | 1.59E-05 | 0.000678629 |
| **CD247** | 0.2553752 | 0.569100788 | 1.156065747 | 0.001306599 | 0.017830729 |
| **CD300E** | 0.25828044 | 0.681693641 | 1.400185136 | 4.02E-07 | 3.19E-05 |
| **CD300LF** | 0.42568062 | 0.903837263 | 1.086291626 | 1.30E-06 | 8.55E-05 |
| **CD3D** | 0.445168143 | 1.369165645 | 1.620874737 | 2.47E-06 | 0.000147949 |
| **CD3E** | 0.429901056 | 1.337424638 | 1.63738104 | 4.72E-07 | 3.68E-05 |
| **CD3G** | 0.226665188 | 0.479144264 | 1.079897261 | 0.000461055 | 0.008786301 |
| **CD40LG** | 0.108242989 | 0.245907771 | 1.183843747 | 0.00065491 | 0.011201485 |
| **CD48** | 0.690405317 | 1.568888758 | 1.184227581 | 5.51E-11 | 1.64E-08 |
| **CD5** | 0.208437742 | 0.623045078 | 1.579720013 | 1.72E-05 | 0.000719932 |
| **CD69** | 0.200874657 | 0.471188007 | 1.230007258 | 0.000476688 | 0.008959175 |
| **CD7** | 0.408422759 | 1.07288897 | 1.393365616 | 5.34E-05 | 0.001775933 |
| **CD79A** | 0.323793094 | 0.719425097 | 1.151772274 | 0.000205098 | 0.004938775 |
| **CD8A** | 0.421614623 | 0.952779344 | 1.176217231 | 0.002713673 | 0.029318812 |
| **CEACAM21** | 0.308569395 | 0.647909869 | 1.070198156 | 1.11E-06 | 7.59E-05 |
| **CLEC4E** | 0.183360522 | 0.553669695 | 1.594342502 | 5.32E-07 | 4.04E-05 |
| **CLECL1** | 0.203636322 | 0.531785549 | 1.384849656 | 1.15E-07 | 1.11E-05 |
| **CSF3R** | 0.479258784 | 0.98870656 | 1.04473753 | 8.66E-08 | 8.71E-06 |
| **CTA-384D8.34** | 0.0711014 | 0.15766229 | 1.148887772 | 0.00615943 | 0.049066776 |
| **CTA-384D8.35** | 0.162327526 | 0.368104692 | 1.181208475 | 0.000244426 | 0.005607817 |
| **CTB-41I6.2** | 0.278958448 | 0.663754473 | 1.250599437 | 2.77E-08 | 3.37E-06 |
| **CTB-61M7.2** | 0.091362383 | 0.241370604 | 1.401577805 | 0.001830452 | 0.022587795 |
| **CTD-2562J17.7** | 0.053534632 | 0.157384236 | 1.555746645 | 0.000828829 | 0.013101521 |
| **CTLA4** | 0.149782963 | 0.324137188 | 1.113731016 | 0.000431938 | 0.008416043 |
| **CXCL10** | 1.4107843 | 3.285131205 | 1.219453565 | 3.51E-08 | 4.17E-06 |
| **CXCL11** | 0.52317089 | 1.35693318 | 1.374995505 | 6.99E-05 | 0.002213382 |
| **CXCL9** | 0.912721131 | 2.149517481 | 1.235766806 | 0.000936197 | 0.014220286 |
| **CXCR2P1** | 0.26603342 | 0.990404119 | 1.896409819 | 3.49E-06 | 0.00019881 |
| **CXCR3** | 0.36527579 | 0.906478324 | 1.311286386 | 4.09E-06 | 0.000223207 |
| **CXCR6** | 0.150497039 | 0.472762696 | 1.651381099 | 7.87E-06 | 0.000386211 |
| **CXorf21** | 0.582964281 | 1.381402401 | 1.244654241 | 8.88E-11 | 2.36E-08 |
| **CYP2S1** | 0.498509911 | 1.082750034 | 1.119006127 | 4.84E-08 | 5.48E-06 |
| **DCSTAMP** | 0.908070913 | 1.820281057 | 1.003284354 | 0.000286017 | 0.006260645 |
| **DENND1C** | 0.602051668 | 1.218987105 | 1.017723654 | 2.64E-11 | 1.01E-08 |
| **EBI3** | 0.621340358 | 1.534980243 | 1.304764417 | 8.73E-09 | 1.25E-06 |
| **FASLG** | 0.115186396 | 0.360472452 | 1.645918674 | 5.29E-05 | 0.001769007 |
| **FCGR1A** | 0.744280264 | 1.838033812 | 1.30424542 | 1.89E-11 | 7.85E-09 |
| **FCGR1B** | 0.145195364 | 0.511166234 | 1.815797153 | 3.08E-09 | 5.09E-07 |
| **FCGR1C** | 0.230972281 | 0.922276416 | 1.997479483 | 7.27E-10 | 1.49E-07 |
| **FCGR2B** | 0.346331697 | 0.9635193 | 1.476159133 | 5.38E-08 | 5.96E-06 |
| **FCN1** | 0.243418099 | 0.542337813 | 1.155755319 | 3.98E-05 | 0.001437776 |
| **FGD2** | 0.363793942 | 0.812489908 | 1.159228374 | 2.58E-08 | 3.19E-06 |
| **FPR1** | 0.685777167 | 1.571525106 | 1.196353545 | 2.58E-07 | 2.21E-05 |
| **FUT7** | 0.07730524 | 0.271661578 | 1.813172423 | 1.36E-08 | 1.83E-06 |
| **GAPT** | 0.192423373 | 0.461112404 | 1.260834428 | 0.000146045 | 0.003850843 |
| **GBP1P1** | 0.305158812 | 0.654885827 | 1.101683157 | 0.003388183 | 0.033896393 |
| **GIMAP1** | 0.715251282 | 1.462561469 | 1.031975176 | 1.40E-09 | 2.60E-07 |
| **GIMAP5** | 0.166919249 | 0.367384018 | 1.138138535 | 4.64E-06 | 0.00025048 |
| **GNLY** | 0.37348494 | 0.801178819 | 1.101074212 | 0.000527054 | 0.009640388 |
| **GPBAR1** | 0.165641243 | 0.487952469 | 1.558678693 | 4.01E-07 | 3.19E-05 |
| **GPR174** | 0.091416568 | 0.265824853 | 1.539948436 | 0.00065482 | 0.011201485 |
| **GPR65** | 0.449780495 | 1.150057791 | 1.354413356 | 2.87E-11 | 1.08E-08 |
| **GPR84** | 0.514515936 | 1.104141486 | 1.101637385 | 3.05E-07 | 2.52E-05 |
| **GZMA** | 1.21742879 | 2.643636145 | 1.118686238 | 1.66E-06 | 0.000105502 |
| **GZMH** | 0.326621599 | 0.898378603 | 1.459703369 | 0.000106918 | 0.003065549 |
| **GZMK** | 0.478762347 | 1.23861982 | 1.371351838 | 2.36E-05 | 0.000924781 |
| **HAMP** | 0.104800748 | 0.255917256 | 1.288028407 | 7.54E-05 | 0.002362215 |
| **HCG4P11** | 0.126805025 | 0.278946909 | 1.137378649 | 0.000560153 | 0.010036375 |
| **HCST** | 1.332548164 | 2.769933846 | 1.055663842 | 5.11E-10 | 1.12E-07 |
| **HK3** | 0.736535591 | 1.557262024 | 1.080184566 | 4.75E-10 | 1.06E-07 |
| **HLA-DQB1-AS1** | 0.315312316 | 0.796688193 | 1.337233669 | 0.000222655 | 0.005234166 |
| **HLA-DQB2** | 0.636369774 | 1.339184458 | 1.073417471 | 0.000146529 | 0.003855555 |
| **HLA-DRB9** | 0.152914619 | 0.330323044 | 1.111151284 | 0.002808713 | 0.029860311 |
| **HS3ST4** | 0.089319484 | 0.180725437 | 1.016752754 | 0.002717694 | 0.029337154 |
| **ICAM3** | 0.11671632 | 0.253229208 | 1.11743752 | 2.32E-05 | 0.000917619 |
| **ICOS** | 0.103774443 | 0.339793428 | 1.711206759 | 0.000274634 | 0.006064036 |
| **IDO1** | 0.378683372 | 0.875832057 | 1.209662184 | 0.003459334 | 0.034187318 |
| **IFI30** | 0.68275528 | 1.386947814 | 1.022473033 | 2.80E-09 | 4.66E-07 |
| **IGHA2** | 0.461857115 | 1.014200889 | 1.134824946 | 0.00040521 | 0.008002809 |
| **IGHG2** | 0.797475261 | 1.75257853 | 1.13596742 | 0.000810813 | 0.012921854 |
| **IGHM** | 0.640350938 | 1.684883937 | 1.395714532 | 0.000526744 | 0.009640388 |
| **IGHV1-24** | 0.197351903 | 0.432738013 | 1.132723429 | 0.005728744 | 0.046876602 |
| **IGKC** | 1.122946677 | 2.532492227 | 1.173268418 | 0.000553984 | 0.010010031 |
| **IGKV3-11** | 0.557752368 | 1.216221287 | 1.124709107 | 0.004487656 | 0.040309513 |
| **IGLC2** | 1.014661554 | 2.201993205 | 1.117811428 | 0.003554536 | 0.034885105 |
| **IGLL5** | 0.2457319 | 0.566450863 | 1.204865658 | 0.00601466 | 0.048175922 |
| **IGSF21** | 1.225320333 | 2.511504157 | 1.035392704 | 5.97E-11 | 1.73E-08 |
| **IKZF1** | 0.326401438 | 0.881934203 | 1.434023612 | 5.30E-09 | 8.12E-07 |
| **IKZF3** | 0.430361101 | 0.892850099 | 1.052870299 | 6.85E-05 | 0.0021783 |
| **IL10** | 0.247637041 | 0.561638197 | 1.181413932 | 3.81E-05 | 0.001389766 |
| **IL10RA** | 1.068744753 | 2.163459303 | 1.017422646 | 1.47E-11 | 7.01E-09 |
| **IL12RB1** | 0.430881709 | 1.042589935 | 1.274808076 | 6.85E-10 | 1.42E-07 |
| **IL1B** | 0.365512061 | 0.764880868 | 1.06531605 | 1.29E-06 | 8.52E-05 |
| **IL27** | 0.084926173 | 0.251045854 | 1.563669749 | 9.73E-06 | 0.000457905 |
| **IL2RA** | 0.285318139 | 0.777091184 | 1.445512423 | 1.51E-06 | 9.65E-05 |
| **ITGAL** | 0.58148707 | 1.248304899 | 1.102151339 | 4.76E-08 | 5.42E-06 |
| **ITGAM** | 1.003074344 | 2.044352524 | 1.027215456 | 1.35E-11 | 6.70E-09 |
| **ITGB2-AS1** | 0.422134888 | 0.846617427 | 1.004006118 | 0.000215269 | 0.00509147 |
| **KLRC1** | 0.070339368 | 0.148568071 | 1.078719814 | 0.000599915 | 0.010554436 |
| **LAG3** | 0.535989169 | 1.33853472 | 1.320378809 | 2.62E-09 | 4.41E-07 |
| **LAIR2** | 0.14251805 | 0.302525346 | 1.085911368 | 0.003875598 | 0.036843705 |
| **LCK** | 0.380468058 | 0.886745645 | 1.220745003 | 1.29E-06 | 8.52E-05 |
| **LILRA1** | 0.126588409 | 0.451870459 | 1.83576393 | 5.87E-09 | 8.80E-07 |
| **LILRA2** | 0.167085924 | 0.43494458 | 1.380241387 | 2.75E-07 | 2.31E-05 |
| **LILRA5** | 0.291903729 | 0.675543541 | 1.210556121 | 5.84E-07 | 4.37E-05 |
| **LILRA6** | 0.356941163 | 0.767576625 | 1.104624492 | 1.46E-07 | 1.35E-05 |
| **LILRB1** | 0.706528004 | 1.552236697 | 1.135529917 | 6.22E-12 | 3.49E-09 |
| **LILRB2** | 0.675437593 | 1.746060232 | 1.370208944 | 1.58E-13 | 2.11E-10 |
| **LILRB4** | 1.008219578 | 2.255949826 | 1.161925107 | 2.55E-12 | 1.74E-09 |
| **LILRB5** | 0.499195773 | 1.165145843 | 1.222832929 | 2.72E-07 | 2.29E-05 |
| **LINC00996** | 0.137393462 | 0.302429068 | 1.138283456 | 0.000150484 | 0.003955495 |
| **LINC01272** | 0.438288399 | 0.98012737 | 1.161088752 | 6.12E-06 | 0.000314092 |
| **LLNLR-470E3.1** | 0.276843099 | 0.66210572 | 1.257993033 | 2.93E-05 | 0.00111128 |
| **LRRC25** | 1.278948802 | 2.611774746 | 1.030071964 | 4.78E-16 | 4.88E-12 |
| **LST1** | 1.010937324 | 2.266685783 | 1.164890857 | 1.43E-13 | 2.01E-10 |
| **LTB** | 0.637453515 | 1.481515484 | 1.216681659 | 3.71E-06 | 0.000206957 |
| **LY6H** | 0.558031762 | 1.15144936 | 1.045031819 | 0.001001512 | 0.014881293 |
| **LY86** | 1.276301155 | 2.559497664 | 1.003891902 | 7.62E-13 | 6.64E-10 |
| **MAGEB2** | 0.372689021 | 1.53619982 | 2.043321659 | 0.00260711 | 0.028644909 |
| **MS4A1** | 0.061655567 | 0.14933954 | 1.276293132 | 0.004190809 | 0.038610366 |
| **MS4A14** | 0.382042089 | 0.809604963 | 1.083486549 | 1.85E-06 | 0.000116489 |
| **MS4A4A** | 1.390355693 | 2.890239135 | 1.055734851 | 9.21E-13 | 7.50E-10 |
| **MT-TA** | 0.620838632 | 1.348168078 | 1.118710133 | 0.002419467 | 0.027223044 |
| **MYO1G** | 0.339444744 | 0.736526453 | 1.117560594 | 8.51E-07 | 6.14E-05 |
| **NAPSB** | 0.32243944 | 0.917034991 | 1.50794856 | 2.37E-06 | 0.000143023 |
| **NAT8B** | 0.053118569 | 0.18333975 | 1.787231416 | 2.04E-06 | 0.000126362 |
| **NCF1** | 0.369776624 | 1.103500574 | 1.577361451 | 3.70E-11 | 1.30E-08 |
| **NCF1B** | 0.278522916 | 0.926466594 | 1.733942924 | 2.13E-10 | 5.27E-08 |
| **NCF1C** | 0.56023706 | 1.65686801 | 1.564349353 | 2.12E-12 | 1.53E-09 |
| **NCF4** | 1.415476041 | 2.886131916 | 1.027849913 | 1.48E-15 | 5.35E-12 |
| **NEURL3** | 0.412724192 | 0.846812639 | 1.036864797 | 0.000687822 | 0.011491003 |
| **NKG7** | 0.951347789 | 2.089432211 | 1.135066196 | 5.56E-07 | 4.18E-05 |
| **NLRP3** | 0.395421885 | 0.829856233 | 1.069468701 | 3.51E-08 | 4.17E-06 |
| **NTSR1** | 0.35339307 | 0.7386142 | 1.06354725 | 0.001382414 | 0.018653728 |
| **OSM** | 0.488386247 | 1.051312317 | 1.106096842 | 1.66E-05 | 0.000700329 |
| **P2RY10** | 0.104954332 | 0.283621299 | 1.434204157 | 0.001041346 | 0.015304049 |
| **P2RY13** | 0.401489734 | 0.909662024 | 1.179967527 | 3.15E-06 | 0.000184069 |
| **P2RY6** | 0.465146489 | 1.22185454 | 1.393315503 | 4.75E-10 | 1.06E-07 |
| **PCED1B** | 0.606237694 | 1.470571447 | 1.278421416 | 8.56E-14 | 1.44E-10 |
| **PCED1B-AS1** | 0.446544615 | 1.264211655 | 1.50136179 | 1.94E-12 | 1.44E-09 |
| **PDE1B** | 0.302265085 | 0.752018355 | 1.314953534 | 1.93E-10 | 4.87E-08 |
| **PF4** | 0.543425552 | 1.169694157 | 1.105977044 | 0.000989303 | 0.01481315 |
| **PIK3R5** | 0.575240958 | 1.185827711 | 1.04365611 | 5.50E-10 | 1.20E-07 |
| **PLD4** | 0.31397642 | 0.646733461 | 1.042515039 | 0.000434462 | 0.008416043 |
| **PPBP** | 0.341504885 | 0.734298753 | 1.104460933 | 0.000600009 | 0.010554436 |
| **PRKCB** | 0.387123815 | 0.813122755 | 1.070678106 | 1.29E-05 | 0.000579256 |
| **PSTPIP1** | 0.919285933 | 1.876926048 | 1.029786239 | 2.58E-07 | 2.21E-05 |
| **PYHIN1** | 0.141597442 | 0.285724765 | 1.012830888 | 0.000741817 | 0.012136197 |
| **RASGRP4** | 0.540000816 | 1.097991482 | 1.02383337 | 4.51E-09 | 7.07E-07 |
| **RBAKDN** | 0.452657843 | 1.015522986 | 1.165730038 | 0.000295689 | 0.006400251 |
| **RETN** | 0.081473931 | 0.389065804 | 2.255603765 | 0.000308072 | 0.006617271 |
| **RGS18** | 0.372031066 | 0.849327822 | 1.190898412 | 2.14E-08 | 2.70E-06 |
| **RP11-1094M14.5** | 0.125454285 | 0.327122869 | 1.382670873 | 0.000723115 | 0.011922896 |
| **RP11-1094M14.8** | 0.194957873 | 0.528597635 | 1.439007555 | 0.000261588 | 0.005894476 |
| **RP11-121A8.1** | 0.2039874 | 0.477975372 | 1.228456246 | 8.35E-07 | 6.08E-05 |
| **RP11-130L8.2** | 0.250203741 | 0.51451596 | 1.04011247 | 0.000614134 | 0.010772938 |
| **RP11-219E7.1** | 0.708715964 | 1.539266286 | 1.118963381 | 1.40E-07 | 1.31E-05 |
| **RP11-24F11.2** | 0.225600879 | 0.567270035 | 1.330262969 | 3.52E-06 | 0.000198834 |
| **RP11-344B5.2** | 0.675694814 | 1.54122515 | 1.189633945 | 1.93E-08 | 2.51E-06 |
| **RP11-368J21.3** | 0.139347233 | 0.32081788 | 1.203070194 | 7.98E-06 | 0.000386748 |
| **RP11-405M12.4** | 0.189562446 | 0.505428419 | 1.414833601 | 2.09E-07 | 1.86E-05 |
| **RP11-416L21.2** | 0.226920242 | 0.476129506 | 1.069168726 | 0.001227082 | 0.017106007 |
| **RP11-455F5.5** | 0.100510773 | 0.215298503 | 1.09898815 | 0.003535519 | 0.03483121 |
| **RP11-472N13.3** | 0.363654911 | 0.757403109 | 1.058491284 | 2.17E-05 | 0.000868899 |
| **RP11-494O16.3** | 0.279067269 | 0.624623368 | 1.162373618 | 0.000326047 | 0.00682914 |
| **RP11-514O12.4** | 0.14653318 | 0.331354633 | 1.17714872 | 3.57E-05 | 0.001316429 |
| **RP11-61A14.1** | 0.100911989 | 0.209195028 | 1.051750979 | 0.001626249 | 0.020825814 |
| **RP11-626A5.2** | 0.699206363 | 1.442882943 | 1.045164044 | 0.000128818 | 0.003513975 |
| **RP11-652L8.4** | 0.102987291 | 0.298449072 | 1.535018455 | 1.96E-05 | 0.000801059 |
| **RP11-702B10.2** | 0.809392795 | 1.84438604 | 1.188228739 | 5.43E-05 | 0.001799356 |
| **RP11-733O18.1** | 0.401209062 | 0.951755837 | 1.24623732 | 2.03E-06 | 0.00012617 |
| **RP11-807H22.7** | 0.488720459 | 1.080481276 | 1.144592666 | 0.000253653 | 0.005756769 |
| **RP11-84C10.2** | 0.092175151 | 0.199498554 | 1.113928509 | 0.001601684 | 0.020631585 |
| **RP11-865I6.2** | 1.010555285 | 2.181500255 | 1.110172392 | 0.004801777 | 0.041854002 |
| **RP11-8L8.2** | 0.111853005 | 0.347600653 | 1.635826773 | 7.15E-08 | 7.62E-06 |
| **RP13-1016M1.2** | 0.187683758 | 0.446208356 | 1.249413728 | 0.004340528 | 0.039567574 |
| **RP3-449M8.9** | 0.175911763 | 0.389375231 | 1.146309159 | 0.000251554 | 0.005719395 |
| **RP5-1091N2.9** | 0.270172114 | 0.616050618 | 1.189170124 | 5.07E-07 | 3.90E-05 |
| **RP5-1171I10.5** | 0.418999093 | 0.973113859 | 1.215661497 | 2.58E-08 | 3.19E-06 |
| **RP5-899E9.1** | 0.837068093 | 1.939952309 | 1.212604295 | 3.82E-06 | 0.00021237 |
| **RPL7AP28** | 0.281786031 | 0.649618348 | 1.204992286 | 0.002339569 | 0.02654875 |
| **RTP4** | 0.766478959 | 1.636667168 | 1.094442873 | 2.14E-08 | 2.70E-06 |
| **RUFY4** | 0.914549405 | 1.962668996 | 1.101683869 | 9.87E-05 | 0.002875085 |
| **S100A8** | 0.508391707 | 1.225546572 | 1.269412908 | 3.36E-06 | 0.000192318 |
| **S100A9** | 1.651241405 | 3.43191475 | 1.055462664 | 5.92E-10 | 1.27E-07 |
| **S1PR4** | 0.331887229 | 0.680937287 | 1.036828817 | 0.000236132 | 0.005457176 |
| **SASH3** | 1.138818878 | 2.33506533 | 1.035924601 | 2.06E-11 | 8.26E-09 |
| **SCIMP** | 0.3890938 | 0.812012892 | 1.061384638 | 2.44E-07 | 2.12E-05 |
| **SELL** | 1.104793053 | 2.221997422 | 1.008080989 | 2.06E-07 | 1.84E-05 |
| **SH2D1A** | 0.165634825 | 0.381300685 | 1.202923092 | 0.001919567 | 0.023343598 |
| **SIGLEC1** | 0.972871062 | 2.149918088 | 1.143961177 | 7.18E-09 | 1.05E-06 |
| **SIGLEC10** | 0.516767275 | 1.047788999 | 1.019761601 | 8.97E-07 | 6.46E-05 |
| **SIGLEC11** | 0.177074276 | 0.494746655 | 1.482335305 | 1.16E-06 | 7.87E-05 |
| **SIGLEC12** | 0.29017095 | 0.658725404 | 1.182774095 | 0.000642295 | 0.011026187 |
| **SIGLEC14** | 0.537500589 | 1.162391804 | 1.112758195 | 2.61E-05 | 0.001007476 |
| **SIGLEC16** | 0.200048571 | 0.502112747 | 1.327661024 | 7.24E-06 | 0.000359357 |
| **SIGLEC7** | 0.567099329 | 1.31754327 | 1.21617699 | 5.51E-11 | 1.64E-08 |
| **SIGLEC8** | 0.10960621 | 0.233196657 | 1.089217567 | 0.005752736 | 0.04702722 |
| **SIRPB2** | 0.332529647 | 0.723592762 | 1.121695006 | 1.30E-07 | 1.23E-05 |
| **SIRPG** | 0.270781095 | 0.686487166 | 1.342105733 | 0.000130966 | 0.003540688 |
| **SIT1** | 0.259144513 | 0.721498127 | 1.477238805 | 3.15E-06 | 0.000184069 |
| **SLA2** | 0.28042103 | 0.588060226 | 1.068369367 | 0.000101456 | 0.002945721 |
| **SLAMF1** | 0.155158683 | 0.313552725 | 1.014963627 | 2.77E-05 | 0.001059861 |
| **SLAMF8** | 1.195021683 | 2.424004739 | 1.020355724 | 1.12E-10 | 2.93E-08 |
| **SLC22A18AS** | 0.417296493 | 0.873218259 | 1.065269502 | 1.89E-11 | 7.85E-09 |
| **SNX20** | 0.34581284 | 0.990605544 | 1.518319258 | 1.02E-15 | 4.88E-12 |
| **SOWAHD** | 0.324746867 | 0.724200653 | 1.15707387 | 4.99E-07 | 3.84E-05 |
| **SP140** | 0.174891591 | 0.430664861 | 1.300104687 | 1.83E-05 | 0.000751091 |
| **SUCNR1** | 0.814145507 | 1.66164799 | 1.029256223 | 5.27E-07 | 4.01E-05 |
| **THEMIS** | 0.072865628 | 0.150326504 | 1.044789065 | 0.006176053 | 0.049066776 |
| **TIGIT** | 0.09732511 | 0.331403825 | 1.767706281 | 0.001218172 | 0.017062197 |
| **TLR7** | 0.451098685 | 1.111638899 | 1.30117324 | 8.16E-08 | 8.32E-06 |
| **TLR8** | 0.205088421 | 0.601919744 | 1.553325095 | 1.40E-09 | 2.60E-07 |
| **TMEM150B** | 0.623532781 | 1.345410501 | 1.109509109 | 5.63E-08 | 6.21E-06 |
| **TMEM176A** | 1.210032762 | 2.432331587 | 1.007293808 | 7.91E-10 | 1.59E-07 |
| **TNF** | 0.182475175 | 0.465224876 | 1.350228039 | 1.24E-05 | 0.00056487 |
| **TNFSF13B** | 0.961050027 | 1.956526881 | 1.025611495 | 5.71E-08 | 6.24E-06 |
| **TNFSF14** | 0.110597724 | 0.227709749 | 1.04187436 | 0.002119162 | 0.024932483 |
| **TNFSF8** | 0.450255026 | 0.920250102 | 1.031283626 | 3.20E-06 | 0.000184342 |
| **TOMM20P2** | 0.097570738 | 0.333140983 | 1.771612406 | 0.001163108 | 0.01654396 |
| **TRAV1-2** | 0.051332285 | 0.184060998 | 1.842245573 | 0.000263817 | 0.005928831 |
| **TRAV12-3** | 0.062838099 | 0.184982361 | 1.557676257 | 0.003786852 | 0.036315825 |
| **TRAV19** | 0.092022394 | 0.342286116 | 1.895145887 | 0.001482042 | 0.01967146 |
| **TRAV2** | 0.067991999 | 0.281959306 | 2.05205007 | 0.000779354 | 0.012587272 |
| **TRAV4** | 0.10482775 | 0.252750187 | 1.269691478 | 0.001498025 | 0.01975985 |
| **TRAV8-6** | 0.051426073 | 0.22038594 | 2.099460282 | 0.000976009 | 0.014631446 |
| **TRBC2** | 0.594058879 | 1.688976113 | 1.50747109 | 2.26E-06 | 0.000137052 |
| **TRBV20-1** | 0.147595847 | 0.602003144 | 2.02811889 | 3.84E-05 | 0.001396994 |
| **TRBV25-1** | 0.035644779 | 0.239448487 | 2.747952626 | 3.20E-06 | 0.000184342 |
| **TRBV28** | 0.311323377 | 1.103193074 | 1.825199487 | 4.74E-06 | 0.000254776 |
| **TRBV29-1** | 0.075751457 | 0.245449181 | 1.696078817 | 0.006235241 | 0.049358252 |
| **TRBV30** | 0.034968971 | 0.224668792 | 2.683652498 | 0.003606594 | 0.035229144 |
| **TRBV6-5** | 0.089963852 | 0.328420835 | 1.868128315 | 7.69E-05 | 0.002405979 |
| **TRBV7-3** | 0.091367611 | 0.225357073 | 1.302457997 | 0.000413508 | 0.008153956 |
| **TREML1** | 0.226089621 | 0.483479431 | 1.096559753 | 5.16E-06 | 0.000274063 |
| **TRGC2** | 0.100999398 | 0.396560573 | 1.973194552 | 4.92E-05 | 0.001671729 |
| **TRGV2** | 0.073032407 | 0.170946997 | 1.226940389 | 0.004928489 | 0.042344774 |
| **TRPC2** | 0.123575239 | 0.316804929 | 1.358205085 | 8.17E-08 | 8.32E-06 |
| **TRPM2** | 0.649945044 | 1.320172407 | 1.022336708 | 1.61E-09 | 2.95E-07 |
| **VENTX** | 0.251472898 | 0.594526145 | 1.241337332 | 7.87E-06 | 0.000386211 |
| **VSIG4** | 1.68451873 | 3.643694439 | 1.113065509 | 4.69E-11 | 1.48E-08 |
| **XCL2** | 0.318417543 | 0.727871047 | 1.192763056 | 0.002728301 | 0.029413954 |
| **ZAP70** | 0.221859034 | 0.513222138 | 1.209940101 | 3.31E-05 | 0.001236975 |
| **ZBP1** | 0.110605882 | 0.231213028 | 1.063794582 | 0.00288998 | 0.030330913 |
| **ZNF683** | 0.190749697 | 0.529139123 | 1.471966325 | 0.002177464 | 0.025397429 |

**Supplementary Table2 B. 320 differential genes down-regulated in the high immune score group.**

| **Gene** | **Low immune score** | **High immune score** | **Log FC** | **P Value** | **FDR** |
| --- | --- | --- | --- | --- | --- |
| **AC003664.1** | 0.361871272 | 0.131155852 | -1.464194402 | 0.00110228 | 0.01598369 |
| **AC003988.1** | 0.470024478 | 0.215496691 | -1.125070177 | 0.005236042 | 0.044043433 |
| **AC004014.4** | 0.195540679 | 0.086033414 | -1.184499781 | 0.000462282 | 0.008786301 |
| **AC005086.3** | 0.312009968 | 0.130121986 | -1.261727378 | 0.003709757 | 0.035931154 |
| **AC005514.2** | 0.405186146 | 0.145647165 | -1.47610723 | 0.001013054 | 0.015043946 |
| **AC005783.1** | 0.420764866 | 0.155721081 | -1.434049976 | 0.003701883 | 0.035868649 |
| **AC007279.1** | 0.323089971 | 0.079618444 | -2.020761392 | 0.004543596 | 0.040540885 |
| **AC012627.1** | 0.698467339 | 0.266268841 | -1.391309041 | 0.00343106 | 0.034187318 |
| **AC013470.6** | 0.483894686 | 0.21754996 | -1.153346341 | 0.003591713 | 0.035097356 |
| **AC016142.1** | 0.192557747 | 0.064583536 | -1.576052818 | 0.001954654 | 0.023624186 |
| **AC019178.2** | 0.199315211 | 0.09797528 | -1.024562122 | 0.001332154 | 0.018141176 |
| **AC037445.1** | 0.18896973 | 0.050733245 | -1.897151794 | 0.003188092 | 0.032603722 |
| **AC063976.7** | 0.264616203 | 0.129994547 | -1.0254503 | 0.005472216 | 0.045434799 |
| **AC064853.3** | 0.19716889 | 0.011638184 | -4.082494036 | 0.002786994 | 0.029798473 |
| **AC068538.2** | 0.191285567 | 0.088101672 | -1.11848672 | 0.004945775 | 0.042464402 |
| **AC093642.4** | 0.182179532 | 0.039314237 | -2.212237129 | 0.000718724 | 0.011873751 |
| **AC104532.3** | 0.339501091 | 0.128707203 | -1.399323414 | 0.001676287 | 0.021245867 |
| **AC104981.1** | 0.644717973 | 0.198676921 | -1.69824391 | 0.000350425 | 0.007237717 |
| **AC114803.3** | 0.31817685 | 0.112551867 | -1.499238883 | 0.001493945 | 0.019716328 |
| **AC116050.1** | 0.197505352 | 0.032389789 | -2.608280769 | 0.006231643 | 0.049345233 |
| **AC139099.4** | 0.250288709 | 0.039042581 | -2.680472869 | 0.001810477 | 0.022406987 |
| **ADAMTS9-AS2** | 0.430265666 | 0.168282892 | -1.354339203 | 6.73E-05 | 0.002155932 |
| **AF064858.8** | 0.370021082 | 0.110646867 | -1.741644868 | 0.001549451 | 0.020216497 |
| **AF196972.4** | 0.357541411 | 0.153345662 | -1.221322993 | 0.000174065 | 0.004392499 |
| **AIRE** | 0.164184295 | 0.037445691 | -2.132444505 | 0.000265208 | 0.005950788 |
| **AKNAD1** | 0.482689676 | 0.212196601 | -1.185694424 | 7.97E-05 | 0.002470906 |
| **AL391001.1** | 0.359722649 | 0.087731724 | -2.03571448 | 0.004584854 | 0.040836883 |
| **ANGPTL7** | 0.585512638 | 0.104591105 | -2.484940145 | 0.000128345 | 0.003504869 |
| **ANP32BP3** | 0.378464469 | 0.144965268 | -1.384450578 | 0.002665309 | 0.028994706 |
| **AP000473.8** | 0.374688241 | 0.10865942 | -1.785877448 | 0.000459358 | 0.008786301 |
| **AP000962.1** | 0.266722797 | 0.054678564 | -2.286293887 | 0.001830349 | 0.022587795 |
| **AP000962.2** | 0.445588641 | 0.092381268 | -2.270040204 | 0.000644132 | 0.011046016 |
| **AP001065.7** | 0.292857079 | 0.123312343 | -1.247879561 | 0.000835739 | 0.013177757 |
| **AP001172.2** | 0.197596756 | 0.049439782 | -1.998814981 | 0.000885467 | 0.013713603 |
| **AP001429.1** | 0.537672721 | 0.261439301 | -1.040252247 | 0.000212794 | 0.005061363 |
| **ARF1P2** | 0.412459945 | 0.181603352 | -1.183463192 | 0.001138309 | 0.016346983 |
| **ARHGEF4** | 0.677883147 | 0.193689625 | -1.807289925 | 0.001201571 | 0.016890194 |
| **ASS1P9** | 0.210547364 | 0.089811628 | -1.229170667 | 0.000309439 | 0.006635345 |
| **BCL11B** | 1.477627665 | 0.711707609 | -1.053926218 | 0.002324722 | 0.02639212 |
| **BEGAIN** | 0.952027184 | 0.459804295 | -1.049982825 | 0.001640672 | 0.020825814 |
| **BNIP3P37** | 0.209941787 | 0.065670618 | -1.676669407 | 0.006095004 | 0.048736877 |
| **BSPRY** | 0.201226758 | 0.038155784 | -2.398848488 | 0.000851074 | 0.013286847 |
| **BTBD17** | 0.224304954 | 0.078720936 | -1.5106422 | 0.006080692 | 0.048637838 |
| **BTBD18** | 0.184016934 | 0.089510897 | -1.039703307 | 0.003020004 | 0.031360544 |
| **C8orf59P2** | 0.187078765 | 0.071297016 | -1.391732215 | 0.006221377 | 0.049294852 |
| **C9orf135-AS1** | 0.355063555 | 0.095763194 | -1.890534113 | 0.000757949 | 0.012337888 |
| **CACNA1B** | 0.63603023 | 0.161937072 | -1.97366204 | 0.000591887 | 0.010491983 |
| **CACNA1G** | 1.195299633 | 0.523538887 | -1.191003706 | 0.000737101 | 0.012066866 |
| **CACNG4** | 1.337318477 | 0.461916841 | -1.533638028 | 0.004632815 | 0.041022561 |
| **CAMK2A** | 0.509681731 | 0.248773693 | -1.034762707 | 0.004899769 | 0.042333174 |
| **CAPN14** | 0.336868212 | 0.1624492 | -1.05219566 | 0.003912471 | 0.036905753 |
| **CCDC26** | 0.391790832 | 0.061175114 | -2.679066853 | 0.000968513 | 0.014536329 |
| **CCDC27** | 0.214216571 | 0.056279855 | -1.928379558 | 0.001127374 | 0.016241461 |
| **CCKAR** | 1.182290788 | 0.570781977 | -1.050573228 | 0.005711882 | 0.046753774 |
| **CDH23** | 1.538974814 | 0.593490015 | -1.37467396 | 0.000115737 | 0.003244758 |
| **CEP170P1** | 0.234082609 | 0.110725739 | -1.080027131 | 0.000171673 | 0.004336459 |
| **CHRND** | 0.413750249 | 0.179752132 | -1.202751299 | 0.005011866 | 0.042954034 |
| **CLCNKB** | 0.742038645 | 0.328764728 | -1.174438799 | 0.001588601 | 0.020473702 |
| **CLDN18** | 0.342498165 | 0.154517185 | -1.148328952 | 0.003490176 | 0.034438377 |
| **CMB9-55A18.1** | 0.187825445 | 0.090183478 | -1.058457466 | 0.002224569 | 0.025823816 |
| **COCH** | 1.897545929 | 0.858849052 | -1.143658309 | 0.002116307 | 0.024910487 |
| **COL9A1** | 0.9857729 | 0.265599462 | -1.892003096 | 0.002844553 | 0.030203203 |
| **COX6CP14** | 0.321156533 | 0.116474074 | -1.463267782 | 0.000928995 | 0.014220286 |
| **CRYZP1** | 0.353419643 | 0.139390899 | -1.342245862 | 0.000822584 | 0.013010945 |
| **CTB-187L3.1** | 0.810088578 | 0.403923054 | -1.003999175 | 0.00256677 | 0.028263558 |
| **CTC-1337H24.3** | 0.307276065 | 0.100929702 | -1.606184596 | 0.000109631 | 0.003132669 |
| **CTC-215O4.4** | 0.484326021 | 0.226446333 | -1.096809335 | 0.000894502 | 0.01381964 |
| **CTC-239J10.1** | 0.265765784 | 0.067735351 | -1.972174506 | 0.001499968 | 0.019775151 |
| **CTD-2006M22.2** | 0.486977448 | 0.23374383 | -1.058926677 | 0.002243514 | 0.025871662 |
| **CTD-2062F14.3** | 0.19256557 | 0.065437405 | -1.557162336 | 0.001941393 | 0.023486392 |
| **CTD-2265O21.7** | 0.166373037 | 0.06915744 | -1.266465276 | 0.002644551 | 0.02881372 |
| **CTD-2313N18.7** | 0.546856661 | 0.25530022 | -1.098967949 | 0.000265265 | 0.005950788 |
| **CTD-2540M10.1** | 0.329171961 | 0.05849493 | -2.492457965 | 0.005007731 | 0.042937979 |
| **CTD-2562G15.2** | 0.361230598 | 0.126341758 | -1.515588554 | 0.004924694 | 0.042333174 |
| **CTD-3051D23.3** | 0.173505654 | 0.037208923 | -2.221262135 | 0.004217531 | 0.038838805 |
| **CTD-3252C9.2** | 0.382734449 | 0.174017716 | -1.137109569 | 0.002857557 | 0.030303062 |
| **CYP24A1** | 0.815387998 | 0.303753386 | -1.424586231 | 0.004099194 | 0.038138361 |
| **CYP3A5** | 0.207040239 | 0.0991103 | -1.062804281 | 0.000317208 | 0.006722506 |
| **CYP51A1P1** | 0.24271672 | 0.112585765 | -1.10824907 | 0.000409518 | 0.008081574 |
| **DBNDD1** | 0.89223981 | 0.303486798 | -1.555797759 | 0.005685186 | 0.046610778 |
| **DCUN1D2-AS** | 0.713528392 | 0.310269952 | -1.201446851 | 0.001158361 | 0.016521856 |
| **DDTP1** | 0.156149929 | 0.053841907 | -1.536130497 | 0.002925374 | 0.030649085 |
| **DLEU2L** | 0.250728171 | 0.114663029 | -1.128723807 | 0.001076228 | 0.015641841 |
| **DNER** | 0.281409941 | 0.091648954 | -1.618482967 | 0.004949953 | 0.042485837 |
| **EDN2** | 0.400587123 | 0.066647673 | -2.587489636 | 0.000210498 | 0.005016195 |
| **EDRF1-AS1** | 0.203809484 | 0.097761488 | -1.05988303 | 0.003389639 | 0.033896393 |
| **EEF1A2** | 2.26076983 | 1.059112067 | -1.093958866 | 0.004925463 | 0.042333174 |
| **EIF1AX-AS1** | 0.428015938 | 0.142020904 | -1.591561221 | 0.002920573 | 0.030611484 |
| **EIF4BP2** | 0.210609915 | 0.102907204 | -1.033229376 | 0.001903761 | 0.023231401 |
| **EIF4BP9** | 0.198057578 | 0.084005249 | -1.237368516 | 0.004957654 | 0.042537476 |
| **EIF5A2P1** | 0.350699897 | 0.120857287 | -1.536932543 | 0.003121594 | 0.032131811 |
| **EMILIN3** | 1.075978343 | 0.526439339 | -1.031309833 | 0.005782368 | 0.047177847 |
| **ENO1-IT1** | 0.889797108 | 0.419236764 | -1.085711173 | 0.00129912 | 0.017830729 |
| **ERBB3** | 0.494357138 | 0.142790633 | -1.791652317 | 0.003802711 | 0.036359183 |
| **ESPN** | 1.027771828 | 0.32492544 | -1.661339403 | 0.000174767 | 0.004405793 |
| **ETF1P2** | 0.440140384 | 0.189540419 | -1.21545822 | 6.93E-06 | 0.000345163 |
| **FAM196A** | 0.536159965 | 0.236957823 | -1.178033208 | 0.000874802 | 0.013556989 |
| **FAM60BP** | 0.276226439 | 0.106257282 | -1.378289699 | 0.00554547 | 0.04580725 |
| **FBXO40** | 0.192274763 | 0.076235997 | -1.334625129 | 0.004535426 | 0.04048228 |
| **FGF11** | 0.329103888 | 0.143804077 | -1.194438495 | 0.000947609 | 0.014344454 |
| **FLG** | 0.529195162 | 0.221605129 | -1.255808603 | 0.000664335 | 0.011239849 |
| **FLRT1** | 0.86349477 | 0.428462091 | -1.011019875 | 0.004134464 | 0.038171257 |
| **FNTAP2** | 0.435580563 | 0.199610512 | -1.125751877 | 0.001214821 | 0.01704799 |
| **FOXJ1** | 0.493676923 | 0.149262181 | -1.725718534 | 0.000435083 | 0.0084216 |
| **FRG2C** | 0.177928717 | 0.043709089 | -2.025294154 | 0.00132159 | 0.018015841 |
| **GAD1** | 0.302823868 | 0.136696432 | -1.147503329 | 0.001795007 | 0.022370156 |
| **GALNT16** | 0.545042715 | 0.260502662 | -1.065071187 | 0.003968423 | 0.037322044 |
| **GAPDHP37** | 0.172911773 | 0.071725404 | -1.269479996 | 0.005896914 | 0.047675543 |
| **GAPDHP52** | 0.1697281 | 0.075512452 | -1.168438961 | 0.005246546 | 0.044115036 |
| **GDI2P1** | 0.173002212 | 0.067937368 | -1.348513265 | 0.001948801 | 0.023564732 |
| **GFAP** | 0.227954554 | 0.059044875 | -1.948862491 | 0.000190779 | 0.004704568 |
| **GMCL1P1** | 0.164546292 | 0.050486247 | -1.704531183 | 0.000142 | 0.003752003 |
| **GOLGA8R** | 0.150900805 | 0.072907972 | -1.049452025 | 0.000642091 | 0.011026187 |
| **GPR142** | 0.345862413 | 0.089392132 | -1.951978476 | 0.000687423 | 0.011491003 |
| **GPR17** | 0.197009406 | 0.088676745 | -1.151636785 | 0.004472413 | 0.040304367 |
| **GRAMD2** | 0.68763277 | 0.340349497 | -1.014621321 | 0.002713673 | 0.029318812 |
| **GRHL3** | 0.723499489 | 0.334130016 | -1.114582408 | 0.006360837 | 0.049927263 |
| **GRIK1** | 0.826458651 | 0.288576617 | -1.517988234 | 0.006188042 | 0.049066776 |
| **GRM4** | 0.189224764 | 0.039340976 | -2.265996256 | 0.002387235 | 0.027007988 |
| **GS1-309P15.4** | 0.338925012 | 0.150516573 | -1.171043762 | 4.10E-05 | 0.001476753 |
| **HAUS6P1** | 0.223121052 | 0.087436254 | -1.351523139 | 2.88E-05 | 0.001092546 |
| **HIGD1C** | 0.240650769 | 0.075763626 | -1.667363751 | 0.002825464 | 0.030025751 |
| **HK2P1** | 0.225495759 | 0.085238773 | -1.403518575 | 6.86E-05 | 0.0021783 |
| **HYDIN2** | 0.245310293 | 0.095880691 | -1.355295555 | 0.000744352 | 0.012169795 |
| **JPH1** | 1.244472082 | 0.465539136 | -1.418559507 | 0.001694273 | 0.021366623 |
| **KB-1958F4.2** | 0.159496075 | 0.061006942 | -1.3864756 | 0.005388298 | 0.044935095 |
| **KCTD19** | 0.22984753 | 0.060641638 | -1.92229652 | 4.74E-06 | 0.000254776 |
| **KCTD9P2** | 0.231593923 | 0.091922059 | -1.333114379 | 2.32E-05 | 0.000917619 |
| **KCTD9P4** | 0.248402373 | 0.073590689 | -1.75508382 | 0.000320082 | 0.006777256 |
| **KIF12** | 0.40856953 | 0.107457091 | -1.926820927 | 0.002675863 | 0.029096989 |
| **KLF14** | 0.20738045 | 0.070602524 | -1.554488223 | 0.004905067 | 0.042333174 |
| **KLHL10** | 0.166817024 | 0.047156145 | -1.822748824 | 0.003364452 | 0.033724626 |
| **LAMA1** | 0.400846827 | 0.113311205 | -1.822760527 | 0.003802711 | 0.036359183 |
| **LINC01411** | 0.537085472 | 0.186950831 | -1.522492811 | 0.001681489 | 0.021290433 |
| **LIPG** | 0.576618637 | 0.186673942 | -1.627096917 | 0.00032271 | 0.006806891 |
| **LRRC2** | 0.598214813 | 0.213377171 | -1.487257804 | 0.000563133 | 0.010068995 |
| **LRRN4CL** | 0.933248311 | 0.462739354 | -1.012061194 | 0.002249889 | 0.025871662 |
| **MAP3K15** | 0.330269449 | 0.086860778 | -1.926866743 | 7.82E-05 | 0.002428163 |
| **MED28P3** | 0.215045767 | 0.093164365 | -1.206793589 | 0.004917532 | 0.042333174 |
| **MEIS1-AS2** | 0.307077437 | 0.103602298 | -1.567546505 | 0.004758475 | 0.041721238 |
| **MIR1296** | 0.61056102 | 0.203053367 | -1.588276536 | 0.003938327 | 0.03710686 |
| **MIR4665** | 0.625659049 | 0.190420764 | -1.716185874 | 0.001080031 | 0.015688081 |
| **MIR92B** | 0.63543314 | 0.257573407 | -1.302756682 | 0.001765612 | 0.02207618 |
| **MIR99AHG** | 0.892893099 | 0.432591779 | -1.04548121 | 0.002473804 | 0.027527885 |
| **MLNR** | 0.203646871 | 0.048701687 | -2.064025988 | 0.001728461 | 0.021732663 |
| **MMP23B** | 1.094518348 | 0.456715044 | -1.260929924 | 0.001637779 | 0.020825814 |
| **MTUS2** | 0.167561921 | 0.044056455 | -1.927269001 | 0.003856339 | 0.036824764 |
| **NANOS1** | 0.951218695 | 0.369938302 | -1.36249239 | 4.01E-07 | 3.19E-05 |
| **NDUFAF4P4** | 0.163611278 | 0.045771867 | -1.837739161 | 0.005386674 | 0.044935095 |
| **NEB** | 0.767054171 | 0.372592469 | -1.041729953 | 0.005179413 | 0.043873899 |
| **NEUROG2** | 0.210378015 | 0.044405175 | -2.244184226 | 0.000804841 | 0.012851002 |
| **NMNAT1P1** | 0.176637331 | 0.03990678 | -2.146084491 | 0.003654531 | 0.035624604 |
| **NODAL** | 0.240592095 | 0.115282663 | -1.06141367 | 0.00171163 | 0.021573762 |
| **NR5A1** | 0.24152986 | 0.086441367 | -1.482407764 | 0.001622181 | 0.020825814 |
| **NRK** | 1.205352098 | 0.561566443 | -1.101926002 | 0.00329474 | 0.033345007 |
| **NUS1P3** | 0.171005576 | 0.03687314 | -2.213401204 | 0.003361128 | 0.03370468 |
| **OPRD1** | 0.48040745 | 0.140484619 | -1.773846337 | 0.005525375 | 0.045656193 |
| **PCA3_1** | 0.297341061 | 0.096485512 | -1.623734477 | 0.003490192 | 0.034438377 |
| **PDE6C** | 0.188720247 | 0.06704106 | -1.493132341 | 0.00438008 | 0.039568268 |
| **PGAM1P11** | 0.354401799 | 0.157985997 | -1.165589234 | 0.005453761 | 0.045361212 |
| **PHACTR3** | 0.731431184 | 0.273783824 | -1.417684924 | 0.003748887 | 0.036296238 |
| **PIP5K1P1** | 0.1455592 | 0.060008759 | -1.278361029 | 0.000110012 | 0.00314 |
| **PITPNM3** | 0.854545019 | 0.41679592 | -1.035815343 | 0.000133458 | 0.003601667 |
| **PKP1** | 1.244363567 | 0.385476323 | -1.690693907 | 0.000306423 | 0.00658883 |
| **POLHP1** | 0.189232479 | 0.061680862 | -1.617264903 | 0.000416049 | 0.008193526 |
| **POU5F1P4** | 0.401920073 | 0.170752354 | -1.235003162 | 0.003902676 | 0.036880509 |
| **PPFIA4** | 1.124486393 | 0.536822864 | -1.066748179 | 1.45E-05 | 0.000629683 |
| **PPP1R14D** | 0.31926069 | 0.092833022 | -1.782024942 | 0.006229531 | 0.049343983 |
| **PRELID1P3** | 0.321177972 | 0.131448559 | -1.288874623 | 0.001447844 | 0.0192487 |
| **PRRT4** | 0.545817968 | 0.270064703 | -1.015114792 | 0.000306488 | 0.00658883 |
| **PRSS45** | 0.275418235 | 0.135875164 | -1.019342308 | 0.004377101 | 0.039568268 |
| **PSMD10P1** | 0.668800366 | 0.316611236 | -1.078863182 | 0.000145706 | 0.003845907 |
| **PTP4A1P3** | 0.239206603 | 0.095625068 | -1.322796444 | 0.001521686 | 0.020030113 |
| **PTPRT** | 0.229952973 | 0.038714245 | -2.570402451 | 0.001162815 | 0.01654396 |
| **RCC2P4** | 0.15411643 | 0.058670784 | -1.393306503 | 0.001940205 | 0.023483271 |
| **RCC2P7** | 0.211042989 | 0.082380026 | -1.357170414 | 4.07E-05 | 0.001468272 |
| **RN7SL229P** | 0.191589759 | 0.076331829 | -1.327663777 | 0.000654972 | 0.011201485 |
| **RN7SL262P** | 0.536170525 | 0.242279277 | -1.146020899 | 0.004191196 | 0.038610366 |
| **RN7SL382P** | 0.425435522 | 0.159442258 | -1.415906451 | 0.000847628 | 0.01324124 |
| **RN7SL449P** | 0.240985107 | 0.089345748 | -1.431473019 | 0.00387506 | 0.036843705 |
| **RN7SL535P** | 0.751633065 | 0.357398503 | -1.07249494 | 0.004852361 | 0.042178469 |
| **RNA5SP311** | 0.789370137 | 0.385963878 | -1.032236106 | 0.006348793 | 0.049927263 |
| **RNF113B** | 0.179358685 | 0.040219421 | -2.156883391 | 0.000682542 | 0.011491003 |
| **RNU1-36P** | 0.61365996 | 0.230275696 | -1.414077303 | 0.003188092 | 0.032603722 |
| **RNU6-1238P** | 0.588577614 | 0.239752716 | -1.295685513 | 0.002740824 | 0.029498598 |
| **RNU6-1294P** | 0.780365703 | 0.227921247 | -1.775614956 | 0.000137628 | 0.003682708 |
| **RNU6-1297P** | 0.774347171 | 0.292596019 | -1.404070388 | 0.004368625 | 0.039568268 |
| **RNU6-577P** | 0.371975558 | 0.101860336 | -1.868615451 | 0.003180829 | 0.032555816 |
| **ROPN1B** | 0.163409648 | 0.036607835 | -2.158268808 | 0.003234388 | 0.033031635 |
| **RP1-102G20.5** | 0.371463276 | 0.139150766 | -1.416570737 | 0.005615018 | 0.046336282 |
| **RP1-120G22.11** | 1.398164172 | 0.679221771 | -1.041579164 | 0.000755693 | 0.012337888 |
| **RP1-202O8.2** | 0.323522097 | 0.069282075 | -2.223310216 | 0.006279795 | 0.049664255 |
| **RP1-290I10.2** | 0.176596891 | 0.033763406 | -2.386927583 | 0.004783922 | 0.041721238 |
| **RP1-67A8.3** | 0.531775696 | 0.243716962 | -1.125611177 | 0.001652641 | 0.020967208 |
| **RP11-100G15.12** | 0.18484111 | 0.050291563 | -1.877897365 | 0.00511536 | 0.043360399 |
| **RP11-102K13.5** | 1.6358152 | 0.809562035 | -1.014796234 | 0.001488988 | 0.01967146 |
| **RP11-10N16.3** | 0.159800103 | 0.047030939 | -1.764586288 | 0.000510844 | 0.009446497 |
| **RP11-114M5.1** | 0.223455476 | 0.105032033 | -1.089158013 | 0.003800937 | 0.036359183 |
| **RP11-158M2.3** | 0.622792447 | 0.302465086 | -1.041982831 | 0.000932762 | 0.014220286 |
| **RP11-159H10.1** | 0.176600502 | 0.062120235 | -1.507354245 | 0.000559274 | 0.010033564 |
| **RP11-159H10.4** | 0.170407046 | 0.030600827 | -2.477342448 | 0.000716991 | 0.011852879 |
| **RP11-161H23.9** | 0.291229633 | 0.089954424 | -1.694891015 | 0.000108698 | 0.003109524 |
| **RP11-162G10.1** | 0.217987773 | 0.08944271 | -1.28521141 | 0.000817473 | 0.0129382 |
| **RP11-169K17.3** | 0.185317209 | 0.055060846 | -1.750898181 | 0.003822489 | 0.03653276 |
| **RP11-18B3.2** | 0.163437744 | 0.047327949 | -1.787976899 | 0.000442245 | 0.008553682 |
| **RP11-196B3.3** | 0.290354108 | 0.116323119 | -1.319675585 | 0.003980185 | 0.037403082 |
| **RP11-203M5.2** | 0.265696189 | 0.074072206 | -1.842773329 | 0.001181047 | 0.016672468 |
| **RP11-20I23.8** | 0.24653155 | 0.110857143 | -1.15307056 | 0.002647369 | 0.028824376 |
| **RP11-210N13.1** | 0.334270966 | 0.133665179 | -1.322394368 | 0.003111686 | 0.032043723 |
| **RP11-218E20.5** | 0.578725375 | 0.259412393 | -1.1576315 | 0.002886425 | 0.03031646 |
| **RP11-21A7A.2** | 0.264631178 | 0.07913316 | -1.741628764 | 0.000460117 | 0.008786301 |
| **RP11-21A7A.4** | 0.261903249 | 0.080691701 | -1.698541758 | 0.001992926 | 0.023881082 |
| **RP11-223I10.1** | 0.199562162 | 0.067830676 | -1.556828435 | 0.001473956 | 0.019575252 |
| **RP11-231I16.1** | 0.209596071 | 0.096032612 | -1.126015347 | 0.003968423 | 0.037322044 |
| **RP11-283C24.1** | 0.256801369 | 0.124704854 | -1.042135274 | 0.000517978 | 0.009522305 |
| **RP11-286H14.8** | 0.770296921 | 0.372365924 | -1.048693606 | 6.01E-05 | 0.001960624 |
| **RP11-295B17.6** | 0.394977263 | 0.068483053 | -2.527950689 | 0.003389098 | 0.033896393 |
| **RP11-295P9.2** | 0.208750172 | 0.103583652 | -1.010981055 | 0.002413303 | 0.027165787 |
| **RP11-30L15.4** | 0.455913611 | 0.225955343 | -1.012722808 | 0.003109624 | 0.032043723 |
| **RP11-313F23.4** | 0.732946969 | 0.22510919 | -1.70308386 | 0.001372815 | 0.01853411 |
| **RP11-325P15.1** | 0.184761696 | 0.076326352 | -1.27541254 | 0.00482133 | 0.041966501 |
| **RP11-338I21.1** | 0.281338483 | 0.126552814 | -1.152567319 | 0.00437869 | 0.039568268 |
| **RP11-339B21.8** | 0.579445543 | 0.28666182 | -1.015323315 | 0.002798178 | 0.029798473 |
| **RP11-340I6.6** | 0.292249258 | 0.145130578 | -1.009847847 | 1.65E-05 | 0.000700329 |
| **RP11-361L15.5** | 0.231668111 | 0.071776664 | -1.690472698 | 0.002975791 | 0.030933532 |
| **RP11-37J13.1** | 0.231840325 | 0.115309255 | -1.007623205 | 0.00015695 | 0.004070393 |
| **RP11-383B4.4** | 0.353019754 | 0.135530604 | -1.381130259 | 0.002731159 | 0.029429884 |
| **RP11-396O20.2** | 0.175911085 | 0.027482288 | -2.678272369 | 0.001209368 | 0.016980896 |
| **RP11-39E3.4** | 0.306764277 | 0.138856067 | -1.143540272 | 0.005796026 | 0.047274015 |
| **RP11-416N2.4** | 0.716088128 | 0.351277096 | -1.027527637 | 0.004633853 | 0.041022561 |
| **RP11-428P16.2** | 0.224534215 | 0.104599648 | -1.102057307 | 0.003968607 | 0.037322044 |
| **RP11-437B10.1** | 0.295735788 | 0.138465172 | -1.094785699 | 0.001264305 | 0.017480209 |
| **RP11-443F16.1** | 0.235696372 | 0.105958473 | -1.153430592 | 0.001408278 | 0.018851665 |
| **RP11-449J10.1** | 0.277435835 | 0.090715002 | -1.612741078 | 0.00393515 | 0.037104106 |
| **RP11-454K7.1** | 0.228411423 | 0.07104354 | -1.684859421 | 0.00133222 | 0.018141176 |
| **RP11-45A17.4** | 0.431794914 | 0.193892268 | -1.155090981 | 0.000525885 | 0.009632958 |
| **RP11-473E2.3** | 0.301141578 | 0.08687931 | -1.793357366 | 0.000722786 | 0.011922896 |
| **RP11-475E11.9** | 0.276304261 | 0.115185103 | -1.262303671 | 0.000917933 | 0.014104001 |
| **RP11-48G14.1** | 0.244834702 | 0.075267589 | -1.701707399 | 6.31E-05 | 0.002039759 |
| **RP11-493P1.2** | 0.265639743 | 0.127284831 | -1.061410504 | 0.002912753 | 0.030542191 |
| **RP11-496H15.2** | 0.395223242 | 0.128468865 | -1.621249034 | 0.001998746 | 0.023928115 |
| **RP11-510I21.1** | 0.178160302 | 0.042288699 | -2.074831815 | 0.001335495 | 0.018175966 |
| **RP11-516C1.1** | 0.319511032 | 0.155421392 | -1.039680657 | 0.004574876 | 0.040762387 |
| **RP11-528A10.1** | 0.359521926 | 0.131742271 | -1.448361437 | 0.003531237 | 0.034816176 |
| **RP11-528A10.2** | 0.162674533 | 0.077447811 | -1.070692041 | 4.59E-05 | 0.001592054 |
| **RP11-542B15.1** | 0.368182937 | 0.155283094 | -1.245522002 | 0.000781458 | 0.012613187 |
| **RP11-543C4.3** | 0.180173976 | 0.045300687 | -1.991785808 | 0.002759238 | 0.029671502 |
| **RP11-54D18.4** | 0.736548455 | 0.351531056 | -1.067128288 | 8.21E-06 | 0.000397203 |
| **RP11-54O15.3** | 0.276403906 | 0.065587034 | -2.07529547 | 0.001159411 | 0.016527497 |
| **RP11-554A11.4** | 0.699129539 | 0.259633698 | -1.429082147 | 0.005452858 | 0.045361212 |
| **RP11-561O4.1** | 0.25877516 | 0.082984814 | -1.640779885 | 0.002449344 | 0.02751019 |
| **RP11-567M16.1** | 0.22026961 | 0.05280272 | -2.06058631 | 0.002420811 | 0.027226038 |
| **RP11-5P18.10** | 0.608240089 | 0.2359816 | -1.365966535 | 0.000869647 | 0.013534983 |
| **RP11-611L7.3** | 0.308453455 | 0.112899565 | -1.450012874 | 0.004805071 | 0.041868261 |
| **RP11-619A14.2** | 0.215383985 | 0.057232486 | -1.912004803 | 0.004038097 | 0.037611482 |
| **RP11-642A1.1** | 0.167360555 | 0.059605658 | -1.489438354 | 0.002367583 | 0.026830483 |
| **RP11-697E2.12** | 0.229681386 | 0.069606156 | -1.722347133 | 0.002166882 | 0.025397429 |
| **RP11-701H24.5** | 0.747079396 | 0.368712057 | -1.018766981 | 0.00505328 | 0.043016774 |
| **RP11-73M18.11** | 0.351193496 | 0.095944002 | -1.872001604 | 6.99E-05 | 0.002213382 |
| **RP11-75A9.3** | 0.169489392 | 0.08416931 | -1.009828794 | 0.000389159 | 0.007753427 |
| **RP11-76E16.2** | 0.254522581 | 0.106920267 | -1.251258315 | 0.001989865 | 0.023855719 |
| **RP11-76P2.4** | 0.302333454 | 0.149464094 | -1.016341684 | 0.005458844 | 0.045388547 |
| **RP11-777F6.3** | 0.480507451 | 0.167358555 | -1.521616504 | 0.000155886 | 0.004055287 |
| **RP11-797A18.5** | 0.398934449 | 0.1694003 | -1.235715283 | 0.000351294 | 0.007249741 |
| **RP11-80I15.4** | 0.29629443 | 0.136504834 | -1.118079461 | 0.000343826 | 0.007148188 |
| **RP11-829H16.3** | 0.316212423 | 0.108265876 | -1.54631545 | 0.00482039 | 0.041966501 |
| **RP11-844P9.2** | 0.275695265 | 0.11834543 | -1.220070494 | 0.000869594 | 0.013534983 |
| **RP11-894P9.2** | 0.479463299 | 0.192319688 | -1.317913923 | 0.00136579 | 0.018449124 |
| **RP11-958F21.1** | 1.02589708 | 0.422205821 | -1.280867628 | 0.000942328 | 0.01430481 |
| **RP11-95I16.6** | 0.305202576 | 0.074933717 | -2.026080219 | 0.005644563 | 0.046519302 |
| **RP3-329A5.1** | 0.373420947 | 0.132534279 | -1.494437305 | 0.004608701 | 0.041020362 |
| **RP3-347M6.2** | 0.323928602 | 0.155037008 | -1.063063225 | 0.000276269 | 0.0060948 |
| **RP3-425P12.4** | 0.440298414 | 0.216391869 | -1.024835356 | 0.000271512 | 0.006000348 |
| **RP3-467K16.2** | 0.179824347 | 0.057795144 | -1.637568184 | 0.004599863 | 0.04095613 |
| **RP3-511B24.5** | 0.305098427 | 0.132032235 | -1.208384542 | 0.005519111 | 0.045656193 |
| **RP3-522J7.5** | 0.415739057 | 0.165260949 | -1.330932435 | 0.001837917 | 0.022668845 |
| **RP4-530I15.9** | 0.178400392 | 0.079592045 | -1.164422648 | 0.002317808 | 0.02639212 |
| **RP5-1039K5.16** | 0.168563852 | 0.04309675 | -1.967644216 | 0.002435882 | 0.027371162 |
| **RP5-1121H13.4** | 0.228693716 | 0.043808002 | -2.384150409 | 0.002535975 | 0.028108126 |
| **RP5-1182A14.5** | 0.319385946 | 0.085430971 | -1.902469744 | 6.65E-05 | 0.002131469 |
| **RP5-875O13.7** | 0.634844697 | 0.227288364 | -1.481879882 | 0.005026892 | 0.043016774 |
| **RPL36P2** | 0.364937084 | 0.141202463 | -1.369882509 | 0.004572106 | 0.040752078 |
| **RPL7P41** | 0.264003514 | 0.032867883 | -3.005806703 | 0.001139582 | 0.016346983 |
| **RPL7P49** | 0.351133803 | 0.168931245 | -1.055584698 | 0.001681401 | 0.021290433 |
| **RPRML** | 0.312096334 | 0.106336913 | -1.553348918 | 0.006116296 | 0.048845284 |
| **RYR3** | 0.296103981 | 0.109083743 | -1.440667776 | 0.001965685 | 0.023734798 |
| **SCDP1** | 0.682476693 | 0.252087438 | -1.436855551 | 4.26E-05 | 0.001515198 |
| **SCRG1** | 0.942990359 | 0.359749819 | -1.390249061 | 0.003109624 | 0.032043723 |
| **SHANK2** | 0.184247766 | 0.059483958 | -1.631074571 | 0.005703197 | 0.046701818 |
| **SLC16A12** | 0.241931082 | 0.088897411 | -1.444382816 | 0.002019958 | 0.024159156 |
| **SLC18A2** | 0.192523894 | 0.093406673 | -1.043439979 | 0.001075913 | 0.015641841 |
| **SNX6P1** | 0.214624867 | 0.076759345 | -1.483402927 | 0.005405421 | 0.044988776 |
| **SOWAHA** | 0.240487417 | 0.079278532 | -1.60095926 | 0.002457192 | 0.027527885 |
| **SOX6** | 1.300670718 | 0.63175895 | -1.041809668 | 4.15E-05 | 0.001483176 |
| **SOX9-AS1** | 0.366342479 | 0.148434063 | -1.303370796 | 0.004899769 | 0.042333174 |
| **SP3P** | 0.209946137 | 0.039226224 | -2.42012887 | 0.001743942 | 0.021887771 |
| **SP5** | 0.558239827 | 0.236012156 | -1.242023887 | 0.004942072 | 0.042447042 |
| **SRP72P1** | 0.412579448 | 0.123698554 | -1.737843324 | 0.000113101 | 0.003206443 |
| **SRPK2P** | 0.275876035 | 0.129346274 | -1.092781637 | 0.003938381 | 0.03710686 |
| **SRRM1P3** | 0.20341441 | 0.098685008 | -1.04351905 | 6.25E-06 | 0.000319034 |
| **STX18-IT1** | 0.209767882 | 0.077163628 | -1.442800924 | 0.000858806 | 0.01339101 |
| **SULT4A1** | 0.534671044 | 0.193111565 | -1.469216985 | 0.004920218 | 0.042333174 |
| **TET1** | 0.922630377 | 0.419139411 | -1.138322612 | 3.74E-08 | 4.39E-06 |
| **TEX36** | 0.165925617 | 0.053050906 | -1.645087335 | 0.006357653 | 0.049927263 |
| **TMEM184A** | 0.532038401 | 0.091247844 | -2.543667998 | 0.002216332 | 0.025741353 |
| **TMEM75** | 0.190928864 | 0.076188547 | -1.325389181 | 0.002229345 | 0.025867364 |
| **TRIM29** | 0.204714906 | 0.051854728 | -1.981068705 | 0.000183894 | 0.004576586 |
| **TRIM9** | 0.90655833 | 0.370785263 | -1.289815946 | 0.000708932 | 0.011779242 |
| **TRPV3** | 0.435426237 | 0.138377963 | -1.653814132 | 0.000334006 | 0.006984268 |
| **TTC21B-AS1** | 0.178098042 | 0.042478741 | -2.067858735 | 0.00536528 | 0.044808618 |
| **UBE3AP2** | 0.232424299 | 0.099138904 | -1.229237684 | 8.75E-05 | 0.002650216 |
| **USH1C** | 0.433676336 | 0.087598996 | -2.307632476 | 0.002596736 | 0.028556181 |
| **WDR66** | 0.45980291 | 0.201223332 | -1.192217999 | 0.000232191 | 0.005375926 |
| **WNK2** | 0.652613485 | 0.278104936 | -1.230599446 | 0.003356998 | 0.033676641 |
| **XRCC6P4** | 0.333129209 | 0.162285291 | -1.037549612 | 0.002050693 | 0.024251174 |
| **XXbac-B461K10.4** | 0.152467873 | 0.059021209 | -1.369199888 | 0.004760462 | 0.041721238 |
| **XXbac-B476C20.11** | 0.529356812 | 0.254061512 | -1.05906266 | 0.00489535 | 0.042333174 |
| **ZBED6** | 1.547189682 | 0.754467348 | -1.036119708 | 1.58E-07 | 1.46E-05 |
| **ZBTB40-IT1** | 0.351509689 | 0.157738868 | -1.156026267 | 0.00213666 | 0.025126648 |
| **ZNF341-AS1** | 0.645040781 | 0.278994429 | -1.209154057 | 0.004109776 | 0.038171257 |
