# Supplementary material for: A three-gene signature based on tumour microenvironment predicts overall survival of osteosarcoma in adolescents and young adults
Source: Aging (Albany NY). 2020 Dec 3;13(1):619–45. doi: 10.18632/aging.202170 (PMC7835013; doi:10.18632/aging.202170)
Supplement: Supplementary Table 3A and 3B [file aging-13-202170-s004.doc]

**Supplementary Table3 A. 160 differential genes up-regulated in the high stromal score group.**

| **Gene** | **Low stromal score** | **High stromal score** | **Log FC** | **P Value** | **FDR** |
| --- | --- | --- | --- | --- | --- |
| **AC005498.4** | 0.07898104 | 0.165157388 | 1.064263232 | 0.003334804 | 0.037649375 |
| **AC020931.1** | 0.162116658 | 0.42839035 | 1.401893647 | 0.004068811 | 0.041538713 |
| **AC027319.1** | 0.121537838 | 0.319739162 | 1.395489931 | 0.001606124 | 0.025722335 |
| **AC091177.1** | 0.141413165 | 0.315001697 | 1.155443166 | 0.00060266 | 0.01504103 |
| **AC243945.1** | 0.949524073 | 2.137071119 | 1.170358437 | 0.000300501 | 0.009936745 |
| **ACKR1** | 0.20023254 | 0.777338243 | 1.956866049 | 2.55E-05 | 0.002296091 |
| **ACSM5** | 0.46355402 | 0.961165874 | 1.052047954 | 0.000312966 | 0.010162384 |
| **ACTG2** | 0.756275746 | 2.01243951 | 1.411961161 | 1.52E-05 | 0.001623039 |
| **AL034375.1** | 0.136022478 | 0.422310363 | 1.634458565 | 0.000368312 | 0.011102155 |
| **ALAS2** | 0.088905435 | 0.216037159 | 1.280935963 | 0.003830744 | 0.040474063 |
| **AP001189.4** | 0.093450597 | 0.354989038 | 1.925498696 | 3.26E-07 | 0.000192317 |
| **APCDD1L** | 0.91254845 | 2.001332628 | 1.132987904 | 0.001441337 | 0.024159929 |
| **ARHGEF34P** | 0.119705535 | 0.255030691 | 1.091181006 | 0.000212142 | 0.008091498 |
| **BHLHE22** | 0.115042763 | 0.604494537 | 2.393559076 | 0.000109214 | 0.005354505 |
| **C1QTNF7** | 0.468450142 | 1.019141354 | 1.121386752 | 0.001001512 | 0.019828823 |
| **C2orf40** | 0.239359681 | 0.58395889 | 1.286686648 | 0.003438592 | 0.038192775 |
| **C3** | 0.954501161 | 2.160839302 | 1.178772927 | 5.27E-07 | 0.000237495 |
| **CCIN** | 0.159841855 | 0.326492319 | 1.030403823 | 0.000475741 | 0.012946418 |
| **CCL14** | 0.262965517 | 0.566806247 | 1.107982029 | 0.000652919 | 0.015756435 |
| **CCL19** | 0.207932288 | 0.521451943 | 1.326420505 | 0.005279965 | 0.047700426 |
| **CCR2** | 0.173136775 | 0.37970921 | 1.132982799 | 0.000197965 | 0.007769414 |
| **CD80** | 0.246467059 | 0.49421806 | 1.00375289 | 0.003489679 | 0.038362552 |
| **CEACAM4** | 0.106010783 | 0.269950003 | 1.34848122 | 0.000293553 | 0.009762897 |
| **CECR5-AS1** | 0.185039904 | 0.413300197 | 1.159353628 | 0.003167506 | 0.036514631 |
| **CH25H** | 1.030225909 | 2.089266511 | 1.020035809 | 1.39E-05 | 0.001556487 |
| **CHRNA9** | 0.100755839 | 0.227216073 | 1.173201452 | 0.002204793 | 0.030169056 |
| **COMP** | 1.758804978 | 3.584525047 | 1.027186452 | 0.001264305 | 0.022794241 |
| **CPB1** | 0.209237962 | 0.476626774 | 1.187715372 | 0.001533579 | 0.025094974 |
| **CRISPLD2** | 1.485808474 | 3.202892422 | 1.108127183 | 1.31E-10 | 1.09E-06 |
| **CRYGN** | 0.119916049 | 0.358421397 | 1.579632005 | 0.001042012 | 0.020271923 |
| **CTB-108O6.2** | 0.098947394 | 0.219802127 | 1.151471732 | 0.000801125 | 0.01796953 |
| **CTD-2078B5.2** | 0.38151186 | 0.783878514 | 1.038902177 | 0.000347639 | 0.010782403 |
| **CTD-2325P2.4** | 0.138146117 | 0.329833788 | 1.255544186 | 0.002661269 | 0.033310178 |
| **CTD-2339F6.1** | 0.266869132 | 0.764174359 | 1.517769411 | 0.003012492 | 0.035604522 |
| **CTF1** | 0.499587267 | 1.106198227 | 1.146801322 | 9.97E-07 | 0.000338714 |
| **CX3CR1** | 0.583872346 | 1.409199896 | 1.271151386 | 1.29E-06 | 0.000400824 |
| **EGFL6** | 1.456369877 | 3.707976664 | 1.348255358 | 1.10E-05 | 0.001418611 |
| **EOMES** | 0.223467008 | 0.528409315 | 1.241594049 | 0.001516166 | 0.025040534 |
| **FCGR1A** | 0.828056996 | 1.754257081 | 1.083058207 | 1.55E-07 | 0.00014766 |
| **FCGR1B** | 0.205244043 | 0.451117555 | 1.13616308 | 6.99E-05 | 0.004269342 |
| **FCGR1C** | 0.329211895 | 0.824036802 | 1.323692309 | 1.27E-05 | 0.001488582 |
| **FCGR2B** | 0.413515013 | 0.896335983 | 1.116099905 | 6.83E-08 | 8.07E-05 |
| **FGF10** | 0.065373784 | 0.175055791 | 1.421030674 | 0.003808771 | 0.040293387 |
| **FGF7** | 0.318716714 | 0.716774155 | 1.16924394 | 0.001146324 | 0.021462649 |
| **FMO3** | 0.456354328 | 0.955440999 | 1.066012374 | 0.00139508 | 0.023950959 |
| **GALNT15** | 0.439317891 | 0.936726518 | 1.092362652 | 6.05E-05 | 0.003840389 |
| **GAPT** | 0.152429379 | 0.501106398 | 1.716975966 | 1.13E-06 | 0.000360768 |
| **GGTA1P** | 0.769469943 | 1.553691103 | 1.013762825 | 1.84E-07 | 0.000150704 |
| **GIMAP5** | 0.17458062 | 0.359722647 | 1.042991572 | 5.90E-05 | 0.003821163 |
| **GTF2F2P1** | 0.189898257 | 0.391793633 | 1.044867292 | 0.000628825 | 0.015445434 |
| **HBA1** | 1.160243664 | 2.340847223 | 1.01260296 | 0.001107327 | 0.021018024 |
| **HBD** | 0.052578065 | 0.14932564 | 1.505928943 | 0.001293488 | 0.0231364 |
| **HEPH** | 0.833450782 | 1.673848062 | 1.005999667 | 0.000223581 | 0.008303302 |
| **HTR2A** | 0.219115493 | 0.516918522 | 1.238245399 | 0.005251541 | 0.047495522 |
| **HTR2B** | 0.254762318 | 0.612242117 | 1.264950392 | 4.96E-07 | 0.000229153 |
| **IGFL3** | 0.059939816 | 0.267527115 | 2.158098563 | 0.001300734 | 0.0231364 |
| **IGHG1** | 1.079594028 | 2.199172252 | 1.026471708 | 0.000675041 | 0.016133354 |
| **IGHG2** | 0.842913701 | 1.707140089 | 1.018124614 | 0.002298407 | 0.030837684 |
| **IGHG3** | 0.624051051 | 1.462345567 | 1.228548316 | 0.002095165 | 0.029445505 |
| **IGHG4** | 0.390137787 | 1.018674167 | 1.384637023 | 0.000475941 | 0.012946418 |
| **IGHM** | 0.642956087 | 1.682278788 | 1.387624699 | 0.003019267 | 0.035649332 |
| **IGHV1-2** | 0.303946158 | 0.756795883 | 1.316088458 | 0.00343106 | 0.038192775 |
| **IGHV1-24** | 0.199154434 | 0.430935481 | 1.11358429 | 0.005073711 | 0.046566266 |
| **IGHV1-69-2** | 0.222927524 | 0.746417187 | 1.743407454 | 0.00020235 | 0.007853688 |
| **IGHV3-23** | 0.551119598 | 1.243758303 | 1.17426882 | 0.001016556 | 0.020078603 |
| **IGHV3-48** | 0.148373572 | 0.345731503 | 1.220417921 | 0.000434098 | 0.012206277 |
| **IGHV3-53** | 0.118355773 | 0.364049154 | 1.621003184 | 0.003058652 | 0.035727862 |
| **IGKV1-16** | 0.207856602 | 0.516202342 | 1.312348119 | 0.002106083 | 0.029445505 |
| **IGKV1-9** | 0.249632682 | 0.677175214 | 1.439722344 | 0.000881332 | 0.018751358 |
| **IGKV2-24** | 0.205023823 | 0.520818192 | 1.344988287 | 0.002228968 | 0.030330536 |
| **IGKV3-11** | 0.584333509 | 1.189640147 | 1.025661311 | 0.000490899 | 0.013222839 |
| **IGKV3-20** | 0.706562364 | 1.527401214 | 1.112190267 | 0.000862791 | 0.018628474 |
| **IGKV4-1** | 0.615773235 | 1.544555944 | 1.32672106 | 0.00235265 | 0.031227678 |
| **IGLV1-47** | 0.293300097 | 0.856798966 | 1.546579193 | 0.000384549 | 0.011444561 |
| **IGLV2-11** | 0.316118452 | 0.895393807 | 1.502057092 | 0.004888343 | 0.046022934 |
| **IGLV2-14** | 0.56197401 | 1.44317122 | 1.360667158 | 0.00064436 | 0.015580192 |
| **IGLV2-23** | 0.370009888 | 0.763815568 | 1.0456605 | 0.001645176 | 0.025831347 |
| **IGLV3-10** | 0.216424039 | 0.524711075 | 1.277662489 | 0.002699726 | 0.033443022 |
| **IGLV3-25** | 0.32109931 | 0.902476443 | 1.490869711 | 0.000104242 | 0.005234869 |
| **IL10** | 0.253285672 | 0.555989565 | 1.134292339 | 5.46E-06 | 0.00096809 |
| **IL2RA** | 0.347862497 | 0.714546826 | 1.038511406 | 4.33E-05 | 0.003150726 |
| **IL33** | 0.161229986 | 0.439076827 | 1.445353309 | 5.19E-05 | 0.003554902 |
| **INSRR** | 0.480269855 | 0.981402468 | 1.030999639 | 0.001340435 | 0.023472481 |
| **ISLR** | 1.890301229 | 4.591050344 | 1.280208099 | 2.18E-07 | 0.000154287 |
| **ISLR2** | 0.166040484 | 0.354506546 | 1.094277224 | 0.000216251 | 0.008116131 |
| **KB-1448A5.1** | 0.100656528 | 0.274619198 | 1.447991745 | 0.000634594 | 0.015540975 |
| **KCNE4** | 0.694137068 | 1.422693232 | 1.035332136 | 1.59E-05 | 0.001669037 |
| **KIF26B** | 0.452145485 | 0.984805834 | 1.12305225 | 8.07E-05 | 0.004507046 |
| **KLHL4** | 0.354830924 | 0.804360558 | 1.180710595 | 0.000244529 | 0.008881827 |
| **LILRA1** | 0.157855722 | 0.420603146 | 1.413853086 | 4.43E-06 | 0.000845177 |
| **LILRA2** | 0.189241742 | 0.412788762 | 1.125173353 | 2.37E-05 | 0.002200849 |
| **LLNLR-470E3.1** | 0.307965191 | 0.630983628 | 1.034835282 | 0.005670699 | 0.049461797 |
| **LRRC55** | 0.083831931 | 0.20622242 | 1.298629417 | 0.001055858 | 0.020479855 |
| **LYVE1** | 0.87152694 | 1.803071503 | 1.048839444 | 5.97E-11 | 1.09E-06 |
| **MCHR1** | 0.251919999 | 0.574954335 | 1.190481721 | 0.002976811 | 0.035234895 |
| **MFAP4** | 1.563304185 | 3.639067211 | 1.218970175 | 0.000450361 | 0.012469363 |
| **MIR374B** | 0.216368822 | 0.479403726 | 1.147748496 | 0.000963695 | 0.019514904 |
| **MIR4715** | 0.057508947 | 0.383057117 | 2.735701209 | 8.14E-05 | 0.004535772 |
| **MIR5190** | 0.493537831 | 1.09301774 | 1.147084237 | 0.001224849 | 0.022392086 |
| **MRVI1** | 0.418006772 | 0.846721122 | 1.018360563 | 1.05E-05 | 0.001413406 |
| **MS4A14** | 0.384074597 | 0.807572455 | 1.072205158 | 3.71E-06 | 0.000736591 |
| **OR2A9P** | 0.106852226 | 0.23737967 | 1.151579416 | 0.000375506 | 0.011208528 |
| **OR51E1** | 0.247270274 | 0.560408632 | 1.180390365 | 5.57E-05 | 0.00367545 |
| **P2RY10** | 0.1267418 | 0.261833831 | 1.046759114 | 0.000247446 | 0.00892298 |
| **PLA2G2A** | 0.116977189 | 0.520434689 | 2.153489895 | 0.002152104 | 0.029893272 |
| **PLD4** | 0.296844136 | 0.663865745 | 1.161185898 | 7.61E-06 | 0.001151393 |
| **PLEKHS1** | 0.189837906 | 0.502817961 | 1.405268095 | 1.22E-05 | 0.001471747 |
| **PRDX3P4** | 0.12462782 | 0.302474447 | 1.27918712 | 7.38E-05 | 0.004422857 |
| **RN7SKP275** | 0.077255071 | 0.202527695 | 1.390417658 | 0.003585626 | 0.038860732 |
| **RN7SL187P** | 0.096252095 | 0.33984178 | 1.819973372 | 2.86E-05 | 0.002389554 |
| **RNA5SP296** | 0.408198813 | 0.824806722 | 1.014784103 | 0.000597331 | 0.014975442 |
| **RNASE2** | 0.470606149 | 1.104933984 | 1.2313681 | 0.000327546 | 0.010471328 |
| **RNU5B-4P** | 0.25490834 | 0.516913537 | 1.019944405 | 0.005599296 | 0.04932789 |
| **RNU6-856P** | 0.384873653 | 0.821331747 | 1.093580152 | 0.001469184 | 0.024565044 |
| **RP1-102K2.9** | 0.087155988 | 0.192279166 | 1.14153075 | 0.000825305 | 0.018135899 |
| **RP11-1180F24.1** | 0.088206033 | 0.190028757 | 1.107268517 | 0.000163065 | 0.006829901 |
| **RP11-1260E13.4** | 0.080569653 | 0.256391212 | 1.670038368 | 0.000154373 | 0.006535294 |
| **RP11-14D22.1** | 0.145843141 | 0.338044079 | 1.212793841 | 4.17E-05 | 0.003085987 |
| **RP11-20J15.3** | 0.086635812 | 0.286148146 | 1.723726856 | 0.000176924 | 0.007173693 |
| **RP11-227G15.11** | 0.223181222 | 0.466013981 | 1.062157592 | 0.000825356 | 0.018135899 |
| **RP11-247C2.2** | 0.084140983 | 0.268136463 | 1.672086849 | 0.001798137 | 0.027108391 |
| **RP11-24F11.2** | 0.254111908 | 0.538759005 | 1.084176092 | 0.000399761 | 0.011687147 |
| **RP11-426C22.4** | 0.541651908 | 1.090615509 | 1.00970467 | 5.86E-07 | 0.000259798 |
| **RP11-426C22.5** | 0.372742938 | 0.754004744 | 1.016392581 | 1.11E-06 | 0.000356452 |
| **RP11-426C22.6** | 0.100532819 | 0.230983406 | 1.200122666 | 0.001533732 | 0.025094974 |
| **RP11-443C10.1** | 0.235865923 | 0.490129742 | 1.055196694 | 0.001313156 | 0.02321936 |
| **RP11-455F5.5** | 0.094377487 | 0.221431788 | 1.230347675 | 0.000475941 | 0.012946418 |
| **RP11-494O16.3** | 0.277608057 | 0.62608258 | 1.173303521 | 0.000157306 | 0.006636828 |
| **RP11-536O18.1** | 0.217888195 | 0.516300783 | 1.244623752 | 0.000267413 | 0.009265352 |
| **RP11-542A14.1** | 0.150021153 | 0.406516933 | 1.438149508 | 0.002092624 | 0.029445505 |
| **RP11-554A11.5** | 0.082772766 | 0.214242718 | 1.372018087 | 0.00036451 | 0.011001208 |
| **RP11-667K14.14** | 0.219212869 | 0.445181622 | 1.022061545 | 0.000820059 | 0.018057357 |
| **RP11-672A2.4** | 0.207248953 | 0.527328008 | 1.347335809 | 2.03E-05 | 0.002005065 |
| **RP11-703I16.3** | 0.836585017 | 2.42249472 | 1.533909456 | 4.96E-06 | 0.000904901 |
| **RP11-733O18.1** | 0.404127356 | 0.948837544 | 1.231351082 | 1.04E-06 | 0.000347053 |
| **RP11-815J4.6** | 0.203691048 | 0.635151251 | 1.640717609 | 4.10E-05 | 0.00308336 |
| **RP11-84C10.2** | 0.086179276 | 0.205494429 | 1.253686394 | 0.002699726 | 0.033443022 |
| **RP11-863P13.3** | 0.546925275 | 1.271414329 | 1.217018612 | 2.19E-05 | 0.00210196 |
| **RP11-87N3.6** | 0.126262782 | 0.336457494 | 1.413994812 | 0.000873102 | 0.01862986 |
| **RP4-555L14.4** | 0.144852088 | 0.294258483 | 1.022503523 | 0.001389998 | 0.023950959 |
| **RP5-1024N4.4** | 0.072914774 | 0.15290443 | 1.068347139 | 0.002945004 | 0.035226455 |
| **RP5-1031D4.3** | 0.192137785 | 0.451670543 | 1.233129568 | 0.000121764 | 0.005667392 |
| **RPL32P1** | 0.109635336 | 0.238206361 | 1.119499084 | 7.86E-05 | 0.004507046 |
| **SCG2** | 0.365438696 | 0.782806789 | 1.099026861 | 0.000187589 | 0.007481832 |
| **SELE** | 0.253430587 | 0.554614724 | 1.129895263 | 0.000889022 | 0.018882593 |
| **SEMA5B** | 0.159502892 | 0.377543964 | 1.243062073 | 1.68E-05 | 0.00172657 |
| **SLC52A3** | 0.214734348 | 0.48804219 | 1.184452891 | 3.08E-05 | 0.002518698 |
| **SLPI** | 1.164411581 | 2.380434206 | 1.031623661 | 0.000601733 | 0.015033034 |
| **SYNDIG1** | 0.234777812 | 0.48374693 | 1.042956437 | 0.000468266 | 0.012807874 |
| **TLR7** | 0.456170719 | 1.106566865 | 1.278444881 | 3.51E-08 | 5.47E-05 |
| **TLR8** | 0.248719409 | 0.558288757 | 1.16649241 | 3.71E-06 | 0.000736591 |
| **TMEM30B** | 0.561726058 | 1.204250923 | 1.100197394 | 8.07E-05 | 0.004507046 |
| **TNFSF8** | 0.424736507 | 0.945768621 | 1.15491916 | 2.28E-08 | 5.14E-05 |
| **TRBV29-1** | 0.07055391 | 0.250646727 | 1.828857459 | 0.00342892 | 0.038192775 |
| **TRBV7-3** | 0.099944672 | 0.216780011 | 1.117030163 | 0.001645176 | 0.025831347 |
| **TRDC** | 0.500617442 | 1.280949701 | 1.355433364 | 0.000687822 | 0.01623549 |
| **VENTX** | 0.258924838 | 0.587074205 | 1.181009504 | 9.30E-06 | 0.001318637 |
| **WISP2** | 0.82353117 | 1.716187811 | 1.059312282 | 0.000336871 | 0.010578595 |
| **XG** | 0.443566923 | 1.002628055 | 1.176562817 | 0.000126351 | 0.005742223 |
| **XPNPEP2** | 0.657279814 | 1.383562352 | 1.07380808 | 0.002150811 | 0.029892057 |

**Supplementary Table3 B. 251 differential genes down-regulated in the high stromal score group.**

| **Gene** | **Low stromal score** | **High stromal score** | **Log FC** | **P Value** | **FDR** |
| --- | --- | --- | --- | --- | --- |
| **AC002314.4** | 0.18482233 | 0.06242455 | -1.565953646 | 0.000734907 | 0.016962898 |
| **AC003988.1** | 0.459727807 | 0.225793361 | -1.025776866 | 0.003370593 | 0.037767691 |
| **AC004862.6** | 0.870667004 | 0.395335048 | -1.139045189 | 0.00015123 | 0.006444642 |
| **AC004920.2** | 0.258956712 | 0.089908376 | -1.526183524 | 0.004021469 | 0.041140044 |
| **AC005062.2** | 0.287702947 | 0.116397668 | -1.305517846 | 0.00496793 | 0.046284377 |
| **AC005229.7** | 0.281999474 | 0.098209925 | -1.521751745 | 0.000556174 | 0.014348645 |
| **AC005235.1** | 0.841652301 | 0.36567418 | -1.202665595 | 0.004013054 | 0.041083875 |
| **AC005307.1** | 0.197053113 | 0.056698034 | -1.797213921 | 0.001223113 | 0.02237959 |
| **AC005740.3** | 0.20629166 | 0.071317587 | -1.532355699 | 0.000692248 | 0.016308914 |
| **AC005786.3** | 0.795771411 | 0.322288765 | -1.304000174 | 0.000982193 | 0.019697858 |
| **AC006373.1** | 1.376419206 | 0.522499226 | -1.397419123 | 0.004415023 | 0.043463451 |
| **AC008694.2** | 0.47486801 | 0.223416125 | -1.087793256 | 0.000364519 | 0.011001208 |
| **AC009517.2** | 0.303680781 | 0.105814482 | -1.521018513 | 8.38E-05 | 0.004630437 |
| **AC012363.13** | 0.231217166 | 0.034721272 | -2.735356818 | 0.003293657 | 0.037443812 |
| **AC013470.6** | 0.513790444 | 0.187654202 | -1.453103459 | 2.46E-05 | 0.00224077 |
| **AC019181.3** | 0.382189788 | 0.187330597 | -1.028702674 | 0.005700869 | 0.049541657 |
| **AC022153.1** | 0.236825176 | 0.043369636 | -2.449065214 | 0.004209156 | 0.042361351 |
| **AC074117.13** | 0.456646171 | 0.183841022 | -1.312618016 | 0.003875598 | 0.040556728 |
| **AC079834.1** | 0.625356447 | 0.305162375 | -1.035101649 | 0.005311835 | 0.047894141 |
| **AC092573.2** | 0.200917091 | 0.07660549 | -1.391080601 | 0.00291112 | 0.034888434 |
| **ACTR3P2** | 0.251764625 | 0.109901235 | -1.195867993 | 0.001423376 | 0.024159929 |
| **ADAM29** | 0.319553129 | 0.119587284 | -1.41799182 | 0.004252828 | 0.042550732 |
| **ANKRD20A10P** | 0.195768373 | 0.054273632 | -1.850824336 | 0.004236184 | 0.042550732 |
| **AP000343.1** | 0.309856317 | 0.114156996 | -1.4405801 | 0.002022748 | 0.028872458 |
| **APOH** | 0.212347368 | 0.059421958 | -1.837358182 | 0.003469306 | 0.038280121 |
| **ARMC4** | 1.02823683 | 0.511484527 | -1.00741009 | 0.000130289 | 0.005823817 |
| **ASS1P10** | 0.150510893 | 0.049774459 | -1.596390367 | 0.000190922 | 0.007573317 |
| **ASS1P5** | 0.196009704 | 0.05280516 | -1.89217427 | 0.00430091 | 0.042953692 |
| **ATP5G1P7** | 0.381616112 | 0.097785586 | -1.964428359 | 0.000265011 | 0.009207832 |
| **C14orf180** | 0.944283443 | 0.445954401 | -1.082323773 | 0.001613238 | 0.025770258 |
| **C17orf102** | 0.167769936 | 0.033534615 | -2.322761277 | 0.003111614 | 0.036155933 |
| **CASKIN1** | 0.360931846 | 0.155570151 | -1.214161159 | 0.004082325 | 0.041612012 |
| **CCDC141** | 0.249520658 | 0.059197091 | -2.075561077 | 0.001426242 | 0.024159929 |
| **CCDC144NL** | 0.399241573 | 0.127753392 | -1.643900363 | 0.00251781 | 0.032464572 |
| **CCDC58P1** | 0.434860047 | 0.163057316 | -1.415171997 | 5.07E-06 | 0.000917408 |
| **CCDC58P3** | 0.3787835 | 0.176352111 | -1.102914637 | 0.003282409 | 0.037370355 |
| **CDC20B** | 0.29295457 | 0.082464816 | -1.828826338 | 0.000402833 | 0.011687147 |
| **CDKL4** | 0.234327458 | 0.092538242 | -1.340404424 | 0.005129654 | 0.046935013 |
| **CHD5** | 0.241239126 | 0.093307888 | -1.370392958 | 0.003802711 | 0.040265496 |
| **CKMT1B** | 0.296804064 | 0.071122454 | -2.061133839 | 0.002798803 | 0.034102511 |
| **CNKSR2** | 1.078108993 | 0.519371044 | -1.053665548 | 0.003066133 | 0.035727862 |
| **COCH** | 1.921141081 | 0.8352539 | -1.201676749 | 0.000936197 | 0.019210235 |
| **COL4A4** | 0.47925454 | 0.186607269 | -1.360786911 | 0.003064814 | 0.035727862 |
| **COLEC10** | 0.354273053 | 0.096146258 | -1.88155912 | 1.26E-05 | 0.001488561 |
| **COX6B1P4** | 0.376270958 | 0.127709214 | -1.558909322 | 0.005130916 | 0.046935013 |
| **CRYBA2** | 0.758096449 | 0.253509023 | -1.580344309 | 0.001328065 | 0.023432881 |
| **CSPG5** | 0.681090581 | 0.304029428 | -1.163635709 | 5.81E-05 | 0.003781155 |
| **CTB-1048E9.7** | 0.373849081 | 0.145029657 | -1.366108046 | 0.003470332 | 0.038280121 |
| **CTB-1144G6.5** | 0.322312684 | 0.103544315 | -1.638212622 | 0.00010688 | 0.005251092 |
| **CTB-4E7.1** | 1.770093051 | 0.776260137 | -1.189213094 | 0.002170239 | 0.029898527 |
| **CTD-2140G10.2** | 0.513143176 | 0.256028607 | -1.003056401 | 0.003704453 | 0.039680511 |
| **CTD-2312P21.1** | 0.326482356 | 0.125148652 | -1.383362279 | 0.002879009 | 0.03465973 |
| **CTD-2515H24.1** | 0.297436721 | 0.139489958 | -1.092421509 | 0.002354041 | 0.031229442 |
| **CTD-2651B20.2** | 0.188750512 | 0.057947634 | -1.703658891 | 0.002716494 | 0.033461169 |
| **CYCSP39** | 0.18912977 | 0.077120293 | -1.29419402 | 0.001412144 | 0.024159929 |
| **DNAI1** | 0.33451946 | 0.162298121 | -1.04344384 | 0.002976374 | 0.035234895 |
| **DUSP26** | 0.754212704 | 0.300632799 | -1.326969034 | 0.002235993 | 0.030330536 |
| **EEF1A1P2** | 0.252416069 | 0.103394947 | -1.287638069 | 0.003798986 | 0.040265496 |
| **EEF1A1P42** | 0.171971903 | 0.066180189 | -1.377701557 | 0.002298051 | 0.030837684 |
| **EEF1B2P4** | 0.31265304 | 0.115727195 | -1.433834617 | 0.00141697 | 0.024159929 |
| **EMILIN3** | 1.176875085 | 0.425542597 | -1.467585741 | 0.000917965 | 0.019136873 |
| **FAM184A** | 0.334891055 | 0.107724967 | -1.636339192 | 0.000519467 | 0.013651417 |
| **FAM60BP** | 0.287646791 | 0.094836929 | -1.600777517 | 0.000689188 | 0.016252269 |
| **FAM60CP** | 0.35096957 | 0.136860459 | -1.358640262 | 0.002558989 | 0.03257229 |
| **FDPSP5** | 0.523715333 | 0.257076378 | -1.026585794 | 0.000641586 | 0.015543418 |
| **FEN1P1** | 0.211388514 | 0.080548437 | -1.391968491 | 0.004937227 | 0.046098126 |
| **FKBP4P1** | 0.254527189 | 0.107723093 | -1.240492213 | 0.000509015 | 0.013607365 |
| **FKBP4P6** | 0.245234902 | 0.115165165 | -1.090459929 | 0.002279685 | 0.030636202 |
| **GAPDHP66** | 0.216116419 | 0.070357193 | -1.619038846 | 0.000662294 | 0.015892783 |
| **GDA** | 0.303588361 | 0.062779895 | -2.273741968 | 0.001634832 | 0.025793273 |
| **GREB1L** | 0.281552311 | 0.098687248 | -1.512467404 | 0.00385616 | 0.040556728 |
| **GRM7-AS1** | 0.994429576 | 0.198633525 | -2.323760063 | 0.002012581 | 0.028760431 |
| **GS1-24F4.1** | 0.190354164 | 0.087920219 | -1.11441924 | 0.004095633 | 0.041709554 |
| **GS1-309P15.4** | 0.330499251 | 0.158942334 | -1.056143564 | 0.005323647 | 0.047894141 |
| **GSTP1P1** | 0.531804921 | 0.158892753 | -1.742843797 | 0.000355813 | 0.01093805 |
| **HAND2** | 1.003175093 | 0.354510465 | -1.500673312 | 0.000306423 | 0.010068524 |
| **HAUS6P1** | 0.217994998 | 0.092562308 | -1.23579828 | 7.52E-05 | 0.004452086 |
| **HCRT** | 0.616378144 | 0.118766577 | -2.37568681 | 0.002424254 | 0.031736625 |
| **HIST1H2BPS3** | 0.209002056 | 0.052296098 | -1.998741919 | 0.001030834 | 0.020215481 |
| **HK2P1** | 0.211524636 | 0.099209896 | -1.092269766 | 0.000311574 | 0.010162384 |
| **HMGN2P18** | 0.399914745 | 0.143310513 | -1.480548028 | 0.00183964 | 0.027503649 |
| **HNRNPA1P19** | 0.31511059 | 0.081650612 | -1.948322631 | 0.002214805 | 0.030279985 |
| **HNRNPA1P22** | 0.292929618 | 0.145244871 | -1.012066854 | 0.000207244 | 0.00795867 |
| **HNRNPA1P30** | 0.264862477 | 0.120362447 | -1.137858127 | 0.000732306 | 0.016962898 |
| **HNRNPA1P42** | 0.245760352 | 0.109503424 | -1.166276201 | 0.001870073 | 0.027560539 |
| **HNRNPA1P43** | 0.24661401 | 0.099307039 | -1.312286876 | 3.14E-05 | 0.002565048 |
| **HNRNPA1P54** | 0.26502048 | 0.050994571 | -2.37768827 | 2.61E-05 | 0.002315692 |
| **HNRNPA1P55** | 0.262202712 | 0.12700991 | -1.045741539 | 0.000580882 | 0.014749756 |
| **HNRNPA3P8** | 0.166006045 | 0.074948191 | -1.147270221 | 0.002588158 | 0.032926678 |
| **HRASLS2** | 0.571256869 | 0.278858444 | -1.034606651 | 0.003393913 | 0.037909143 |
| **HSP90AB7P** | 0.171641726 | 0.066834364 | -1.36073832 | 0.000400542 | 0.011687147 |
| **HSPA8P11** | 0.311585401 | 0.142299804 | -1.130693966 | 0.000296003 | 0.009806828 |
| **HSPD1P16** | 0.312544755 | 0.134263735 | -1.218993111 | 0.003147567 | 0.036450354 |
| **HSPD1P2** | 0.180593964 | 0.062141178 | -1.539128173 | 0.000101449 | 0.005168462 |
| **HSPD1P5** | 0.513971256 | 0.228680782 | -1.168352548 | 6.53E-07 | 0.000269918 |
| **HSPE1P7** | 0.649051328 | 0.221797773 | -1.549087695 | 0.000529978 | 0.01388353 |
| **HSPE1P9** | 0.186101531 | 0.048152252 | -1.950414756 | 0.00347555 | 0.038320637 |
| **ICE2P1** | 0.201058631 | 0.031716184 | -2.664325156 | 0.001248427 | 0.022789531 |
| **KBTBD11-OT1** | 0.344891208 | 0.083284216 | -2.050026349 | 1.25E-05 | 0.001488561 |
| **KCNJ11** | 0.390728063 | 0.111169335 | -1.813405993 | 0.005315771 | 0.047894141 |
| **KLHL7-AS1** | 0.46671531 | 0.221414991 | -1.07578989 | 0.000253243 | 0.00901357 |
| **LGR6** | 2.12753963 | 0.882497145 | -1.269522489 | 7.43E-05 | 0.004422857 |
| **LINC00837** | 0.698029254 | 0.118092257 | -2.563373123 | 0.001986508 | 0.02842661 |
| **LINC01060** | 1.484618422 | 0.723515012 | -1.036997321 | 0.001471019 | 0.024574436 |
| **LINC01517** | 0.536558744 | 0.099443631 | -2.431785246 | 0.001309091 | 0.023163996 |
| **MAP3K15** | 0.311851248 | 0.105278979 | -1.566640633 | 0.003391096 | 0.03789654 |
| **MIP** | 0.404037374 | 0.143359952 | -1.494846693 | 0.000756067 | 0.017361814 |
| **MIR181B1** | 0.528432667 | 0.249517703 | -1.082577475 | 0.00527992 | 0.047700426 |
| **MIR4444-2** | 1.016333809 | 0.447229851 | -1.184285932 | 0.000458187 | 0.0126719 |
| **MIR4666A** | 0.49185903 | 0.07362178 | -2.740040349 | 1.06E-05 | 0.001418611 |
| **MIR6859-3** | 0.731619917 | 0.273890957 | -1.417492718 | 0.002362516 | 0.031308381 |
| **MMP20** | 0.748760465 | 0.232306314 | -1.688475894 | 0.000551976 | 0.014323661 |
| **MT3** | 0.331921937 | 0.148959415 | -1.155924673 | 0.005670454 | 0.049461797 |
| **MTNR1A** | 0.156798759 | 0.048870213 | -1.681886846 | 0.001412037 | 0.024159929 |
| **MYEF2** | 0.956865366 | 0.417916912 | -1.195099806 | 0.003912653 | 0.040647022 |
| **MYO16** | 0.616720203 | 0.132841866 | -2.214906215 | 0.004438449 | 0.043659419 |
| **MYOM2** | 1.449531207 | 0.678266401 | -1.095662459 | 3.80E-07 | 0.000209348 |
| **NACAP5** | 0.25337001 | 0.083331641 | -1.60430948 | 0.002985848 | 0.035323284 |
| **NPM1P12** | 0.336374061 | 0.152831367 | -1.138125787 | 0.000977383 | 0.019639413 |
| **NPM1P18** | 0.45390058 | 0.222344729 | -1.029578128 | 0.000708091 | 0.016571339 |
| **NPM1P30** | 0.248859114 | 0.029864912 | -3.058805864 | 4.74E-06 | 0.000877724 |
| **NPM1P31** | 0.293483935 | 0.109292424 | -1.425088134 | 0.001678234 | 0.026242399 |
| **NPM1P34** | 0.247010504 | 0.092763867 | -1.412937524 | 0.002881903 | 0.03465973 |
| **NPM1P40** | 0.360934949 | 0.177147535 | -1.026787452 | 0.00395815 | 0.041083875 |
| **NPM1P50** | 0.256467527 | 0.125418671 | -1.032024033 | 0.002038869 | 0.028954806 |
| **NRBF2P3** | 0.340145385 | 0.15068635 | -1.174602778 | 0.0009023 | 0.018982709 |
| **OR8B10P** | 0.391702914 | 0.096870908 | -2.015624485 | 0.00118892 | 0.022010984 |
| **OR8B9P** | 0.415878967 | 0.149104853 | -1.479836507 | 0.002250034 | 0.030330536 |
| **OR8Q1P** | 0.398326107 | 0.1138631 | -1.806649757 | 0.002306646 | 0.030906588 |
| **OTX2** | 0.43218833 | 0.093052665 | -2.215540744 | 0.002308145 | 0.030906588 |
| **OXCT2** | 0.823386804 | 0.404227024 | -1.026404556 | 5.81E-05 | 0.003781155 |
| **PGAM1P12** | 0.174834754 | 0.054098163 | -1.692340491 | 0.00043734 | 0.012273233 |
| **PHBP8** | 0.302555333 | 0.141621363 | -1.095160111 | 0.000471654 | 0.012886347 |
| **PRDM13** | 0.373487689 | 0.0813569 | -2.198724079 | 0.004072056 | 0.041554737 |
| **PRR18** | 0.430813719 | 0.188909722 | -1.189367244 | 0.00473511 | 0.045372198 |
| **RIMKLA** | 0.314996546 | 0.126133819 | -1.320380866 | 0.004381073 | 0.043179755 |
| **RN7SKP85** | 0.270871975 | 0.039181195 | -2.789377846 | 0.003396259 | 0.037918266 |
| **RN7SL416P** | 0.202097157 | 0.049510678 | -2.029237421 | 0.004684825 | 0.045012055 |
| **RN7SL644P** | 0.414001851 | 0.153987581 | -1.426823214 | 0.001775416 | 0.026988071 |
| **RP1-290I10.2** | 0.183033338 | 0.027326959 | -2.743709646 | 3.61E-05 | 0.002777462 |
| **RP11-1074O12.1** | 0.235177472 | 0.080695202 | -1.543195069 | 0.005509546 | 0.048763256 |
| **RP11-108M9.3** | 0.508537579 | 0.131304983 | -1.953432717 | 0.00112704 | 0.02131067 |
| **RP11-1136G4.2** | 0.593240706 | 0.268527754 | -1.143546386 | 0.000375486 | 0.011208528 |
| **RP11-119F7.3** | 0.418341855 | 0.184062181 | -1.184489116 | 0.005465351 | 0.048718799 |
| **RP11-119H12.1** | 0.384676564 | 0.18628829 | -1.046108946 | 0.000147253 | 0.006320145 |
| **RP11-126F18.2** | 0.194024138 | 0.085603179 | -1.180499869 | 0.001948054 | 0.028255912 |
| **RP11-12K11.1** | 0.425486154 | 0.170203339 | -1.321852847 | 0.003050728 | 0.035727862 |
| **RP11-136I14.5** | 0.788259295 | 0.276052239 | -1.513728976 | 0.003002762 | 0.035506444 |
| **RP11-147K6.1** | 0.339146661 | 0.121486471 | -1.481113634 | 0.004787577 | 0.045401454 |
| **RP11-151H2.1** | 0.345697363 | 0.075743 | -2.190325136 | 0.001743174 | 0.026691668 |
| **RP11-16E18.1** | 0.187382248 | 0.079692605 | -1.233466514 | 0.001816915 | 0.027251532 |
| **RP11-16L9.1** | 0.610285274 | 0.183017787 | -1.737499912 | 6.49E-06 | 0.001088513 |
| **RP11-195C7.2** | 0.293075907 | 0.101726244 | -1.526582451 | 0.005387953 | 0.048098003 |
| **RP11-205M3.1** | 0.26648594 | 0.115865679 | -1.201606141 | 0.003282409 | 0.037370355 |
| **RP11-229A12.2** | 0.504763178 | 0.2375343 | -1.087470815 | 0.000887984 | 0.018876706 |
| **RP11-229M1.2** | 0.595155354 | 0.291013155 | -1.032181932 | 0.001041522 | 0.020271923 |
| **RP11-249C24.12** | 0.21495339 | 0.051067726 | -2.073540148 | 0.005151705 | 0.047090455 |
| **RP11-24H1.1** | 0.459869417 | 0.193752293 | -1.24701087 | 8.07E-05 | 0.004507046 |
| **RP11-256G5.1** | 0.248093982 | 0.080932905 | -1.616088449 | 0.001563733 | 0.025382317 |
| **RP11-25H11.1** | 0.42016655 | 0.146004335 | -1.52495011 | 4.25E-05 | 0.003127679 |
| **RP11-283C24.1** | 0.270220731 | 0.111285492 | -1.279872838 | 0.0002086 | 0.00799836 |
| **RP11-289K10.1** | 0.488068249 | 0.223848071 | -1.124563011 | 0.001733517 | 0.026691668 |
| **RP11-291H24.1** | 0.924740876 | 0.342446044 | -1.433172469 | 0.000494759 | 0.013312347 |
| **RP11-2F9.3** | 0.210991988 | 0.100802332 | -1.065659196 | 0.004145036 | 0.04183485 |
| **RP11-325E14.5** | 0.209837649 | 0.103109321 | -1.02509879 | 0.001588129 | 0.025475372 |
| **RP11-331H13.1** | 0.498438645 | 0.227905334 | -1.128981239 | 0.001575912 | 0.025475372 |
| **RP11-366O20.5** | 0.404620231 | 0.194261143 | -1.0585711 | 0.005246956 | 0.047495522 |
| **RP11-369K16.1** | 0.56681247 | 0.2048151 | -1.468549417 | 0.002089086 | 0.029413188 |
| **RP11-382N13.1** | 0.271874017 | 0.114075352 | -1.25295117 | 0.000228644 | 0.008478625 |
| **RP11-384B12.2** | 0.583250935 | 0.278647954 | -1.065673156 | 0.000286017 | 0.009653748 |
| **RP11-388B24.4** | 0.185236116 | 0.065874612 | -1.49157095 | 0.003820251 | 0.040397612 |
| **RP11-415C15.1** | 0.192719177 | 0.075672955 | -1.348650454 | 0.001048216 | 0.020363463 |
| **RP11-432J24.5** | 0.636648272 | 0.221797837 | -1.521251253 | 0.00499642 | 0.046528224 |
| **RP11-439C15.4** | 0.380971364 | 0.123250801 | -1.628085538 | 0.000284308 | 0.009609144 |
| **RP11-454H13.1** | 0.367899792 | 0.155941683 | -1.238306254 | 0.005645042 | 0.049461797 |
| **RP11-468H14.1** | 0.172543312 | 0.06920724 | -1.317963681 | 0.002462434 | 0.031963614 |
| **RP11-473P24.2** | 0.571731218 | 0.262728785 | -1.121762797 | 0.00014081 | 0.006154736 |
| **RP11-477G18.2** | 0.499976613 | 0.225012251 | -1.151857058 | 0.000630541 | 0.015472263 |
| **RP11-478H13.4** | 0.479104743 | 0.153019339 | -1.646627096 | 0.004959669 | 0.046284377 |
| **RP11-4M23.7** | 0.371178274 | 0.059864057 | -2.632350317 | 0.000831291 | 0.018221487 |
| **RP11-4N23.1** | 0.373269548 | 0.140097782 | -1.413783702 | 0.000627663 | 0.015440195 |
| **RP11-500M8.7** | 0.192196805 | 0.079576119 | -1.272176912 | 0.001447911 | 0.024253738 |
| **RP11-536C10.1** | 0.524827188 | 0.231840869 | -1.17870755 | 0.000832602 | 0.018221487 |
| **RP11-53I6.1** | 0.669342188 | 0.310656405 | -1.10742415 | 0.000177496 | 0.007173693 |
| **RP11-550C4.4** | 0.19836591 | 0.062674152 | -1.662221632 | 0.002355627 | 0.031233774 |
| **RP11-565A3.2** | 0.286947435 | 0.038071534 | -2.914001856 | 0.000177025 | 0.007173693 |
| **RP11-65M17.3** | 0.410433646 | 0.120211148 | -1.771578309 | 0.002251592 | 0.030330536 |
| **RP11-67L3.5** | 0.508712906 | 0.228632479 | -1.153821333 | 6.88E-06 | 0.001101706 |
| **RP11-680L20.1** | 0.173571984 | 0.058924398 | -1.558597081 | 0.002888174 | 0.034680451 |
| **RP11-69M1.3** | 0.300805768 | 0.119257562 | -1.334751488 | 0.001331301 | 0.023472481 |
| **RP11-69M1.4** | 0.36000994 | 0.127226771 | -1.500634463 | 2.46E-05 | 0.00224077 |
| **RP11-701I24.1** | 0.487093453 | 0.201875782 | -1.270730741 | 3.24E-05 | 0.002581181 |
| **RP11-708H21.1** | 0.237096367 | 0.040977446 | -2.532571576 | 0.000520455 | 0.013662897 |
| **RP11-713C19.2** | 0.758757384 | 0.366743062 | -1.04886898 | 4.99E-07 | 0.000229153 |
| **RP11-718G2.5** | 0.224772499 | 0.040977811 | -2.455550691 | 0.000106204 | 0.005251092 |
| **RP11-71L14.4** | 0.277359391 | 0.0863561 | -1.683386582 | 0.001049336 | 0.020369278 |
| **RP11-74K11.1** | 0.159821848 | 0.067103369 | -1.252007538 | 0.002691873 | 0.033443022 |
| **RP11-756P10.1** | 0.59665826 | 0.215756612 | -1.467500086 | 5.01E-05 | 0.00348869 |
| **RP11-80H5.2** | 0.219367627 | 0.05647879 | -1.957569558 | 0.000130269 | 0.005823817 |
| **RP11-84H6.1** | 0.236798396 | 0.111454085 | -1.08720982 | 5.89E-05 | 0.003821163 |
| **RP11-855A2.5** | 0.522392822 | 0.228076611 | -1.195616565 | 0.001602665 | 0.025684048 |
| **RP11-91A18.1** | 0.312283534 | 0.099025682 | -1.656981867 | 0.00533656 | 0.047951272 |
| **RP13-235E23.1** | 0.344561334 | 0.111311363 | -1.630159936 | 0.000415889 | 0.011936407 |
| **RP13-824C8.2** | 0.269571847 | 0.104875632 | -1.361990331 | 0.000947398 | 0.019335935 |
| **RP3-407E4.4** | 0.205307196 | 0.098129649 | -1.065023186 | 0.000854732 | 0.018502779 |
| **RP3-486D24.1** | 0.266496549 | 0.126853909 | -1.070948871 | 0.004447775 | 0.043716482 |
| **RP4-612B18.1** | 0.561846352 | 0.272684014 | -1.042945524 | 0.005593212 | 0.049309315 |
| **RP5-875O13.1** | 0.32727104 | 0.076658614 | -2.093966116 | 0.001661629 | 0.026040231 |
| **RPEL1** | 0.267414905 | 0.116027438 | -1.204613868 | 0.003695756 | 0.039606316 |
| **RPL10AP3** | 0.377008676 | 0.182447143 | -1.047119163 | 0.000649868 | 0.015698074 |
| **RPL15P21** | 0.144067889 | 0.056670711 | -1.346073592 | 0.000905532 | 0.018982709 |
| **RPL17P41** | 0.30340491 | 0.148014665 | -1.035504313 | 0.001647626 | 0.025853445 |
| **RPL17P44** | 0.145544237 | 0.058403817 | -1.317323147 | 0.000627621 | 0.015440195 |
| **RPL18AP8** | 0.174929987 | 0.076724535 | -1.189017723 | 0.005686272 | 0.049461797 |
| **RPL21P111** | 0.291582499 | 0.071012363 | -2.037762011 | 0.003260629 | 0.037173565 |
| **RPL21P127** | 0.173313976 | 0.061976102 | -1.48360407 | 4.86E-05 | 0.003434187 |
| **RPL22P18** | 0.390677215 | 0.165678451 | -1.237591144 | 0.00083813 | 0.018303099 |
| **RPL23AP78** | 0.329272285 | 0.091872315 | -1.841579002 | 0.000343398 | 0.010769948 |
| **RPL31P35** | 0.39061961 | 0.175240992 | -1.15642409 | 0.005368071 | 0.047951272 |
| **RPL35AP33** | 0.493106249 | 0.221248849 | -1.15622859 | 0.002964526 | 0.035234895 |
| **RPL5P32** | 0.371565139 | 0.180267182 | -1.043478374 | 0.000971586 | 0.019548335 |
| **RPL5P33** | 0.168824004 | 0.052647582 | -1.681080868 | 0.001871142 | 0.027560539 |
| **RPL7AP38** | 0.167348205 | 0.065124134 | -1.361588878 | 0.001844228 | 0.027503649 |
| **RPL7AP8** | 0.382731722 | 0.186361768 | -1.038227563 | 0.000978255 | 0.019639413 |
| **RPL7P33** | 0.344467281 | 0.162185304 | -1.086723855 | 0.000148908 | 0.006380157 |
| **RPL7P36** | 0.245714903 | 0.114289393 | -1.104293851 | 0.000604038 | 0.015060266 |
| **RPL7P58** | 0.304355352 | 0.140185514 | -1.118419459 | 0.002826325 | 0.034353492 |
| **RPRML** | 0.318346259 | 0.100086988 | -1.669342378 | 0.002497106 | 0.032214354 |
| **RPS10-NUDT3** | 0.151624491 | 0.052143193 | -1.539951965 | 0.001408218 | 0.024126432 |
| **RPS24P18** | 0.353071092 | 0.148771384 | -1.246861652 | 0.001953498 | 0.028307467 |
| **RPS24P6** | 0.481019594 | 0.228539664 | -1.073651093 | 0.000627989 | 0.015440195 |
| **RPS26P55** | 0.485106183 | 0.193838892 | -1.323442507 | 0.003304353 | 0.037534059 |
| **RPS26P56** | 0.440841795 | 0.168616968 | -1.386511282 | 0.000247234 | 0.00892298 |
| **RPS2P2** | 0.527447937 | 0.254786897 | -1.049737607 | 0.000113101 | 0.005477313 |
| **RPS3AP40** | 0.241599151 | 0.094137565 | -1.359772941 | 0.000954716 | 0.019461464 |
| **RPS3AP9** | 0.179100582 | 0.020985294 | -3.093319462 | 0.000512114 | 0.013651417 |
| **RPS7P7** | 0.319990883 | 0.125542978 | -1.349849465 | 0.001755297 | 0.026764227 |
| **SCN5A** | 0.595301276 | 0.221689814 | -1.4250775 | 0.001076228 | 0.020664911 |
| **SETP7** | 0.200895904 | 0.054774777 | -1.874864528 | 0.003977614 | 0.041083875 |
| **SLC25A5P6** | 0.374520536 | 0.15049794 | -1.315301088 | 0.000911425 | 0.019064607 |
| **SLC25A5P7** | 0.274840037 | 0.133819448 | -1.038304385 | 0.002348559 | 0.031190066 |
| **SLC27A2** | 0.763037648 | 0.287405729 | -1.408665421 | 0.001587817 | 0.025475372 |
| **SLC6A2** | 0.916406348 | 0.325521157 | -1.493236141 | 0.003096961 | 0.036070139 |
| **SLC9A2** | 0.725803212 | 0.337517358 | -1.104616741 | 0.001764783 | 0.026892071 |
| **SNRPEP3** | 0.235308517 | 0.070644604 | -1.735902267 | 4.79E-05 | 0.003402184 |
| **SNRPEP5** | 0.470094671 | 0.210762956 | -1.157330007 | 0.002817948 | 0.034268456 |
| **SNRPEP6** | 0.275120226 | 0.110451254 | -1.316652411 | 0.000995173 | 0.019828823 |
| **TMEM151A** | 0.293460454 | 0.122809075 | -1.256748935 | 0.003063322 | 0.035727862 |
| **TP73** | 0.659064729 | 0.141238395 | -2.222287836 | 6.85E-05 | 0.00419579 |
| **TUBB4BP3** | 0.456231671 | 0.168600102 | -1.43616119 | 0.004325478 | 0.043094959 |
| **VDAC1P5** | 0.179609886 | 0.069014707 | -1.379891018 | 0.000905979 | 0.018982709 |
