# Supplementary material for: A three-gene signature based on tumour microenvironment predicts overall survival of osteosarcoma in adolescents and young adults
Source: Aging (Albany NY). 2020 Dec 3;13(1):619–45. doi: 10.18632/aging.202170 (PMC7835013; doi:10.18632/aging.202170)
Supplement: Supplementary Tables 4, 5 and 6 [file aging-13-202170-s005.pdf]

SUPPLEMENTARY TABLES

Supplementary Table 4. The intersection of differential genes between high and low score groups.

Gene	Regulation
CCL14	up
CCR2	up
FCGR1A	up
FCGR1B	up
FCGR1C	up
FCGR2B	up
GAPT	up
GIMAP5	up
IGHG2	up
IGHM	up
IGHV1-24	up
IGKV3-11	up
IL10	up
IL2RA	up
LILRA1	up
LILRA2	up
LLNLR-470E3.1	up
MS4A14	up
P2RY10	up
PLD4	up
RP11-24F11.2	up
RP11-455F5.5	up
RP11-494O16.3	up
RP11-733O18.1	up
RP11-84C10.2	up
TLR7	up
TLR8	up
TNFSF8	up
TRBV29-1	up
TRBV7-3	up
VENTX	up
AC003988.1	down
AC013470.6	down
COCH	down
EMILIN3	down
FAM60BP	down
GS1-309P15.4	down
HAUS6P1	down

HK2P1	down
MAP3K15	down
RP1-290I10.2	down
RP11-283C24.1	down
RPRML	down

Supplementary Table 5. Screening of genes affecting prognosis of osteosarcoma patients by univariate Cox regression analysis.

Id	HR	HR.95 L	HR.95 H	P value
ARHGAP9	0.51087512	0.279370094	0.934220925	0.029190049
ARL11	0.2925651	0.106229156	0.805751841	0.017415939
CARD11	0.366473556	0.135386517	0.991995883	0.048179537
CCL2	0.554934323	0.361183981	0.852618385	0.007196217
COCH	1.444447813	1.154257554	1.807594396	0.001310445
COLEC10	2.165998823	1.213980939	3.864600135	0.008886882
DENND1C	0.40798314	0.170180199	0.978082315	0.044469242
FCGR2B	0.330313897	0.122811646	0.888411433	0.028209966
GPR65	0.367016467	0.149575271	0.9005572	0.028620701
HSPD1P5	5.996700537	1.928439911	18.64741397	0.00197144
HTR2B	0.173056522	0.037741629	0.793515297	0.023968604
IGHG2	0.695364235	0.487357095	0.992150157	0.045132177
IL2RA	0.247279044	0.077194996	0.792109969	0.018656486
ITGAM	0.472112561	0.266834219	0.835313669	0.009935059
KBTBD11-OT1	3.569424862	1.869198572	6.816179958	0.000115665
LAG3	0.438454408	0.197341701	0.974159375	0.042945491
LILRB2	0.516296389	0.28565598	0.933157295	0.028593342
LILRB4	0.560448573	0.358927426	0.875114524	0.010874652
LRRC25	0.629703223	0.399797335	0.991817892	0.045998229
MIR4666A	2.388143135	1.302508685	4.378648448	0.004886248
MS4A4A	0.609618914	0.409457223	0.907628928	0.014799605
MYOM2	1.673891374	1.229811511	2.278326645	0.001056289
NCF1B	0.256873893	0.086789752	0.760276362	0.014088033
NCF4	0.630684051	0.422394571	0.941684386	0.024212517
PCED1B	0.373265768	0.164832238	0.845267499	0.01812482
PDE1B	0.128492357	0.028553448	0.578223886	0.007499985
PIK3R5	0.345663883	0.141728729	0.843043756	0.019527423
RASGRP4	0.262925485	0.098455778	0.702140718	0.007685921
SNX20	0.267559459	0.087584958	0.817355696	0.020671466
TNFSF8	0.256172732	0.088599698	0.740685017	0.011933582
TRBV7-3	0.061055107	0.004092557	0.910855004	0.042593103
VSIG4	0.663373939	0.489320843	0.899338314	0.008210926

Supplementary Table 6. Immune-related differential genes in high and low risk group.

Gene	Low risk	High risk	Log FC	P Value	FDR
C3	2.101113494	0.993065337	-1.081193548	3.42E-06	0.000164068
CCL23	0.163230344	0.078820756	-1.050261781	0.007313622	0.035887774
CD3D	1.257976912	0.581421327	-1.113449547	3.45E-05	0.000757924
CD3E	1.234124531	0.559110455	-1.142282751	2.65E-06	0.000162902
CNTFR	0.763638404	1.740009475	1.188133599	0.008324904	0.039384637
COLEC10	0.116194113	0.342232389	1.558439322	3.88E-05	0.000805077
CSPG5	0.331461945	0.664455344	1.003329	5.66E-06	0.000232374
CX3CR1	1.310084234	0.647589283	-1.016508559	0.000112751	0.001950043
CXCR3	0.864951829	0.425292057	-1.024165878	1.88E-05	0.000505711
CXCR6	0.42387118	0.211257015	-1.004626627	0.000203797	0.003205921
FCGR2B	0.925072469	0.353461012	-1.388015301	6.26E-09	4.34E-06
IGHM	1.600302821	0.779517085	-1.03769238	0.004409497	0.027274265
IL2RA	0.770377786	0.294716765	-1.386236974	3.42E-06	0.000164068
PAK6	0.013952663	0.047304117	1.761425248	0.005420618	0.02981382
PRLHR	0.015912746	0.035923823	1.174758031	0.009123249	0.041138437
PROK2	0.357443064	0.932191954	1.382913603	0.001419738	0.011887491
PTHLH	0.467021326	0.15219946	-1.617525187	0.000558506	0.006400523
RETN	0.403612052	0.074929169	-2.429369894	0.000337595	0.004686353
RNASE2	1.131581553	0.441860267	-1.356678453	3.89E-05	0.000805077
TLR7	1.040862022	0.519912736	-1.001437434	2.79E-06	0.000162902
TRAV1-2	0.179271881	0.061862702	-1.535007475	0.000217345	0.003229565
TRAV14DV4	0.209995614	0.055742782	-1.913502272	0.000922779	0.008931487
TRBC2	1.572979597	0.750764535	-1.067067551	7.74E-05	0.001432748
TRBV12-4	0.278282339	0.138663227	-1.00496411	0.005634189	0.030482407
TRBV20-1	0.625931807	0.136296403	-2.199257999	1.42E-05	0.00039359
TRBV28	0.980494657	0.45568394	-1.105476244	0.000227234	0.003329603
TRBV29-1	0.230535009	0.09849979	-1.226793302	0.005011945	0.029217714
TRBV4-2	0.288890712	0.060070621	-2.265792342	0.000564217	0.006400523
TRBV6-5	0.302632378	0.119943755	-1.335208298	0.007216178	0.035887774
TRBV7-3	0.259605434	0.063285289	-2.0363785	8.82E-06	0.00026592
TRDC	1.285825752	0.538065431	-1.256841623	0.000919254	0.008931487
